# *Chlamydia trachomatis* and *Chlamydia muridarum* spectinomycin resistant vectors and a transcriptional fluorescent reporter to monitor conversion from replicative to infectious bacteria

**DOI:** 10.1371/journal.pone.0217753

**Published:** 2019-06-06

**Authors:** María Eugenia Cortina, Rachel J. Ende, R. Clayton Bishop, Charlie Bayne, Isabelle Derré

**Affiliations:** Department of Microbiology, Immunology and Cancer Biology, University of Virginia, Charlottesville, VA, United States of America; Institut Pasteur, FRANCE

## Abstract

*Chlamydia trachomatis* infections are the leading cause of sexually transmitted infections of bacterial origin. Lower genital tract infections are often asymptomatic, and therefore left untreated, leading to ascending infections that have long-term consequences on female reproductive health. Human pathology can be recapitulated in mice with the mouse adapted strain *C*. *muridarum*. Eight years into the post-genetic era, significant advances to expand the *Chlamydia* genetic toolbox have been made to facilitate the study of this important human pathogen. However, the need for additional tools remains, especially for *C*. *muridarum*. Here, we describe a new set of spectinomycin resistant *E*. *coli-Chlamydia* shuttle vectors, for *C*. *trachomatis* and *C*. *muridarum*. These versatile vectors allow for expression and localization studies of *Chlamydia* effectors, such as Inc proteins, and will be instrumental for mutant complementation studies. In addition, we have exploited the differential expression of specific *Chlamydia* genes during the developmental cycle to engineer an *omcA*::*gfp* fluorescent transcriptional reporter. This novel tool allows for monitoring RB to EB conversion at the bacterial level. Spatiotemporal tracking of GFP expression within individual inclusions revealed that RB to EB conversion initiates in bacteria located at the edge of the inclusion and correlates with the time post initiation of bacterial replication and inclusion size. Comparison between primary and secondary inclusions potentially suggests that the environment in which the inclusions develop influences the timing of conversion. Altogether, the *Chlamydia* genetic tools described here will benefit the field, as we continue to investigate the molecular mechanisms underlying *Chlamydia*-host interaction and pathogenesis.

## Introduction

*Chlamydia* spp. are Gram-negative obligate intracellular bacterial pathogens that infect a wide range of hosts and are responsible for various diseases. *Chlamydia trachomatis* is the leading cause of sexually transmitted infections of bacterial origin and the most common cause of non-congenital blindness due to trachoma [1]. Infection with *C*. *pneumoniae* leads to community-acquired pneumonia [2]. *C*. *felis*, *C*. *caviae*, and *C*. *suis* are among *Chlamydia* species that infect animals [3]. Of interest to this study, the mouse-adapted strain *C*. *muridarum* is commonly used to model human female genital tract infections, because intravaginal *C*. *muridarum* infection in mice recapitulates the upper genital tract pathology observed with *C*. *trachomatis* infection in women [4].

*Chlamydia* primarily infects the epithelium of the targeted tissue and replicates within a cytosolic membrane bound compartment, called the inclusion. In the lumen of the inclusion, *Chlamydia* undergoes a bi-phasic developmental cycle that is shared by all species and lasts 2–3 days. This cycle is characterized by alternation between two very distinct morphological and functional forms of the bacterium: a small, electron-dense, infectious elementary body (EB) and a larger, more electron-lucent, replicative reticulate body (RB) [5, 6]. The infection starts with the attachment and internalization of EBs, followed by EB conversion into RB. RBs replicate to high number and, beginning mid-cycle, start their asynchronous conversion back to infectious EBs [7]. At the end of the cycle, inclusions contain a large number of EBs that are released by successive lysis of the inclusion and host plasma membranes or by extrusion [8].

A prolific developmental cycle relies on the maturation of the inclusion into an environment conducive to *Chlamydia* replication. This process depends on the translocation of *Chlamydia* Type III effector proteins [9], some of which, the Inc proteins, are inserted into the inclusion membrane [10–12]. *Chlamydia* species encode 50 to 100 Inc proteins, all of which are characterized by one or more bi-lobed transmembrane domain and cytosolic tails [13]. The inclusion localization and topology of the Inc proteins makes them ideally positioned to mediate inclusion-host cell interactions.

The lack of genetic tools to manipulate *Chlamydia* has significantly hindered our ability to study the *Chlamydia* developmental cycle and characterize the role of *Chlamydia* virulence factors, including Inc proteins. A major breakthrough came from the development of a transformation method for *C*. *trachomatis* [14] and *E*. *coli-C*. *trachomatis* shuttle vectors allowing for fluorescent proteins and tagged-effector expression under constitutive or inducible promoters [14–20], as well as vectors for insertional targeted mutagenesis [21] or allelic replacement [22, 23]. These new genetic tools have been instrumental for live imaging of the *C*. *trachomatis* inclusion and to get a better understanding of the function of some of *C*. *trachomatis* Inc proteins [17, 20, 24–33].

In spite of these tremendous advances in the field, the *Chlamydia* genetic toolbox still has some limitations. Most of the shuttle vectors described for *C*. *trachomatis*, as well as *C*. *muridarum*, confer resistance to ampicillin, complicating complementation studies of ampicillin resistant mutant strains. Another limitation in the field is that genetic tools for *Chlamydia* species other than *C*. *trachomatis* are significantly underdeveloped. Due to species barrier specificity for replication, *C*. *trachomatis*-derived shuttle vectors cannot be maintained in other *Chlamydia* species [34, 35]. Species-specific vectors are therefore needed. Recently, Shima et al. reported the successful transformation and maintenance of a shuttle vector in *C*. *pneumoniae* [36]. Interestingly, this *C*. *pneumoniae*-derived vector also replicated in *C*. *felis*, suggesting that species barrier could be overcome in some instances. Multiple *C*. *muridarum* vectors have been described and successfully maintained in *C*. *muridarum*. These vectors were used to study the role of *C*. *muridarum* proteins encoded by the plasmid or for bioluminescence imaging of ascending infection in whole animals, using a luciferase reporter [34, 35, 37–39]. However, expression of transgenes has been limited to GFP or luciferase, and plasmid-based expression of effector proteins, such as Inc proteins, has never been performed in *C*. *muridarum*.

With the advance in *Chlamydia* genetics also comes the opportunity to create tools to monitor RB to EB conversion. Changes in the transcriptional profile of the bacteria as they progress through the developmental cycle have previously been reported [40, 41]. Some genes are expressed early and throughout, while others are expressed mid- to late-cycle as RBs begin to convert to EBs. The *omcAB* operon falls in the latter category. Transcription from this promoter is not initiated until mid-cycle and leads to the production of two cysteine-rich outer membrane proteins that are specific to EBs [40, 42–46]. Such temporal regulation of *Chlamydia* gene transcription offers the possibility to engineer a fluorescent transcriptional reporter indicative of RB to EB conversion at the bacterial level. This reporter would combine, in a single fluorescence assay, the power of methods that currently must be used in combination to accurately monitor the progression of the *Chlamydia* developmental cycle (i.e. inclusion size by immunofluorescence microscopy, bacterial morphology by electron microscopy and production of infectious progeny by Inclusion Forming Units (IFUs) assay). In addition, such a reporter would allow tracking of RB to EB conversion and could validate some of the models that have been proposed to control this process [7, 47].

Here, we describe a new set of spectinomycin resistant *E*. *coli-Chlamydia* shuttle vectors, for *C*. *trachomatis* and *C*. *muridarum*. These versatile vectors allow for expression and localization studies of *Chlamydia* effectors, such as Inc proteins, and will be instrumental for mutant complementation studies as the field further investigates the molecular mechanisms underlying *Chlamydia*-host interaction and pathogenesis. In addition, we have exploited the differential expression of specific *Chlamydia* genes during the developmental cycle to engineer an *omcA*::*gfp* fluorescent transcriptional reporter. This novel tool allows for monitoring RB to EB conversion at the bacterial level and will be a definite asset to monitor *Chlamydia* developmental growth defects, including in high-throughput assays. Moreover, spatiotemporal tracking of inclusions revealed that RB to EB conversion initiates in bacteria located at the edge of the inclusion and correlates with time post initiation of bacterial replication and inclusion size. In addition, comparison between primary and secondary inclusions potentially suggests that the environment in which the inclusions develop influences the timing of conversion.

## Material and methods

### Ethics statement

All genetic manipulations and containment work were approved by the UVA Biosafety Committee and are in compliance with the section III-D-1-a of the National Institutes of Health guidelines for research involving recombinant DNA molecules.

### Cell lines and bacterial strains

HeLa cells were obtained from ATCC (CCL-2) and cultured at 37°C with 5% CO_2_ in DMEM high glucose (Invitrogen) supplemented with 10% heat inactivated FBS (Invitrogen). *C*. *trachomatis Lymphogranuloma venereum*, *Type II* were obtained from ATCC (L2/434/Bu VR-902B). *C*. *muridarum* was obtained from Michael Starnbach (Harvard Medical School, Boston, MA). *Chlamydia* propagation and infection were performed as previously described [48].

### Plasmid construction

Restriction enzymes and T4 DNA ligase were obtained from New England Biolabs (Ipswich, MA). PCR was performed using Herculase DNA polymerase (Stratagene). PCR primers were obtained from Integrated DNA Technologies and are listed in S1 Table. All inserts were sequenced using Genscript. Plasmid maps were generated using Serial Cloner (http://serialbasics.free.fr/Serial_Cloner.html).

#### Construction of p2TK2_Spec_

A spectinomycin resistance cassette (Spec^R^) was assembled by overlap PCR using MCSIncDPromFw and aadA-TermOrigRv primers and the following templates: the *incDEFG* operon promoter, amplified from p2TK2-SW2 IncDProm-mCherrry-IncDTerm [15] with IncDPromNot5 and IncDPromaad3 primers, the aadA open reading frame (ORF), amplified from pDFTT3aadA incA plasmid [49] with IncDPromaad5 and aadTerm3 primers and a terminator [50], amplified with aadTerm5 and TermNot3 primers. In parallel, a DNA fragment corresponding to the *E*. *coli* origin of replication and the multiple cloning sites (Ori-MCS) was amplified from the p2TK2_Amp_ vector [15] with aadA-TermOrigFw and MCSIncDPromRv primers. p2TK2_Spec_ was constructed by overlap PCR of Ori-MCS and (Spec^R^) with MCSIncDPromFw and MCSIncDPromRv primers.

#### Construction of p2TK2_Spec_-SW2

pSW2 was obtained by BamHI digest of p2TK2-SW2 mCh(Gro) Tet-IncV-3F plasmid [29] and gel purification of the 7kb band corresponding to pSW2. To construct p2TK_Spec_-SW2, BamHI digested pSW2 was cloned into the BamHI site of p2TK2_Spec_.

#### Construction of p2TK2_Spec_-SW2 mCh(Gro_L2_)

The DNA fragment corresponding to the *mCherry* ORF, flanked by the *groESL* operon promoter and terminator was obtained by AgeI digest of p2TK2-SW2 mCh(Gro) Tet-IncV-3F plasmid [29], gel purified and cloned into the AgeI site of p2TK2_Spec_-SW2.

#### Construction of p2TK2_Spec_-SW2 mCh(Gro_L2_) MCS-3xFLAG

A DNA fragment corresponding to the 3xFLAG and the *incDEFG* operon terminator was amplified by PCR from p2TK2-SW2 mCh(Gro) Tet-IncV-3F plasmid [29] using primers NotI3XFLAGFw and IncDTermRv and cloned into the NotI/SalI sites of p2TK2_Spec_-SW2 mCh(Gro_L2_).

#### Construction of p2TK2_Spec_-SW2 mCh(Gro_L2_) Tet-IncV-3xFLAG

A DNA fragment corresponding to the *tet* repressor (TetR), *tetA* promoter (*tetA*^*P*^) and *incV* ORF was amplified by PCR from p2TK2-SW2 mCh(Gro) Tet-IncV-3F plasmid [29] using primers TetRSTOP5Kpn and IncVNotIRv and cloned into the KpnI/NotI sites of p2TK2_Spec_-SW2 mCh(Gro_L2_) MCS-3xFLAG.

#### Construction of p2TK2_Spec_-SW2 mCh(Gro_L2_) Tet-KpnI-IncV-NotI-3xFLAG

A DNA fragment corresponding to the *tet repressor* and the *tetA* promoter was amplified by PCR from p2TK2-SW2 mCh(Gro) Tet-IncV-3F plasmid [29] using primers AgeITetRStopFw and TetAPKpnIRv. The *incV* gene was amplified by PCR from the same template with the KpnIIncVFw and IncVNotIRv primers. These PCR products were sequentially cloned into the AgeI/KpnI and KpnI/NotI sites, respectively, of p2TK2_Spec_-SW2 mCh(Gro_L2_) MCS-3xFLAG.

#### Construction of p2TK2_Spec_-Nigg

The cloning strategy was inspired by and used plasmids generated by Wang et al. and Skilton et al. [35, 39]. The 7kb BamHI fragment of pSW2NiggCDS2, corresponding to SW2 (CDS3-8 and CDS1) but in which the SW2 CDS2 ORF has been substituted for the Nigg CDS2 ORF [35], was cloned into the BamHI restriction site of p2TK2_Spec_, leading to p2TK2_Spec_-SW2NiggCDS2. Then, a ~6.3kb SpeI/MluI fragment from pGFP::Nigg, corresponding to a portion of CDS2 and CDS3-8 from Nigg [39], was cloned into the ~3kb SpeI/MluI fragment from p2TK2_Spec_-SW2NiggCDS2, corresponding to p2TK2_Spec_, a portion of Nigg CDS2 and SW2 CDS1. The resulting ligation led to p2TK2_Spec_-Nigg. Please note that as for pGFP::Nigg [39], our *E*. *coli*-*C*. *muridarum* shuttle vector encode all Nigg CDS, except for CDS1 which is from the *C*. *trachomatis* SW2 strain.

#### Construction of p2TK2_Spec_-Nigg mCh(Gro_L2_)

The DNA fragment corresponding to the *mCherry* ORF, flanked by the *groESL* operon promoter and terminator was obtained by AgeI digest of p2TK2_Spec_-SW2 mCh(Gro_L2_) and gel purification of the corresponding ∼1kb fragment (mCh(Gro_L2_)). To construct p2TK2_Spec_-Nigg mCh(Gro_L2_), AgeI digested mCh(Gro_L2_) was cloned into the AgeI restriction site of p2TK2_Spec_-Nigg.

#### Construction of p2TK2_Spec_-Nigg mCh(Gro_L2_) TetTC0273-3xFLAG

A DNA fragment corresponding to the TetR-tetA^P^ regulatory element was amplified by PCR from p2TK2_Spec_-SW2 mCh(Gro_L2_) Tet-IncV-3xFLAG using primers TetRSTOP5Kpn and TetTC02733. A DNA fragment corresponding to the *tc0273* ORF was amplified from *C*. *muridarum* genomic DNA by PCR using primers TetTC02735 and 0273FLAG3. A DNA fragment corresponding to 3xFLAG *incDEFG* terminator was amplified by PCR from p2TK2_Spec_-SW2 mCh(Gro_L2_) Tet-IncV-3xFLAG using primers 0273FLAG5 and IncDTerm3NotI. A DNA fragment corresponding to TetR-tetA^P^ TC0273 3xFLAG *incDEFG* terminator was amplified by overlapping PCR and cloned into the KpnI/NotI sites of p2TK2_Spec_-Nigg mCh(Gro_L2_).

#### Construction of p2TK2-SW2 mCh(Gro_L2_) GFP(OmcA_L2_)

A DNA fragment corresponding to the intergenic region upstream of the *omcAB* operon was amplified by PCR from *C*. *trachomatis* L2 genomic DNA using primers OmcAProm5Kpn and OmcARSGFP3. A DNA fragment corresponding to GFP and *incDEFG* terminator was amplified from p2TK2-SW2 IncDProm-RSGFP-IncDTerm [15] using primers OmcARSGF5 and IncDTerm3NotI. A DNA fragment corresponding to *omcA* promoter-GFP-*incDEFG* terminator was amplified by overlapping PCR and cloned into the KpnI/NotI sites of p2TK2-SW2 mCh(Gro_L2_) [15]. Please note that this plasmid confers resistance to ampicillin.

#### Construction of p2TK2_Spec_-Nigg mCh(Gro_L2_) GFP(OmcA_Cm_)

A DNA fragment corresponding to the intergenic region upstream of the *omcAB* operon was amplified by PCR from *C*. *muridarum* genomic DNA using primers CmOmcAProm3KpnI and CmOmcApromRSGFP3. A DNA fragment corresponding to GFP and the *C*. *trachomatis incDEFG* terminator was amplified from p2TK2-SW2 mCh(Gro_L2_) GFP(OmcA_L2_) using primers CmOmcApromRSGF5 and IncDTerm3NotI. A DNA fragment corresponding to *omcA*_*Cm*_ promoter-GFP-*incDEFG* terminator was amplified by overlapping PCR and cloned into the KpnI/NotI restriction sites of p2TK2_Spec_-Nigg mCh(Gro_L2_).

### *C*. *trachomatis* and *C*. *muridarum* transformation

Our calcium-based transformation protocol was adapted from Wang et al. [14] and modified from Agaisse et al. [15] as follows. For one transformation the reaction was set by combining 500ng of plasmid DNA (extracted from the *E*.*coli* GM2163 (dam^-^ dcm^-^) strain), *C*. *trachomatis L2* or *C*. *muridarum* (empirically determined so that 100% of the cells were infected), 6.7μl of 5X CaCl_2_ Buffer (50mM Tris, 250mM CaCl_2_ pH 7.4), and water to a final volume of 33.5μl, added in that order and incubated for 30min at room temperature. Mixing the reaction by pipetting up and down every 5min can be performed but is not necessary. At the end of the 30min, 2ml of media (DMEM high glucose supplemented with 10% FBS) was added to the transformation reaction and transferred to one well of a 6 well plate containing a confluent monolayer of HeLa cells (5x10^5^ cells plated the day before). The plate was centrifuged for 10–15 min at 1,200 rpm and incubated for 36-48h at 37°C in the presence of 5% CO_2_. At 12-18h post-infection (p.i.), the media was removed and 2ml of fresh media supplemented with 500μg/ml spectinomycin (Fisher BioRegent) and 1μg/ml of cycloheximide (Sigma) was added. The first passage was performed at 36-48h p.i. (36h p.i. was favored for *C*. *muridarum*). The media was removed and the infected cells were lysed by addition of 0.5ml of water/well, scraped, collected and spun for 5min at 1,200rpm. The supernatant was diluted in 2ml of media containing 500μg/ml spectinomycin and 1μg/ml cycloheximide and added to a fresh cell monolayer (seeded the day before) and incubated for 48 h at 37°C in the presence of 5% CO2. Passages were performed until enough infectious particles were recovered to generate a frozen stock and proceed to plaque purification. For both *C*. *trachomatis* and *C*. *muridarum*, transformants were routinely observed at the second passage and occasionally observed after the first passage.

### Immunoblotting

Protein samples were separated by SDS-PAGE and transferred to nitrocellulose membranes. The membranes were blocked for 1h at room temperature in 1xPBS containing 0.05% Tween and 5% fat-free milk. Primary and HRP-conjugated secondary antibodies were diluted in 1xPBS containing 0.05% Tween and 5% fat-free milk and respectively incubated overnight at 4°C and 1h at room temperature. Proteins were detected using the Amersham ECL immunoblotting detection reagent as per manufacturer recommendation and a Bio-Rad ChemiDoc imaging system.

### Immunofluorescence and microscopy

At the indicated times, HeLa cells seeded onto glass coverslips were fixed for 30 min in PBS containing 4% paraformaldehyde. Immunostainings were performed at room temperature. Antibodies were diluted in PBS containing 0.1% Triton X-100. Samples were washed with PBS and examined under an epifluorescence or spinning disc confocal microscope. Images were processed using the Imaris software (Bitplane, Belfast, United Kingdom).

### Antibodies

The following primary antibodies were used: mouse monoclonal anti-FLAG (1:1,000 (IF), 1:10,000 (WB), Sigma), rabbit polyclonal anti-GFP (1:2,000, Molecular Probes), rabbit polyclonal anti-mCherry (1:2,000, BioVision), and rabbit polyclonal anti-actin (1:10,000, Sigma). The rabbit polyclonal anti-OmcA was generated against a synthetic OmcA peptide (CGGDTHQDAEHGPQARE) by Alpha Diagnostic International and used at 1:10,000. The following secondary antibodies were used: peroxidase-conjugated goat anti-rabbit IgG (1:10,000, Jackson ImmunoResearch), peroxidase-conjugated goat anti-mouse IgG (1:10,000, Jackson ImmunoResearch), and goat anti-mouse AlexaFluor 488 (1:500, Invitrogen).

### Time-lapse video microscopy

HeLa cells were seeded in 8 well Lab-Tek II Chambered Coverglass dishes (MatTek, Ashland, MA) and infected with the indicated fluorescent *C*. *trachomatis* or *C*. *muridarum* strain at a multiplicity of infection of 0.2. At 8h p.i. and for the next 70h, images were captured every 20min on a Leica DMi8 confocal microscope equipped with the Andor iXon ULTRA 888BV EMCCD camera, CSU-W1 confocal scanner unit, and a humidified live cell environmental chamber set at 37°C and 5% CO_2_. The Imaris imaging software (Bitplane, Belfast, United Kingdom) was used to analyze and process the data. Videos were converted to mp4 format using the HandBrake compression platform.

### Spatiotemporal analysis of RB to EB transition in *C*. *trachomatis*

Confocal time lapse videos were analyzed using the Imaris imaging software (Bitplane, Belfast, United Kingdom) by tracking individual inclusions in space and time. Quantification was performed on three independent confocal time lapse imaging experiments. A total of 46 primary inclusions and 29 secondary inclusions were analyzed. Secondary inclusions were distinguished from primary inclusions based on time of inclusion appearance and the timing of primary inclusion lysis. The graphs were generated using GraphPad Prism. A Student’s *t* test was performed and statistical significance was set to *P* < 0.0001.

*Quantification of time to GFP Expression*: The frame in which an inclusion containing 4–6 mCherry-positive RBs was clearly identifiable and the frame in which GFP expression is first visible within that same inclusion were identified. The 20-minute frame rate was then used to calculate the time elapsed between 4–6 mCherry-positive RBs and the start of GFP expression.

*Quantification of inclusion volume at time of GFP expression*: For each inclusion, at the time of first GFP expression, a single three-dimensional object corresponding to the raw mCherry signal of the bacteria was generated. The volume of the inclusion was calculated as the sum of the pixels within the 3D object.

*Tracking of the spatial distribution of the GFP signal over time*: Individual inclusions were tracked for 120min after GFP expression was first visible. At each 20min time mark, the spatial distribution of the GFP signal within individual inclusions was determined by analyzing individual Z stacks of covering the entire inclusion and recording if the signal was at the edge of the inclusion (edge), within the inclusion lumen (internal), or at both the edge and lumen (edge and internal). The mCherry signal of the bacteria was used to determine the boundaries of the inclusion.

## Results

### A new set of spectinomycin resistant cloning vectors for *Chlamydia trachomatis*

Following the development of a transformation system for *Chlamydia* [14], we described a series of p2TK2_Amp_-SW2 *E*. *coli-C*. *trachomatis* shuttle cloning vectors [15, 17]. These plasmids encode an ampicillin resistance cassette. Based on a report by Lowden et al. that spectinomycin could also be used to select *C*. *trachomatis* transformants [49], we generated a new series of p2TK2-SW2 plasmids. The simplest vector of this series is referred to as p2TK2_Spec_-SW2. It is very similar to p2TK2_Amp_-SW2 [15], except that it encodes the aminoglycoside 3′ adenyltransferase gene (*aadA*), which confers resistance to spectinomycin. The *incDEFG* promoter, which is expressed throughout the developmental cycle [40], was chosen to drive the expression of the *aadA* gene to ensure the constitutive expression of the antibiotic resistance. The presence of a multiple cloning site (MCS) facilitates the cloning of any transgene of interest.

**Fig 1:**
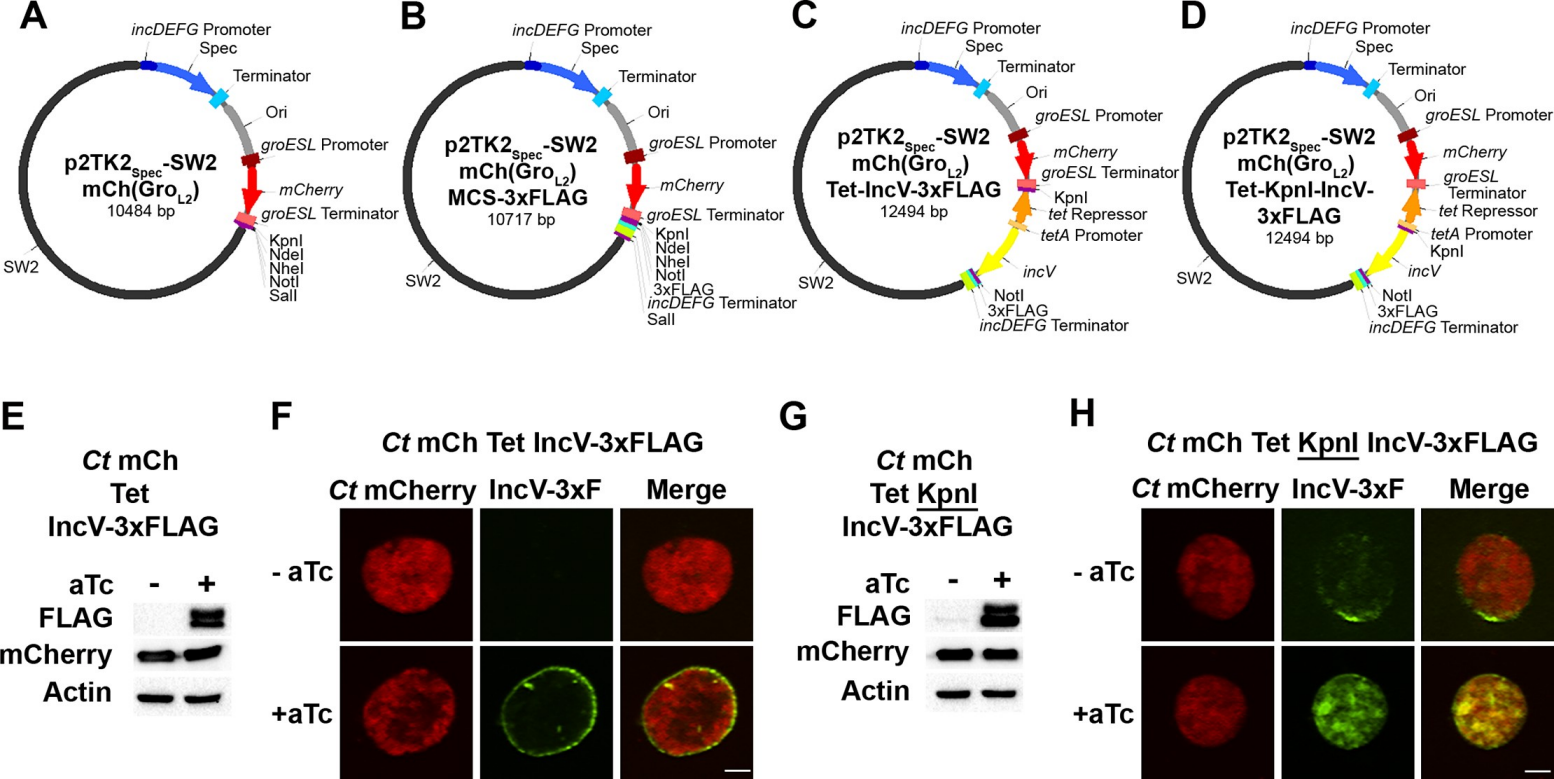
A new set of versatile spectinomycin resistant expression vectors for *C*. *trachomatis*. Vectors maps of p2TK2_Spec_-SW2 mCh(Gro_L2_) **(A)**, p2TK2_Spec_-SW2 mCh(Gro_L2_)-MCS-3xFLAG **(B)**, p2TK2_Spec_-SW2 mCh(Gro_L2_) Tet-IncV-3xFLAG **(C)**, and p2TK2_Spec_-SW2 mCh(Gro_L2_) Tet-KpnI-IncV-3xFLAG **(D)**. The pSW2 plasmid (SW2) is shown in black; the *incDEFG* operon promoter in dark blue; the *aadA* ORF that confers spectinomycin resistance (Spec) in blue; *aadA* terminator light blue; the *E*.*coli* origin of replication (Ori) in grey; the *groESL* operon promoter in dark red; the *mCherry* ORF in red; the *groESL* operon terminator in light red; unique restriction sites in violet; the *tet* repressor in orange; the *tetA* promoter in light orange; the *incV* ORF in yellow; the 3xFLAG tag in teal; the *incDEFG* operon terminator in light green. **(E, G)** Immuno-blots of cell lysates from HeLa cells infected with strains of *C*. *trachomatis* transformed with p2TK2_Spec_-SW2 mCh(Gro_L2_) Tet-IncV-3xFLAG **(E)** or p2TK2_Spec_-SW2 mCh(Gro_L2_) Tet-KpnI-IncV-3xFLAG **(G)** expressing mCherry constitutively and IncV-3xFLAG under the control of the aTc inducible promoter. The cells were infected for 24h in the absence (-aTc) or in the presence (+aTc) of 10ng/ml aTc added at 20h p.i. for 4h. Immuno-blots of the corresponding lysates were probed using antibodies against FLAG, mCherry, and actin. **(F, H)** Confocal micrographs of inclusions of the *C*. *trachomatis* strains as described in **E** and **G**, respectively. The cells were fixed 24h p.i., immunostained with anti-FLAG antibodies and imaged using a confocal microscope. A single plane crossing the middle of the inclusion is shown. The left panels correspond to the bacteria (*Ct* mCherry, red) and the middle panels to the 3xFLAG signal (IncV-3xF, green). The merge is shown on the right. Scale bar: 5μm.

p2TK2_Spec_-SW2 was further improved by the addition of a constitutively expressed mCherry fluorescent reporter driven by the *groESL* promoter, as previously described for our p2TK2_Amp_-SW2 derivatives [17]. The map and the sequence of the corresponding vector, which is referred to as p2TK2_Spec_-SW2 mCh(Gro_L2_), are respectively shown in Fig 1A and S1 Fig. We have also adapted p2TK2_Spec_-SW2 mCh(Gro_L2_), to include a 3xFLAG tag downstream from the NotI restriction site and upstream from the *incDEFG* terminator, to allow for convenient cloning and expression of translational fusion between *C*. *trachomatis* ORFs of interest and the 3xFLAG tag. The map and sequence of the corresponding vector, p2TK2_Spec_-SW2 mCh(Gro_L2_) MCS-3xFLAG, are presented in Fig 1B and S2 Fig, respectively.

We have also engineered the p2TK2_Spec_-SW2 mCh(Gro_L2_) vector to include the TetR repressor and the *tetA* promoter (tetA^P^) to express *C*. *trachomatis* ORFs from the anhydrotetracycline (aTc)-inducible promoter. A first vector, p2TK2_Spec_-SW2 mCh(Gro_L2_) Tet-IncV-3xFLAG was constructed by cloning a DNA fragment generated by overlapping PCR and comprising of TetR-tetA^P^-IncV into the KpnI and NotI restriction sites of p2TK2_Spec_-SW2 mCh(Gro_L2_) MCS-3xFLAG. The map and sequence of the resulting plasmid are presented in Fig 1C and S3 Fig, respectively. This configuration allows for a seamless junction between tetA^P^ and the start codon of IncV, which has been our preferred arrangement to avoid a potential disruption of aTc regulation [17, 27, 29, 51].

In an attempt to streamline the cloning process and avoid multiple rounds of overlap PCR, a second vector, p2TK2_Spec_-SW2 mCh(Gro_L2_) Tet-KpnI-IncV-3xFLAG was also generated (Fig 1D and S4 Fig for map and sequence respectively). With this configuration, the KpnI and NotI restriction sites present downstream of the TetR-tetA^P^ regulatory element and upstream of the 3xFLAG allow for a single step cloning of any gene of interest. The potential downside of this configuration is the introduction of a KpnI restriction site between tetA^P^ and the start codon of the downstream ORF, which could negatively affect the aTc dependent expression (see below).

Altogether, we present a new set of versatile, spectinomycin resistant expression vectors for *C*. *trachomatis*.

### Addition of nucleotides between tetA^P^ and the downstream ORF can affect aTc induction

We next tested if introducing a KpnI restriction site between *tetA*^*P*^ and the start codon of the downstream ORF would affect aTc induction. For this purpose, we used the T3SS effector IncV. We generated and introduced in *C*. *trachomatis* p2TK2_Spec_-SW2 mCh(Gro_L2_) Tet-IncV-3xFLAG and p2TK2_Spec_-SW2 mCh(Gro_L2_) Tet-KpnI-IncV-3xFLAG. The transformants were plaque purified and IncV expression was determined by immunoblot or immunofluorescence in the absence or presence of aTc. As previously observed with our Amp^R^ vector [29], the Tet-IncV-3xFLAG configuration led to the tight repression of IncV expression in the absence of aTc, with no IncV-3xFLAG protein detectable by immunoblot or immunofluorescence (Fig 1E left lane and 1F top panels). Addition of aTc, led to IncV-3xFLAG expression and localization to the inclusion membrane (Fig 1E right lane and 1F bottom panels). Similar levels of IncV-3xFLAG expression were obtained with aTc induction of the Tet-KpnI-IncV-NotI-3xFLAG construct (Fig 1G, right lane and 1H bottom panels). However, and although to a lesser extent, the IncV-3xFLAG protein was also expressed in the absence of aTc, as determined both by immunoblot and immunofluorescence (Fig 1G left lane and 1H top panels).

Altogether, these results suggest that introducing a KpnI restriction site between tetA^P^ and the start codon of IncV affected the aTc dependent repression of the promoter leading to weak but constitutive expression of the construct in the absence of inducer.

### A spectinomycin resistant cloning vectors for *Chlamydia muridarum*

**Fig 2:**
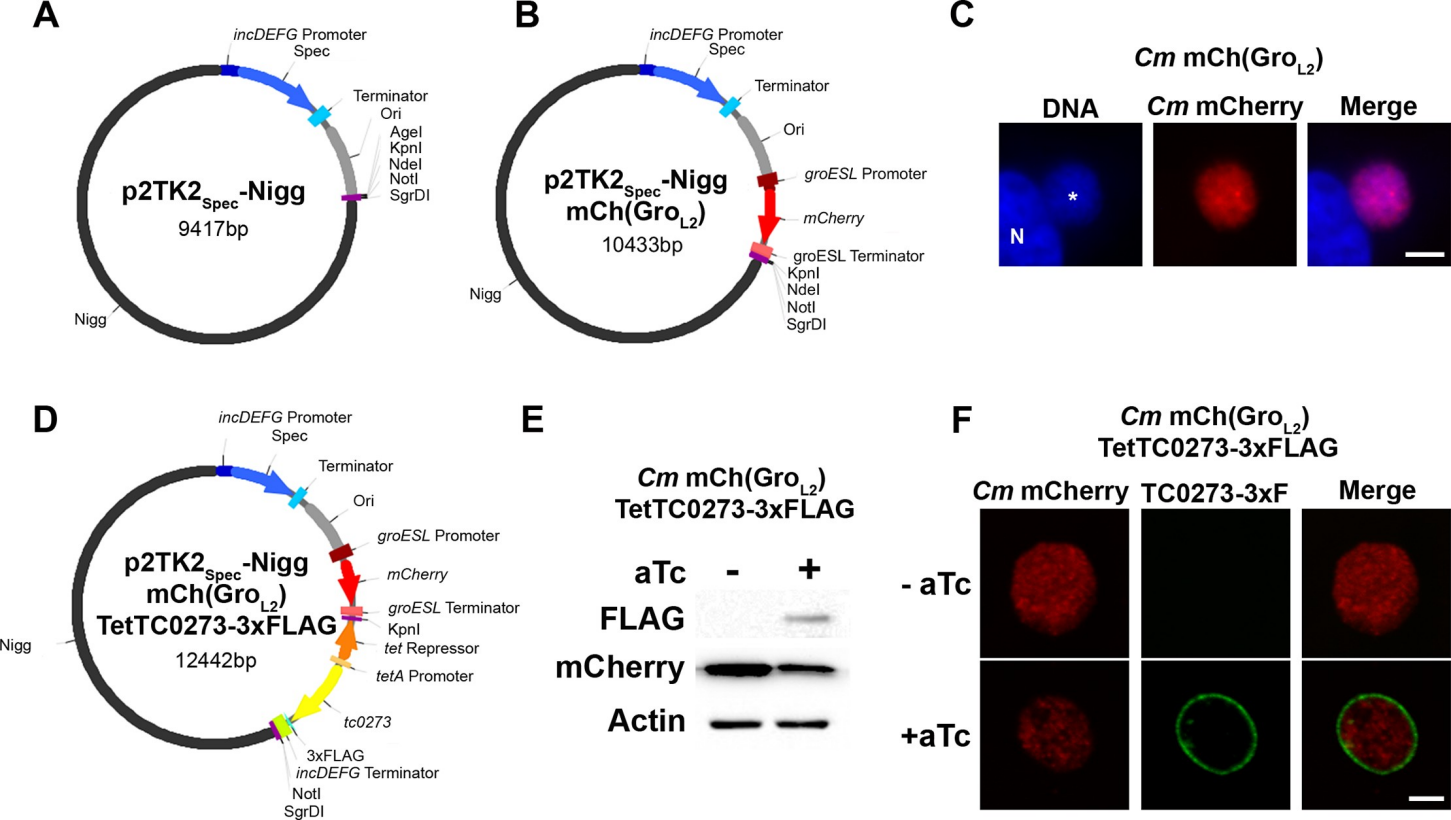
*C*. *muridarum* strain expressing mCherry from the *groESL* promoter and TC0273-3xFLAG under the control of the aTc inducible promoter. **(A-B)** Vector maps of p2TK2_Spec_-Nigg **(A)** and p2TK2_Spec_-Nigg mCh(Gro_L2_) **(B)**. The pNigg plasmid is shown in black; *incDEFG* promoter in dark blue; the *aadA* ORF that confers spectinomycin resistance (Spec) in blue; the *aadA* terminator light blue; the *E*. *coli* origin of replication (Ori) in grey; the *groESL* promoter in dark red; the *mCherry* ORF in red; the *groESL* terminator in light red; unique restriction sites in purple. **(C)** Immunofluorescence images of HeLa cells infected for 18h with a *C*. *muridarum* strain transformed with p2TK2_Spec_-Nigg mCh(Gro_L2_) stained with the Hoechst DNA dye. The left panel corresponds to the DNA (blue) with the nucleus labeled with “N” and the inclusion annotated with an asterisk. The middle panel corresponds to the bacteria (*Cm* mCherry, red), and the merge is shown on the right. Scale bar: 10μm. **(D)** Vector map of p2TK2_Spec_-Nigg mCh(Gro_L2_) TetTC0273-3xFLAG. The pNigg plasmid is shown in black; the *incDEFG* promoter in dark blue; the *aadA* ORF that confers spectinomycin resistance (Spec) in blue; the *aadA* terminator light blue; the *E*. *coli* origin of replication (Ori) in grey; the *groESL* promoter in dark red; *mCherry* ORF in red; the *groESL* terminator in light red; the Tet repressor in orange; the *tetA* promoter in light orange; the *tc0273* ORF in yellow; the 3xFLAG in teal; the *incDEFG* terminator in light green; unique restriction sites in purple. **(E)** Immuno-blot of cell lysates from HeLa cells infected with a strain of *C*. *muridarum* transformed with p2TK2_Spec_-Nigg mCh(Gro_L2_) TetTC0273-3xFLAG vector expressing mCherry constitutively and TC0273-3xFLAG under the control of the aTc inducible promoter. The cells were infected for 24h in the absence (-aTc) or presence (+aTc) of 10ng/ml aTc added at 2h p.i.. Immuno-blots of the corresponding lysates were probed using antibodies against FLAG, mCherry, and actin. **(F)** Confocal micrographs of inclusions of the *C*. *muridarum* strain as described in **E**. Cells were fixed 18h p.i., immunostained with anti-FLAG antibody and imaged using a confocal microscope. A single plane crossing the middle of the inclusion is shown. The left panels correspond to the bacteria (*Cm* mCherry, red), and the middle panels correspond to the 3xFLAG signal (TC0273-3xF, green). The merge is shown on the right. Scale bar: 5μm.

Skilton et al. have recently described, pGFP-Nigg, an *E*. *coli*-*C*. *muridarum* shuttle vector encoding CDS2-8 from the *C*. *muridarum* Nigg plasmid and CDS1 from the *C*. *trachomatis* SW2 plasmid, an ampicillin resistance cassette, and the green fluorescent protein (GFP) [39]. We have generated a different version of this plasmid, referred to as p2TK2_Spec_-Nigg, which includes a spectinomycin selectable marker (*aadA* under the *C*. *trachomatis incDEFG* promoter) and a MCS (see material and methods for details on cloning strategy) (Fig 2A and S5 Fig for map and sequence respectively). In addition, we have introduced the mCherry reporter under the control of the *C*. *trachomatis groESL* promoter to generate p2TK2_Spec_-Nigg mCh(Gro_L2_) (Fig 2B and S6 Fig for map and sequence respectively). The latter was transformed into *C*. *muridarum* following the same protocol used for *C*. *trachomatis* and led to mCherry expressing *C*. *muridarum* at the second passage (*Cm* mCherry). mCherry expression was stable after plaque purification of the strain (Fig 2C).

Altogether, these results indicate that spectinomycin can be used as a selectable marker for *C*. *muridarum* and that *C*. *trachomatis* promoters (i.e. *incDEFG* and *groESL* operon) and mCherry are expressed in *C*. *muridarum*.

### aTc inducible gene expression in *C*. *muridarum*

We next tested if the Tet system could be used for inducible gene expression in *C*. *muridarum*. For this purpose, the *tc0273* ORF, which encodes the *C*. *muridarum* IncV homolog, was cloned as a 3xFLAG translational fusion under the control of the *tetA* inducible promoter into the KpnI and NotI restriction sites of p2TK2_Spec_-Nigg mCh(Gro_L2_). Based on our results with *C*. *trachomatis* (Fig 1), we chose the configuration in which the *tc0273* ORF is seamlessly attached to *tetA*^P^ to ensure tight repression. The map and sequence of the resulting plasmid are presented in Fig 2D and S7 Fig, respectively. *C*. *muridarum* transformation resulted in mCherry expressing transformants that were plaque purified. In the absence of aTc, the resulting strain expressed mCherry only, as assayed by immunoblot and immunofluorescence (Fig 2E left lane and 2F top panels). In the presence of aTc, TC0273-3xFLAG expression was induced and detectable by immunoblot (Fig 2E right lane). TC0273-3xFLAG expression was confirmed by immunofluorescence microscopy, which also revealed that, as observed for its *C*. *trachomatis* homologue, TC0273 localized to the inclusion membrane (Fig 2F bottom panels).

Altogether, these results demonstrated that the Tet system can be used for inducible gene expression in *C*. *muridarum* and for studying the cellular localization of *C*. *muridarum* effectors, such as Inc proteins.

### A transcriptional fluorescent reporter to monitor RB to EB transition

**Fig 3:**
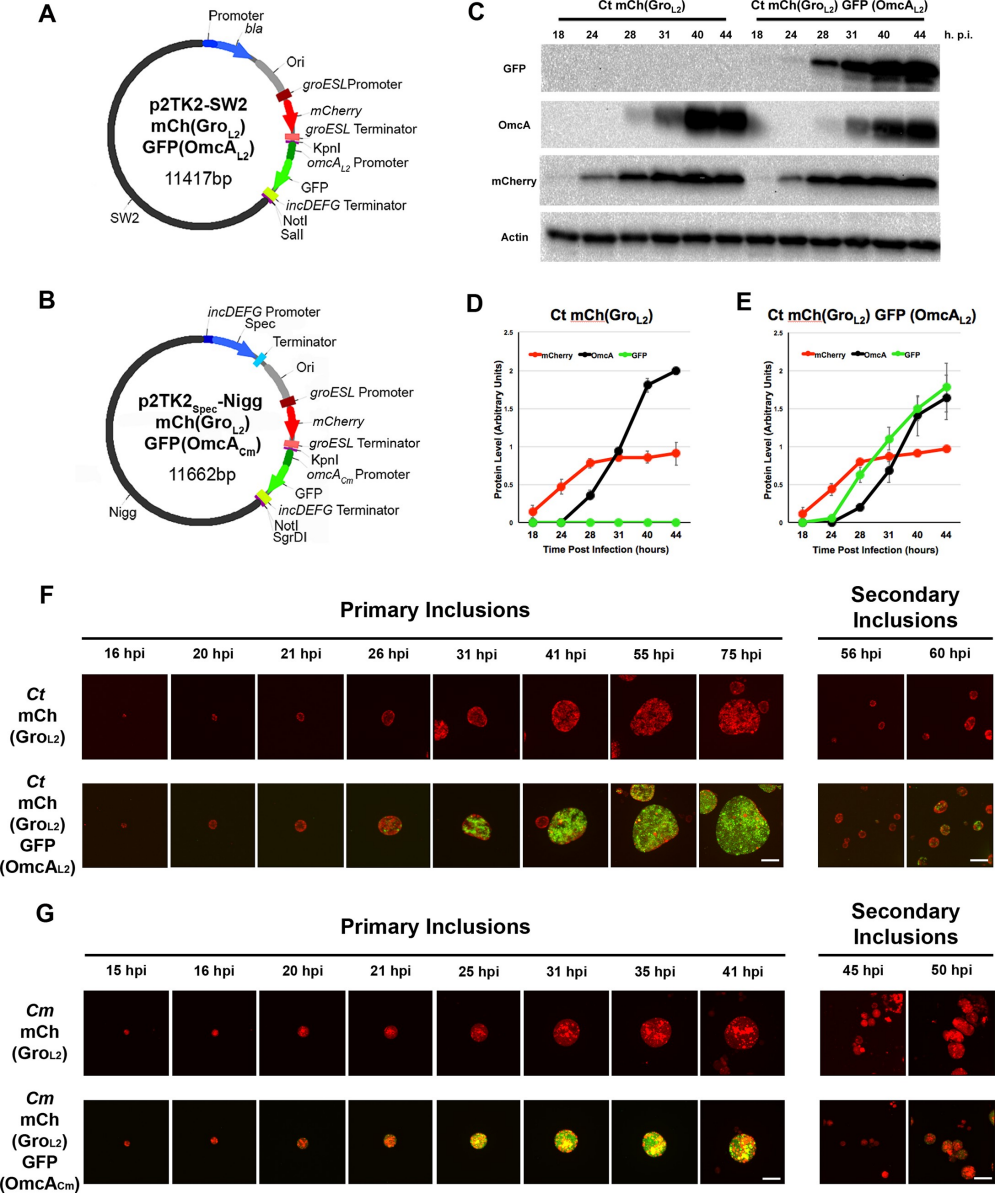
Transcriptional fluorescent reporters to monitor RB to EB transition in *C*. *trachomatis* and *C*. *muridarum*. Vector maps of p2TK2-SW2 mCh(Gro_L2_)GFP(OmcA_L2_) **(A)** and p2TK2-Nigg mCh(Gro_L2_)GFP(OmcA_Cm_) **(B)**. The pSW2 and pNigg plasmids are shown in black; the *incDEFG* promoter in dark blue; the spectinomycin resistance gene (*aadA*) and ampicillin resistance gene (*bla*) in blue; the terminator in light blue; the *E*. *coli* origin of replication (Ori) in grey; the *groESL* promoter in dark red; *mCherry* ORF in red; the *groESL* terminator in light red; the *omcA* promoter in dark green; GFP in green; the *incDEFG* terminator in light green; unique restriction sites in purple. **(C)** Immuno-blot of cell lysates from HeLa cells infected with a strain of *C*. *trachomatis* transformed with p2TK2_Spec_-SW2 mCh(Gro_L2_) or p2TK2_Spec_-SW2 mCh(Gro_L2_) GFP(OmcA_L2_). Lysates were collected at the indicated time and probed using antibodies against GFP, OmcA, mCherry, and actin. **(D-E)** Quantification of the signal intensity of the indicated marker from immunoblots as shown in **(C)**. The average signal intensity from three independent experiments is shown. mCherry: red, OmcA: black and GFP: green. **(F-G)** Selected 3-dimensional merged frames from Video S1 (Top panels, **(F)**), Video S2 (Bottom panels, **(F)**), Video S3 (Top panels, **(G)**) and Video S4 (Bottom panels, **(G)**) acquired by time-lapse video confocal microscopy of HeLa cells infected with *C*. *trachomatis* **(F)** or *C*. *muridarum* **(G)** expressing mCherry (red) from the *groESL* promoter (Top panels) or mCherry (red) and GFP (green) under the control of the *C*. *trachomatis groESL* and *omcA* promoter, respectively (Bottom panels). Frames were selected to provide representative examples of primary (left) and secondary (right) inclusions. The time p.i. is indicated above each frame. Scale Bar: 10μm.

Constitutive expression of a fluorescent reporter such as mCherry is a useful tool to monitor inclusion expansion, however it does not inform if and when the bacteria have undergone RB or EB conversion. For this purpose, we took advantage of the fact that a subset of *C*. *trachomatis* genes are expressed late in the developmental cycle, as the bacteria transition from RB to EB [40, 41]. One such gene is *omcA*. GFP was cloned under the control of the *C*. *trachomatis omcA* promoter and introduced between the KnpI and NotI restriction sites of p2TK2_Spec_-SW2 mCh(Gro_L2_). The resulting plasmid, p2TK2_Spec_-SW2 mCh(Gro_L2_) GFP(OmcA_L2_) (Fig 3A and S8 Fig for map and sequence, respectively) was transformed into *C*. *trachomatis* and the resulting strain, referred to as *Ct* mCh(Gro_L2_) GFP(OmcA_L2_), was plaque purified. Expression of mCherry or mCherry and GFP did not affect bacterial replication (not shown).

HeLa cells were infected with this strain or with *C*. *trachomatis* expressing mCherry only (*Ct* mCh(Gro_L2_)). At various times from 18h p.i., when the inclusions mostly harbor RBs, to 44h p.i., when *C*. *trachomatis* completes its developmental cycle and most of the bacteria have converted to EB, samples were collected and analyzed by immunoblot using antibodies against GFP, OmcA, mCherry and actin (Fig 3C). Quantification of three independent experimental replicates are presented in Fig 3D and 3E. As expected for a constitutively expressed reporter, both strains were positive for mCherry at all time points analyzed (Fig 3C mCherry and 3D and 3E red line). Also as expected, the overall intensity of the mCherry signal increased over time, especially during the first 30h, reflecting RB multiplication. For both strains, a weak OmcA signal was detectable starting at 28h p.i., when asynchronous RB to EB conversion is occurring. The OmcA signal intensified over time as the developmental cycle progressed and more RBs transitioned into EBs (Fig 3C OmcA and 3D and 3E black line). Additionally, GFP was only detected in the *Ct* mCh(Gro_L2_) GFP(*omcA*_L2_) strain, and most importantly for the validation of our approach, the expression of GFP mirrored the expression of endogenous OmcA (Fig 3C GFP and 3D and 3E green line).

Altogether these results indicate that, at the population level, GFP expression from the *C*. *trachomatis omcA* promoter correlates with the expression of endogenous OmcA and therefore constitute an adequate reporter of the RB to EB conversion during *C*. *trachomatis* developmental cycle.

### Visualizing *C*. *trachomatis* RB to EB transition by time lapse-microscopy

We next performed time-lapse confocal microscopy to visualize the expression of the mCherry and GFP reporters at a single inclusion level. For this purpose, HeLa cells infected with the *Ct* mCh(Gro_L2_) or *Ct* mCh(Gro_L2_) GFP(OmcA_L2_) strain were imaged starting 8h p.i.. Z stacks were acquired every 20min for 70h to capture several rounds of infections. Selected frames and the corresponding movies are presented in Fig 3F and S1 and S2 Movies, respectively. With the *Ct* mCh(Gro_L2_) strain, mCherry expression was detected early when the inclusion harbored only a few bacteria (Fig 3F, top panels, Primary inclusions, 16h). Moreover, the bacteria remained fluorescent during the entire duration of the movie, which included the formation of primary and secondary inclusions (Fig 3F, top panels, Secondary inclusions, 56h). The *Ct* mCh(Gro_L2_) GFP(*omcA*_L2_) strain presented similar timing of mCherry expression to the *Ct* mCh(Gro_L2_) strain (Fig 3F, bottom panels, Primary inclusions). In addition, in primary inclusions, a few bacteria started to express GFP at 26h p.i. (Fig 3F, bottom panels, Primary inclusions, 26h). The number of GFP-positive bacteria per inclusion progressively increased over time, until the inclusion was eventually filled with GFP-expressing bacteria (Fig 3F, bottom panels, Primary inclusions). Importantly, the same pattern of mCherry and GFP expression was observed during secondary infections. Early on, secondary inclusions were mCherry-positive only and displayed increasing GFP expression as time progressed (Fig 3F, bottom panels, Secondary inclusions, compare 56h and 60h), further confirming the temporal regulation of the GFP reporter.

Altogether, these results indicate that our fluorescent reporters allow for live imaging of early to late events of *C*. *trachomatis* developmental cycle. Moreover, we showed that, in *C*. *trachomatis*, the *omcA* promoter-dependent expression of GFP leads to a temporal expression, which correlates with the late developmental stages of the bacteria, therefore constituting an adequate reporter for monitoring *C*. *trachomatis* RB to EB conversion.

### Visualizing *C*. *muridarum* developmental cycle, including RB to EB transition, by time lapse-microscopy

We next investigated if the RB to EB transition *omcA* reporter could be used in *C*. *muridarum*. For this purpose, GFP was cloned under the control of the *C*. *muridarum omcA* promoter and introduced between the KpnI and NotI restriction sites of p2TK2_Spec_-Nigg mCh(Gro_L2_). The resulting plasmid, p2TK2_Spec_-Nigg mCh(Gro_L2_) GFP(OmcA_Cm_) (Fig 3B and S9 Fig for map and sequence, respectively) was transformed into *C*. *muridarum* and the derived strain, *Cm* mCh(Gro_L2_) GFP(OmcA_Cm_) was plaque purified. HeLa cells infected with *Cm* mCh(Gro_L2_) or *Cm* mCh(Gro_L2_) GFP(OmcA_Cm_) were analyzed by time-lapse confocal microscopy under the same conditions described above for *C*. *trachomatis*. Selected frames and the corresponding movies are presented in Fig 3G and S3 and S4 Movies, respectively. Live imaging of the *Cm* mCh(Gro_L2_) strain revealed that the mCherry reporter was expressed early and throughout the duration of the movie which included secondary infections (Fig 3G Top panels). In addition, comparison of the kinetics of the developmental cycle of the mCherry expressing *C*. *trachomatis* and *C*. *muridarum* strains, indicated that *C*. *muridarum* developmental cycle appeared to progress faster than *C*. *trachomatis* developmental cycle (Compare time points of the selected frames in Fig 3F and 3G). Live imaging of the *Cm* mCh(Gro_L2_) GFP(OmcA_Cm_) strain revealed that, early on, inclusions were expressing mCherry only, but progressive and increasing appearance of the GFP signal occurred as the developmental cycle progressed (Fig 3G Bottom panels, Primary inclusions). Moreover, nascent secondary inclusions were at first mCherry-positive but GFP-negative (Fig 3G bottom panels, Secondary inclusions, compare 45h and 50h).

Altogether, these results indicated that our mCherry reporter allows for live imaging of early and late events of *C*. *muridarum* developmental cycle. They also revealed that in our experimental set up *C*. *muridarum* appeared to go through one round of infection at a faster pace than *C*. *trachomatis*. Moreover, our data indicated that GFP expression from the *C*. *muridarum omcA* promoter was temporally regulated and coincided with late stages of the developmental cycle, supporting the use of this reporter for monitoring RB to EB conversion.

### Spatiotemporal analysis of RB to EB transition in *C*. *trachomatis*

**Fig 4:**
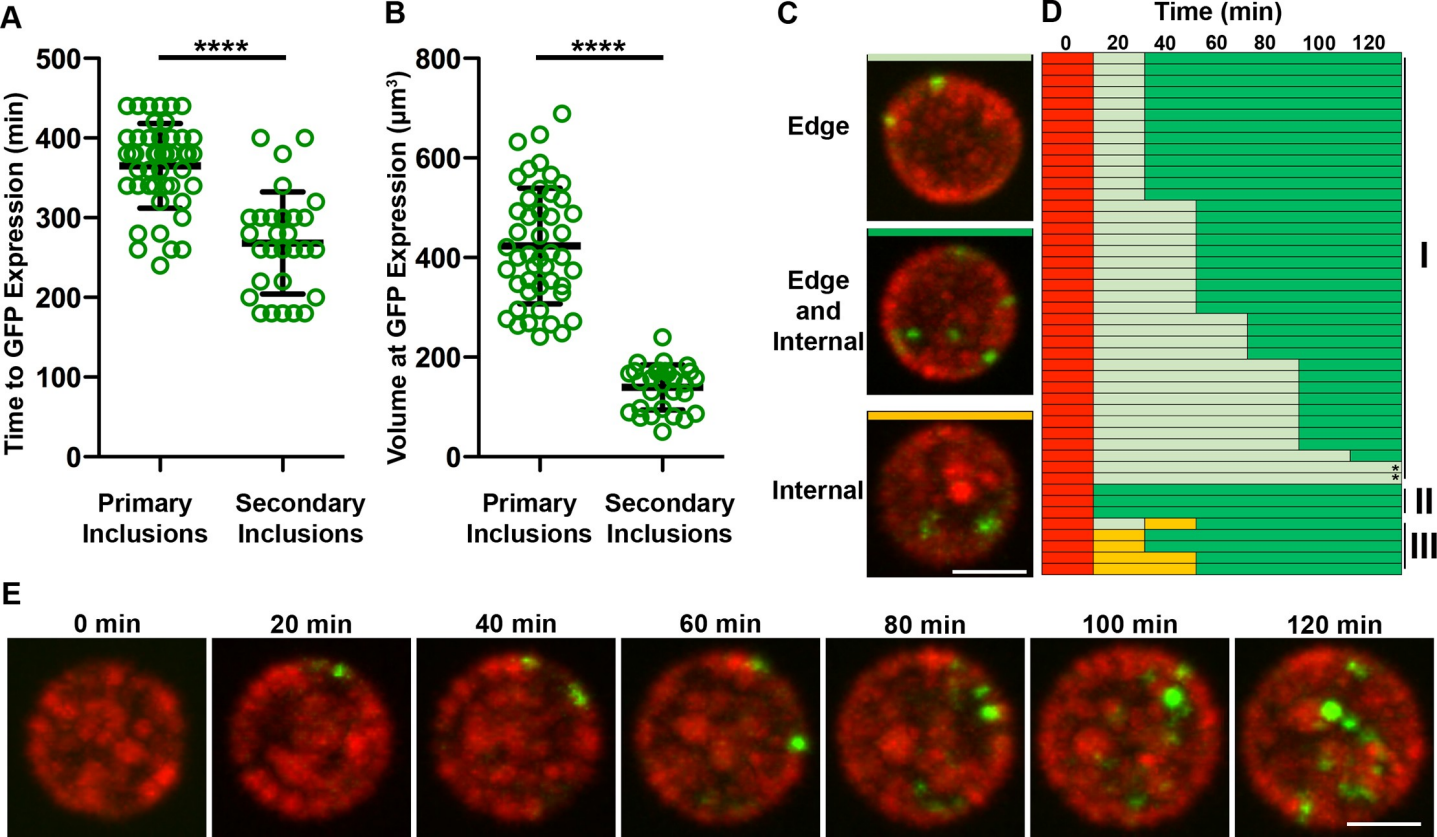
Spatiotemporal analysis of RB to EB transition in *C*. *trachomatis*. **(A-B)** Quantification of time from 4–6 RBs per inclusion to first GFP expression in minutes **(A)** and of the volume of the inclusions at the time of first GFP expression in μm^3^ **(B)**. Measurements were performed on primary and secondary inclusions harboring the *C*. *trachomatis* mCh(Gro_L2_) GFP(OmcA_L2_) strain and imaged by time-lapse video confocal microscopy. Each circle represents one inclusion. Error bars are Standard Deviation. ****P < 0.0001. **(C)** Representative 3-dimensional merged frames acquired by time-lapse video confocal microscopy of *C*. *trachomatis* mCh(Gro_L2_) GFP(OmcA_CL2_) inclusions exhibiting GFP-positive bacteria at the edge only (top panel, light green), both edge and internal (middle panel, dark green), and internal only (bottom panel, orange). Red: mCherry, Green: GFP. Scale Bar: 5 μm. **(D)** Tracking of the spatiotemporal distribution of the GFP signal over time in primary inclusions using categories defined in (C). Time prior to GFP expression is represented in red. Inclusions were grouped in three classes (I, II, III) based on spatiotemporal pattern of GFP expression. **(E)** Selected 3-dimensional merged frames acquired by time-lapse video confocal microscopy and representative of Class I inclusions, where GFP localization change from edge only to edge and internal over time. Red: mCherry, Green: GFP. Scale Bar: 5μm.

To gain insight into the spatiotemporal dynamics of the RB to EB transition, we analyzed when and where the GFP signal was first observed. Time-lapse confocal microscopy was performed on HeLa cells infected with the *Ct* mCh(Gro_L2_) GFP(OmcA_L2_) strain, as described in Fig 3, and individual inclusions were tracked over time. We first quantified the time required for inclusions containing 4 to 6 mCherry-positive bacteria, a stage at which we could confidently identify inclusions, to display the first GFP-positive bacteria (Fig 4A). The fastest and slowest primary inclusions displayed GFP-positive bacteria after 240min and 440min, respectively. Overall, the average time for the initiation of RB to EB conversion in primary inclusion was 365min +/-53min, once the inclusion contains 4–6 bacteria. This number was significantly reduced in secondary inclusions (268min +/-64min). We also determined that, when the first GFP-positive bacteria were observed, the average volume of the inclusions was 423μm^3^ +/-116 μm^3^ and 139 μm^3^ +/-45 μm^3^ in primary and secondary inclusions, respectively (Fig 4B).

We next determined if the GFP signal was occurring randomly or at a specific location within the inclusion. For this purpose, primary inclusions were monitored until GFP expression was first detected and the spatial distribution of the GFP signal was tracked and recorded over time. Three categories of inclusions were observed. The GFP signal was at the edge only, at the edge and internally, or internally only (Fig 4C, light green, dark green and orange respectively). In all cases, at the time of conversion, the GFP signal was restricted to one to three bacteria. Tracking of multiple primary inclusions revealed that for 85% of the inclusions, an example of which is provided in Fig 4E, the GFP signal appeared first in one to three bacteria localized at the edge of the inclusion only (Fig 4C, top panel, light green and 4D, Class I, light green). As time progressed, the GFP signal was observed in bacteria localizing at the edge of the inclusion but also in the center of the inclusion (Fig 4C, middle panel, dark green and 4D, Class I, dark green). The time to transition from inclusions containing GFP-positive bacteria at the edge only, to inclusions with GFP-positive bacteria at the edge and internally, ranged from 20min to 120min (Fig 4D, Class I). Two inclusions, annotated with an asterisk, required longer (240min and 160min). For about 6% of the primary inclusions, the GFP signal was observed at the edge and internally at all time points analyzed (Fig 4C, middle panel, dark green and 4D, Class II, dark green). Because frames were acquired every 20min, it is possible that we were not able to capture these inclusions with GFP-positive bacterium at the edge only, despite its occurrence. If so, it would suggest that transition from edge only to edge and internal could occur in less that 20min. Finally, 9% of the primary inclusions displayed the GFP signal internally at first and at the edge and internally later on (Fig 4C, bottom panel, orange and 4D, Class III). It is also possible that the initial GFP expressing bacteria at the edge of the inclusion was missed because of the timing of the acquisition. Secondary inclusions followed the same trend (not shown).

Altogether our spatiotemporal analysis of RB to EB conversion suggests that this process starts at a specific time (365min +/-53min and 268min +/-64min in primary and secondary inclusions, respectively), after the bacteria have undergone 2–3 rounds of replication, and correlates with a specific size of the inclusion (423μm^3^ +/- 116μm^3^ and 139μm^3^ +/- 45μm^3^ in primary and secondary inclusions, respectively). In addition, we provide evidence that the first bacteria to convert localize at the edge of the inclusion and that within 20-120min converting/converted bacteria are observed at the edge and in the lumen of the inclusion.

## Discussion

### Spectinomycin resistant vectors for *Chlamydia*

Beta-lactams, such as ampicillin or penicillin, were the first antibiotics used for the selection of *C*. *trachomatis* transformants [14]. Chloramphenicol and spectinomycin selections were described later on [21, 49]. However, most of the *C*. *trachomatis* vectors available so far display the beta-lactamase gene and therefore cannot be used for the complementation of TargeTron or FRAEM mutants in which the beta-lactamase gene either interrupts or replaces the targeted gene [22, 52]. To circumvent this problem and complement a *ct383*::*bla* mutant, Weber et al. inserted the *aadA* gene under the control of the *incD* promoter in their pBOMB4-Tet-CT383 vector, leading to a vector that conferred resistance to spectinomycin, in addition to ampicillin [20]. Our p2TK2-SW2 derivatives offer the advantage of conferring resistance to either ampicillin (p2TK2_Amp_-SW2) or spectinomycin (p2TK2_Spec_-SW2). Moreover, since their MCS are compatible, transgenes can easily be transferred from one vector to another.

### Cloning downstream of the *tet*^*A*^ promoter

We have cloned the *C*. *trachomatis incV* ORF downstream of the *tet*^*A*^ promoter, either seamlessly, or by introducing a KpnI restriction site between the promoter and the start codon. Seamless junction led to a tight aTc regulation with the construct being off or on in the absence or presence of aTc, respectively. In addition, when expressed, IncV localized to the inclusion membrane. This is in contrast with the construct that included a KpnI site. This configuration led to leaky expression of IncV in the absence of inducer. In addition, a great proportion of the IncV molecules appeared to be trapped inside the bacteria. The only difference between the 2 constructs is the presence of the KpnI restriction, which would suggest that the addition of the corresponding 6 nucleotides is responsible for affecting tight repression, but also translocation. Potential reasons for the latter are unclear to us. Wickstrum et al. included an AgeI site between the *tet*^*A*^ promoter and start codon of GFP without affecting regulation [16]. Similarly, Bauler et al. used a NotI site [18]. At this point we cannot conclude if our results are specific to IncV and/or KpnI; regardless, they indicate that caution should be taken when designing strategies to clone effectors downstream of the *tet*^*A*^ promoter.

### A versatile vector for *Chlamydia muridarum*

Since the development of genetic tools to study *Chlamydia*, there has been a bias towards *C*. *trachomatis*. Tools for *C*. *muridarum*, the mouse adapted strain commonly used to model human genital tract ascending infections in mice, are still limited. Most of the vectors described so far have been used to investigate the role of plasmid encoded ORFs in *C*. *muridarum* virulence [34, 35, 37–39]. Here, we present *C*. *muridarum* vectors that are as versatile as the one described for *C*. *trachomatis*. We have shown that in addition to GFP [39] and luciferase [37], mCherry can be expressed in *C*. *muridarum* without affecting its developmental cycle. Our study also reveals that spectinomycin can be used to select for transformants, hereby providing an additional selection marker for *C*. *muridarum*. By including a MCS, to facilitate the cloning of any transgene of interest, and an aTc inducible promoter, we have performed a tagged effector localization study. Our results indicate that the Tet inducible system is functional in *C*. *muridarum* and confirmed the predicted inclusion localization of TC0273 [11]. Altogether, increasing the versatility of *C*. *muridarum* vectors will be instrumental for mutant complementation and subsequent *in vivo* studies.

We were able to image *C*. *muridarum* developmental cycle using a constitutive mCherry reporter or a reporter for RB to EB conversion. Our live imaging analysis indicates that constitutive mCherry expression allows for detection of nascent inclusions containing a few RBs. In addition, we noticed that *C*. *muridarum* secondary inclusions are detected earlier than *C*. *trachomati*s secondary inclusions and that the developmental cycle progresses almost twice as fast as *C*. *trachomati*s developmental cycle, in agreement with a previous report [53].

### Advantages of a fluorescent reporter to monitor RB to EB conversion

Inclusion Forming Units (IFUs) assay, where the number of infectious bacteria (i.e. EBs) recovered at a given stage of the developmental cycle is determined by infection of a fresh monolayer of cells with serial dilutions of infected cells lysates, is a standard assay to determine if *Chlamydia* undergoes a productive developmental cycle. While accurately measuring the amount of EBs, this assay is labor intensive, limited in the number of conditions that can be tested at once, and not easily adaptable to high-throughput screening. Moreover, it does not provide information on potential defects in entry, EB to RB conversion or RB replication.

Electron microscopy (EM) has been instrumental in investigating RB to EB conversion due to the clear morphological difference of the two developmental forms [5–7]. Although EM offers the unambiguous identification of the different *Chlamydia* forms, it is labor intensive and the number of inclusions that can be analyzed is limited. In addition, 3D EM revealed that the distribution of EB and RB within the inclusion is heterogeneous, therefore introducing a bias when analyzing inclusion content by conventional 2D EM [7].

*Chlamydia* intracellular development can also be evaluated by using a fluorescence-based assay where the surface area of the inclusions, as determined per immunostaining of the bacteria or using fluorescent strains, is quantified using computer assisted software. This assay allows for the analysis of a large number of inclusions and is amenable to high-throughput formats. However, it does not provide a full picture of the developmental cycle. Some inclusions could be large and yet mostly contain RBs. On the contrary, small inclusions could be filled with EBs, as shown with Brefeldin A treatment [54].

Here, we have designed a fluorescent reporter to monitor RB to EB conversion. Our rationale stemmed from earlier studies that demonstrated temporal expression of specific *Chlamydia* genes throughout the developmental cycle [40, 41]. Of particular interest to our design were genes, like *omcA*, whose transcription initiation corresponded to the time of RB to EB conversion. As anticipated, the timing and asynchronous nature of the expression of the transcriptional *omcA*::*gfp* reporter closely mimics the expression of endogenous OmcA. In addition, the timing of GFP expression, as a readout for RB to EB conversion, was consistent with results from previous EM studies. For example, in their 3D EM analysis of the developmental cycle, Lee et al. observed the first intermediate bodies at 24h p.i. [7], which is the same time point at which we observed the first GFP-expressing *C*. *trachomatis*. Moreover, Lee et al. also reported that by 40h p.i., 70% of the bacteria were EBs, which is also in agreement with our *C*. *trachomatis* imaging results where the inclusions were filled with EBs at that time point.

Altogether, our RB to EB conversion reporter offers an alternative that combines the advantages of the assays listed above, while alleviating some of the limitations. Fluorescence microscopy analysis of cells infected with *Chlamydia* strains encoding the fluorescent RB to EB conversion reporter allows for quantification, in a single assay, of: 1) the size of the inclusion based on the mCherry signal, 2) the proportion of GFP-positive inclusions in which RB have converted to EB, 3) the proportion of RB that have converted to EB in a given inclusion (i.e. GFP-positive bacteria). Moreover, live imaging can be used to measure the timing of these events. These readouts would be applicable to genome wide siRNA or CRISPR screens or screening of small molecules that inhibit *Chlamydia* developmental cycle. The reporter could also be introduced in *Chlamydia* mutants to monitor potential growth defects. Finally, one could also envision that this new tool could be instrumental in visualizing the RB to EB transition *in vivo*.

### Spatiotemporal analysis of RB to EB transition in *C*. *trachomatis*

By tracking individual inclusions harboring our *C*. *trachomatis* RB to EB reporter strain, we were able to gather spatiotemporal information of RB to EB conversion. In primary inclusions, the timing of the initiation of RB to EB conversion was fairly homogenous and averaged to 365min +/-53min, after the bacteria had undergone 2–3 rounds of replication. It also correlated with similar inclusion volumes (423μm^3^ +/-116μm^3^). In secondary inclusions, although the average time to conversion was also consistent from one inclusion to another, it was significantly faster than in primary inclusions (268min +/-64min) and correlated with smaller inclusions (139μm^3^ +/-45μm^3^). In our experimental set up, the environment in which primary and secondary inclusions matured was different. Primary inclusions developed in an undisturbed environment, whereas secondary inclusions developed in the presence of lysed infected cells and high numbers of released EB that continue to infect the monolayer. In addition, it is possible that specific nutrients are being depleted, resulting in some level of starvation of the bacteria in secondary inclusions. Altogether our results indicate that the initiation of RB to EB conversion occurs at a specific time after the RBs start to replicate and once the inclusion has reached a certain size. However, the timing and inclusion size appear to vary depending on the environment, suggesting that external signals may also influence this process.

We were also able to visualize where RB to EB conversion is initiated. Our data indicate that the first one to three bacteria to undergo conversion are localized at the edge of the inclusion. Within 20–120 min of the first conversion event, a different set of GFP-expressing bacteria localized at the edge and some GFP-positive bacteria were present in the lumen of the inclusion. These results could fit a model by which RB to EB transition is initiated in bacteria that are in close proximity to the inclusion membrane and is followed by the subsequent accumulation of converting/converted bacteria in the center of the inclusion. This model would follow the one proposed by Wilson et al. in which replicating RBs are attached to the inclusion though their type III secretion system and over time detachment of RBs from the inclusion membrane provides the signal for differentiation into EB which accumulate in the lumen of the inclusion [47]. Because our experimental setup does not allow us to determine if converting RBs are physically attached to the inclusion membrane, we cannot confirm at this time if RB detachment is required to initiate conversion or if completion of the conversion requires detachment. It is also unclear if the size of the RBs controls this process [7]. Regardless, our data clearly indicate that the first event of RB to EB conversion occurs in RB localized at the periphery of the inclusion.

## Conclusion

Altogether, we present here a new set of genetic tools for *C*. *trachomatis* and *C*. *muridarum* that will be instrumental as the field continues to harness the power of genetics to study this important pathogen.

## Supporting information

S1 Table Primers used in this study. (DOCX)

S1 Fig p2TK2_Spec_-SW2 mCh(Gro_L2_) sequence. (DOCX)

S2 Fig p2TK2_Spec_-SW2 mCh(Gro_L2_) MCS-3xFLAG sequence. (DOCX)

S3 Fig p2TK2_Spec_-SW2 mCh(Gro_L2_) Tet-IncV-3xFLAG sequence. (DOCX)

S4 Fig p2TK2_Spec_-SW2 mCh(Gro_L2_) Tet-KpnI-IncV-NotI-3xFLAG sequence. (DOCX)

S5 Fig p2TK2_Spec_-Nigg sequence. (DOCX)

S6 Fig p2TK2_Spec_-Nigg mCh(Gro_L2_) sequence. (DOCX)

S7 Fig p2TK2_Spec_-Nigg mCh(Gro_L2_) TetTC0273-3xFLAG sequence. (DOCX)

S8 Fig p2TK2-SW2 mCh(Gro_L2_) GFP(OmcA_L2_) sequence. (DOCX)

S9 Fig p2TK2_Spec_-Nigg mCh(Gro_L2_) GFP(OmcA_Cm_) sequence. (DOCX)

S1 Movie Time-lapse video microscopy of the developmental cycle of mCherry expressing *C. trachomatis*. Video S1 shows the development of primary and secondary inclusions of *C*. *trachomatis* expressing mCherry under the control of the *C*. *trachomatis groESL* promoter. Infected HeLa cells were monitored starting 8h p.i. by spinning disc confocal microscopy. A z-stack of 40 images covering 40**μ**m was acquired every 20min over the course of 70h. The mp4 video was generated using Imaris imaging software and the HandBrake compression platform. The video is played at 10 frames/s. The time (hours:min:seconds) is indicated in the upper right corner. Scale Bar: 20**μ**m.
(MP4)

S2 Movie Time-lapse video microscopy of the *C. trachomatis* developmental cycle using a transcriptional fluorescent reporter to monitor RB to EB transition. Video S2 shows the development of primary and secondary inclusions and the RB to EB transition that occurs during *C*. *trachomatis* infection. HeLa cells were infected with a *C*. *trachomatis* strain that expresses mCherry under the control of the *C*. *trachomatis groESL* promoter and GFP under the control of the *C*. *trachomatis omcA* promoter. 8h p.i., infected cells were monitored by spinning disc confocal microscopy. A z-stack of 40 images covering 40**μ**m was acquired every 20min over the course of 70h. The mp4 video was generated using Imaris imaging software and the HandBrake compression platform. The video is played at 10 frames/s. The time (hours:min:seconds) is indicated in the upper right corner. Scale Bar: 20**μ**m.
(MP4)

S3 Movie Time-lapse video microscopy of the developmental cycle of mCherry expressing *C. muridarum*. Video S3 shows the development of primary and secondary inclusions of *C*. *muridarum* expressing mCherry under the control of the *C*. *trachomatis groESL* promoter. Infected HeLa cells were monitored starting 8h p.i. by spinning disc confocal microscopy. A z-stack of 40 images covering 40**μ**m was acquired every 20min over the course of 70h. The mp4 video was generated using Imaris imaging software and the HandBrake compression platform. The video is played at 10 frames/s. The time (hours:min:seconds) is indicated in the upper right corner. Scale Bar: 20**μ**m.
(MP4)

S4 Movie Time-lapse video microscopy of the *C. muridarum* developmental cycle using a transcriptional fluorescent reporter to monitor RB to EB transition. Video S4 shows the development of primary and secondary inclusions and the RB to EB transition that occurs during *C*. *muridarum* infection. HeLa cells were infected with a *C*. *muridarum* strain that expressed mCherry under the control of the *C*. *trachomatis groESL* promoter and GFP under the control of the *C*. *muridarum omcA* promoter. 8h p.i., infected cells were monitored by spinning disc confocal microscopy. A z-stack of 40 images covering 40**μ**m was acquired every 20min over the course of 70h. The mp4 video was generated using Imaris imaging software and the HandBrake compression platform. The video is played at 10 frames/s. The time (hours:min:seconds) is indicated in the upper right corner. Scale Bar: 20**μ**m.
(MP4)
