# Supplementary material for: Chlamydia trachomatis and Chlamydia muridarum spectinomycin resistant vectors and a transcriptional fluorescent reporter to monitor conversion from replicative to infectious bacteria
Source: PLoS One. 2019 Jun 6;14(6):e0217753. doi: 10.1371/journal.pone.0217753 (PMC6553856; doi:10.1371/journal.pone.0217753)
Supplement: S1 Table — (DOCX) [file pone.0217753.s001.docx]

**Table S1: Primers used in this study.**

| **Primer Name** | **Primer Sequence** |
| --- | --- |
| MCSIncDPromFw | GCGGCCGCGTCGACGGATCCAACGGAGCCTTCTAGCTATTTTG |
| aadA-TermOrigRv | GAGTAAACTTGGTCTGACAGTAGTTACAAATTGTTTCACT |
| IncDPromNot5 | GCGGGCGGCCGCAACGGAGCCTTCTAGCTATTTTG |
| IncDPromaad3 | GACCAGTTGCGTGAGCGCATAAACCTCACTTCGACAGATTTTAGCC |
| IncDPromaad5 | GACCAGTTGCGTGAGCGCATAAACCTCACTTCGACAGATTTTAGCC |
| aadTerm3 | GTTATTGCAATAAAATTAGCTTATTTGCCGACTACCTTGG |
| aadTerm5 | CCAAGGTAGTCGGCAAATAAGCTAATTTTATTGCAATAAC |
| TermNot3 | GCGGGCGGCCGCTAGTTACAAATTGTTTCAC |
| aadA-TermOrigFw | AGTGAAACAATTTGTAACTACTGTCAGACCAAGTTTACTC |
| MCSIncDPromRv | CAAAATAGCTAGAAGGCTCCGTTGGATCCGTCGACGCGGCCGC |
| NotI3xFLAGFw | GGCGGCCGCATGGACTACAAAGACCATGACG |
| IncDTermRv | GTCGTCGACGTCTTAGGAGCTTTTTGCAATGC |
| TetRSTOP5Kpn | GGTGGTACCTTAAGACCCACTTTCACATTTAAG |
| IncVNotIRv | GCGGCGGCCGCCTTTACGAGAGGGTTTCTTCTTTTG |
| AgeITetRStopFw | ACCACCGGTTTAAGACCCACTTTCACATTTAAG |
| TetAPKpnIRv | GGTGGTACCTTCACTTTTCTCTATCACTG |
| KpnIIncVFw | GGTGGTACCATGACTCCAGTAACACCAGTCC |
| TetTC02733 | GTTGGCGTTATTGGGGCCATTTCACTTTTCTCTATCACTG |
| TetTC02735 | CAGTGATAGAGAAAAGTGAAATGGCCCCAATAACGCCAAC |
| 0273FLAG3 | CATGGTCTTTGTAGTCCATTTTAGGTCGAGACCTGTATTTTTTCC |
| 0273FLAG5 | GGAAAAAATACAGGTCTCGACCTAAAATGGACTACAAAGACCATG |
| IncDTerm3Not | GCGGGCGGCCGCGTCTTAGGAGCTTTTTGCAATGC |
| OmcAProm5Kpn | GGTGGTACCCATCCCTCCGATTCTTGTATTA |
| OmcARSGFP3 | GTGCTTCTCCTTTACTCATAACTTCCAGACTCCTTTCTAG |
| OmcARSGF5 | CTAGAAAGGAGTCTGGAAGTTATGAGTAAAGGAGAAGCAC |
| CmOmcAprom3KpnI | GGTGGTACCTTTTTTACCCCTTTGATTC |
| CmOmcApromRSGFP5 | CTAGAAAGGAGTCTGGAAGTTATGAGTAAAGGAGAAGCAC |
| CmOmcApromRSGFP3 | GTGCTTCTCCTTTACTCATAACTTCCAGACTCCTTTCTAG |

| **Sequencing Primers** |  |
| --- | --- |
| GroESLmChSTOP5 | GGACGAGCTGTACAAGTAGTTCCTCTAATGGGAACAAATAG |
| 2TK2SW2SpecRev | GTGGGTGTTTGTACTAGAGG |
| Nigg-Rev | ATATCACAATATTGTGGGTG |
