# Supplementary material for: Chlamydia trachomatis and Chlamydia muridarum spectinomycin resistant vectors and a transcriptional fluorescent reporter to monitor conversion from replicative to infectious bacteria
Source: PLoS One. 2019 Jun 6;14(6):e0217753. doi: 10.1371/journal.pone.0217753 (PMC6553856; doi:10.1371/journal.pone.0217753)
Supplement: S2 Fig — (DOCX) [file pone.0217753.s003.docx]

**Plasmid p2TK2_Spec_-SW2 mCh(Gro_L2_) MCS-3xFLAG features**

*incDEFG* Promoter: bases 1-228

*aadA* ORF (spectinomycin resistance): bases 229-1239

Terminator: bases 1240-1386

*E.coli* origin of replication: bases 1451-2234

*groESL* Promoter: bases 2256-2426

*mCherry* ORF: bases 2427-3137

*groESL* Terminator: bases 3138-3265

Multiple Cloning Site: Unique Restriction Sites: KpnI (3272), NdeI (3284), NheI (3290), NotI (3296)

3xFLAG ORF: bases 3304-3369

*incD* Terminator: bases 3370-3536

Restriction Site: SalI (3537)

SW2: bases 3549-10711

**Plasmid p2TK2_Spec_-SW2 mCh(Gro_L2_) MCS-3xFLAG sequence**

AACGGAGCCTTCTAGCTATTTTGTAAATATTTTAACAATTTAGATTCTTCAAAGCTCAGCGAGGGCGTGAAGAATCTTGTTCAGGTGTATTTGAAAAAAGTTTGTTTTAAATAGTTTTTTTAGTTAAAATGGGTCCCTAAATAATTTAAATCCGGTAGTTTTTGCGTCCGAAACATTGTTTTATAAGTGAGAAATGAGATCTGGCTAAAATCTGTCGAAGTGAGGTTTATGCGCTCACGCAACTGGTCCAGAACCTTGACCGAACGCAGCGGTGGTAACGGCGCAGTGGCGGTTTTCATGGCTTGTTATGACTGTTTTTTTGGGGTACAGTCTATGCCTCGGGCATCCAAGCAGCAAGCGCGTTACGCCGTGGGTCGATGTTTGATGTTATGGAGCAGCA

ACGATGTTACGCAGCAGGGCAGTCGCCCTAAAACAAAGTTAAACATCATGAGGGAAGCGGTGATCGCCGAAGTATCGACTCAACTATCAGAGGTAGTTGGCGTCATCGAGCGCCATCTCGAACCGACGTTGCTGGCCGTACATTTGTACGGCTCCGCAGTGGATGGCGGCCTGAAGCCACACAGTGATATTGATTTGCTGGTTACGGTGACCGTAAGGCTTGATGAAACAACGCGGCGAGCTTTGATCAACGACCTTTTGGAAACTTCGGCTTCCCCTGGAGAGAGCGAGATTCTCCGCGCTGTAGAAGTCACCATTGTTGTGCACGACGACATCATTCCGTGGCGTTATCCAGCTAAGCGCGAACTGCAATTTGGAGAATGGCAGCGCAATGACATTCTTGCAGGTATCTTCGAGCCAGCCACGATCGACATTGATCTGGCTATCTTGCTGACAAAAGCAAGAGAACATAGCGTTGCCTTGGTAGGTCCAGCGGCGGAGGAACTCTTTGATCCGGTTCCTGAACAGGATCTATTTGAGGCGCTAAATGAAACCTTAACGCTATGGAACTCGCCGCCCGACTGGGCTGGCGATGAGCGAAATGTAGTGCTTACGTTGTCCCGCATTTGGTACAGCGCAGTAACCGGCAAAATCGCGCCGAAGGATGTCGCTGCCGACTGGGCAATGGAGCGCCTGCCGGCCCAGTATCAGCCCGTCATACTTGAAGCTAGACAGGCTTATCTTGGACAAGAAGAAGATCGCTTGGCCTCGCGCGCAGATCAGTTGGAAGAATTTGTCCACTACGTGAAAGGCGAGATCACCAAGGTAGTCGGCAAATAAGCTAATTTTATTGCAATAACAGGTGCTTACTTTTAAAACTACTGATTTATTGATAAATATTGAACAATTTTTGGGAAGAATAAAGCGTCCTCTTGTGAAATTAGAGAACGCTTTATTACTTTAATTTAGTGAAACAATTTGTAACTACTGTCAGACCAAGTTTACTCATATATACTTTAGATTGATTTAAAACTTCATTTTTAATTTAAAAGGATCTAGGTGAAGATCCTTTTTGATAATCTCATGACCAAAATCCCTTAACGTGAGTTTTCGTTCCACTGAGCGTCAGACCCCGTAGAAAAGATCAAAGGATCTTCTTGAGATCCTTTTTTTCTGCGCGTAATCTGCTGCTTGCAAACAAAAAAACCACCGCTACCAGCGGTGGTTTGTTTGCCGGATCAAGAGCTACCAACTCTTTTTCCGAAGGTAACTGGCTTCAGCAGAGCGCAGATACCAAATACTGTCCTTCTAGTGTAGCCGTAGTTAGGCCACCACTTCAAGAACTCTGTAGCACCGCCTACATACCTCGCTCTGCTAATCCTGTTACCAGTGGCTGCTGCCAGTGGCGATAAGTCGTGTCTTACCGGGTTGGACTCAAGACGATAGTTACCGGATAAGGCGCAGCGGTCGGGCTGAACGGGGGGTTCGTGCACACAGCCCAGCTTGGAGCGAACGACCTACACCGAACTGAGATACCTACAGCGTGAGCTATGAGAAAGCGCCACGCTTCCCGAAGGGAGAAAGGCGGACAGGTATCCGGTAAGCGGCAGGGTCGGAACAGGAGAGCGCACGAGGGAGCTTCCAGGGGGAAACGCCTGGTATCTTTATAGTCCTGTCGGGTTTCGCCACCTCTGACTTGAGCGTCGATTTTTGTGATGCTCGTCAGGGGGGCGGAGCCTATGGAAAAACGCCAGCAACGCGGCCTTTTTACGGTTCCTGGCCTTTTGCTGGCCTTTTGCTCACATGTTCTTTCCTGCGTTATCCCCTGATTCTGTGGATAACCGTATTACACCGGTATTTTTAAAAATAGCAGTTGATCATGCCAACTGCTAAACCAGTTGCAAAAAAGCGAGGACTTTGCTATCGTTCTTCCTCTGAACGTTCTATCGTTCAAATCCCTACGTTGGTAGCGGAACAAAGCCGGACCACGGGGCCTCATAGAATATAAAAATACGAGGAGCTTAAACATGGTGAGCAAGGGCGAGGAGGATAACATGGCCATCATCAAGGAGTTCATGCGCTTCAAGGTGCACATGGAGGGCTCCGTGAACGGCCACGAGTTCGAGATCGAGGGCGAGGGCGAGGGCCGCCCCTACGAGGGCACCCAGACCGCCAAGCTGAAGGTGACCAAGGGTGGCCCCCTGCCCTTCGCCTGGGACATCCTGTCCCCTCAGTTCATGTACGGCTCCAAGGCCTACGTGAAGCACCCCGCCGACATCCCCGACTACTTGAAGCTGTCCTTCCCCGAGGGCTTCAAGTGGGAGCGCGTGATGAACTTCGAGGACGGCGGCGTGGTGACCGTGACCCAGGACTCCTCCCTGCAGGACGGCGAGTTCATCTACAAGGTGAAGCTGCGCGGCACCAACTTCCCCTCCGACGGCCCCGTAATGCAGAAGAAGACCATGGGCTGGGAGGCCTCCTCCGAGCGGATGTACCCCGAGGACGGCGCCCTGAAGGGCGAGATCAAGCAGAGGCTGAAGCTGAAGGACGGCGGCCACTACGACGCTGAGGTCAAGACCACCTACAAGGCCAAGAAGCCCGTGCAGCTGCCCGGCGCCTACAACGTCAACATCAAGTTGGACATCACCTCCCACAACGAGGACTACACCATCGTGGAACAGTACGAACGCGCCGAGGGCCGCCACTCCACCGGCGGCATGGACGAGCTGTACAAGTAGTTCCTCTAATGGGAACAAATAGATTCTTCGAGCCTCGTTTCCCAAAAGGAACGAGGCTTTTTTTTAGATTCCTAATATTTCTCTATTCCTCTATCGTAAACATCTAGTGCTTACGACCATCCTTTTCTACCGGTGGTACCCCATGGCATATGGCTAGCGCGGCCGCATGGACTACAAAGACCATGACGGTGATTATAAAGATCATGACATCGATTACAAGGATGACGATTAAGGATGACATGTGATTCGCGTAGGAAAAAGAGGAGGGAGACCTCCTCTTTTTTTTTATTTTGTAGAGTTCCGTTACTATTGGCACCCTGTGTGCAGTTAGGATGAGTAGACTAGTTCTGCAGCCTTTTACAGGGTGTTATGTTTTGCATTGCAAAAAGCTCCTAAGACGTCGACGGATCCGTTTGTTCTGGGGAAGAGGTAATTCCTCTAGTACAAACACCCACAATATTGTGATATAATTAAAATTATATTCATATTCTGTTGCCAGAAAAAACACCTTTAGGCTATATTAGAGCCAGCTTCTTTGAAGCGTTGTCTTCTCGAGAAGATTTATCGTACGCAAATATCATCTTTGCGGTTGCGTGTCCTGTGACCTTCATTATGTCGGAGTCTGAGCACCCTAGGCGTTTGTACTCCGTCACAGCGGTTGCTCGAAGCACGTGCGGGGTTATTTTAAAAGGGATTGCAGCTTGTAGTCCTGCTTGAGAGAACGTGCGGGCGATTTGCCTTAACCCCACCATTTTTCCGGAGCGAGTTACGAAGACAAAACCTCTTCGTTGACCGATGTACTCTTGTAGAAAGTGCATAAACTTCTGAGGATAAGTTATAATAATCCTCTTTTCTGTCTGACGGTTCTTAAGCTGGGAGAAAGAAATGGTAGCTTGTTGGAAACAAATCTGACTAATCTCCAAGCTTAAGACTTCAGAGGAGCGTTTACCTCCTTGGAGCATTGTCTGGGCGATCAACCAATCCCGGGCATTGATTTTTTTTAGCTCTTTTAGGAAGGATGCTGTTTGCAAACTGTTCATCGCATCCGTTTTTACTATTTCCCTGGTTTTAAAAAATGTTCGACTATTTTCTTGTTTAGAAGGTTGCGCTATAGCGACTATTCCTTGAGTCATCCTGTTTAGGAATCTTGTTAAGGAAATATAGCTTGCTGCTCGAACTTGTTTAGTACCTTCGGTCCAAGAAGTCTTGGCAGAGGAAACTTTTTTAATCGCATCTAGGATTAGATTATGATTTAAAAGGGAAAACTCTTGCAGATTCATATCCAAGGACAATAGACCAATCTTTTCTAAAGACAAAAAAGATCCTCGATATGATCTACAAGTATGTTTGTTGAGTGATGCGGTCCAATGCATAATAACTTCGAATAAGGAGAAGCTTTTCATGCGTTTCCAATAGGATTCTTGGCGAATTTTTAAAACTTCCTGATAAGACTTTTCACTATATTCTAACGACATTTCTTGCTGCAAAGATAAAATCCCTTTACCCATGAAATCCCTCGTGATATAACCTATCCGTAAAATGTCCTGATTAGTGAAATAATCAGGTTGTTAACAGGATAGCAC

GCTCGGTATTTTTTTATATAAACAGGTTGTTAACAGGATAGCACGCTCGGTATTTTTTTATATAAACATGAAAACTCGTTCCGAAATAGAAAATCGCATGCAAGATATCGAGTATGCGTTGTTAGGTAAAGCTCTGATATTTGAAGACTCTACTGAGTATATTCTGAGGCAGCTTGCTAATTATGAGTTTAAGTGTTCTCATCATAAAAACATATTCATAGTATTTAAATACTTAAAAGACAATGGATTACCTATAACTGTAGACTCGGCTTGGGAAGAGCTTTTGCGGCGTCGTATCAAAGATATGGACAAATCGTATCTCGGGTTAATGTTGCATGATGCTTTATCAAATGACAAGCTTAGATCCGTTTCTCATACGGTTTTCCTCGATGATTTGAGC

GTGTGTAGCGCTGAAGAAAATTTGAGTAATTTCATTTTCCGCTCGTTTAATGAGTACAATGAAAATCCATTGCGTAGATCTCCGTTTCTATTGCTTGAGCGTATAAAGGGAAGGCTTGATAGTGCTATAGCAAAGACTTTTTCTATTCGCAGCGCTAGAGGCCGGTCTATTTATGATATATTCTCACAGTCAGAAATTGGAGTGCTGGCTCGTATAAAAAAAAGACGAGTAGCGTTCTCTGAGAATCAAAATTCTTTCTTTGATGGCTTCCCAACAGGATACAAGGATATTGATGATAAAGGAGTTATCTTAGCTAAAGGTAATTTCGTGATTATAGCAGCTAGACCATCTATAGGGAAAACAGCTTTAGCTATAGACATGGCGATAAATCTTGCGGTTACTCAACAGCGTAGAGTTGGTTTCCTATCTCTAGAAATGAGCGCAGGTCAAATTGTTGAGCGGATTATTGCTAATTTAACAGGAATATCTGGTGAAAAATTACAAAGAGGGGATCTCTCTAAAGAAGAATTATTCCGAGTAGAAGAAGCTGGAGAAACGGTTAGAGAATCACATTTTTATATCTGCAGTGATAGTCAGTATAAGCTTAACTTAATCGCGAATCAGATCCGGTTGCTGAGAAAAGAAGATCGAGTAGACGTAATATTTATCGATTACTTGCAGTTGATCAACTCATCGGTTGGAGAAAATCGTCAAAATGAAATAGCAGATATATCTAGAACCTTAAGAGGTTTAGCCTCAGAGCTAAACATTCCTATAGTTTGTTTATCCCAACTATCTAGAAAAGTTGAGGATAGAGCAAATAAAGTTCCCATGCTTTCAGATTTGCGAGACAGCGGTCAAATAGAGCAAGACGCAGATGTGATTTTGTTTATCAATAGGAAGGAATCGTCTTCTAATTGTGAGATAACTGTTGGGAAAAATAGACATGGATCGGTTTTCTCTTCGGTATTACATTTCGATCCAAAAATTAGTAAATTCTCCGCTATTAAAAAAGTATGGTAAATTATAGTAACTGCCACTTCATCAAAAGTCCTATCCACCTTGAAAATCAGAAGTTTGGAAGAAGACCTGGTCAATCTATTAAGATATCTCCCAAATTGGCTCAAAATGGGATGGTAGAAGTTATAGGTCTTGATTTTCTTTCATCTCATTACCATGCATTAGCAGCTATCCAAAGATTACTGACCGCAACGAATTACAAGGGGAACACAAAAGGGGTTGTTTTATCCAGAGAATCAAATAGTTTTCAATTTGAAGGATGGATACCAAGAATCCGTTTTACAAAAACTGAATTCTTAGAGGCTTATGGAGTTAAGCGGTATAAAACATCCAGAAATAAGTATGAGTTTAGTGGAAAAGAAGCTGAAACTGCTTTAGAAGCCTTATACCATTTAGGACATCAACCGTTTTTAATAGTGGCAACTAGAACTCGATGGACTAATGGAACACAAATAGTAGACCGTTACCAAACTCTTTCTCCGATCATTAGGATTTACGAAGGATGGGAAGGTTTAACTGACGAAGAAAATATAGATATAGACTTAACACCTTTTAATTCACCACCTACACGGAAACATAAAGGGTTCGTTGTAGAGCCATGTCCTATCTTGGTAGATCAAATAGAATCCTACTTTGTAATCAAGCCTGCAAATGTATACCAAGAAATAAAAATGCGTTTCCCAAATGCATCAAAGTATGCTTACACATTTATCGACTGGGTGATTACAGCAGCTGCGAAA

AAGAGACGAAAATTAACTAAGGATAATTCTTGGCCAGAAAACTTGTTATTAAACGTTAACGTTAAAAGTCTTGCATATATTTTAAGGATGAATCGGTACATCTGTACAAGGAACTGGAAAAAAATCGAGTTAGCTATCGATAAATGTATAGAAATCGCCATTAAGCTTGGCTGGTTATCTAGAAGAAAACGCATTGAATTTCTGGATTCTTCTAAACTCTCTAAAAAAGAAATTCTATATCTAAATAAAGAGCGCTTTGAAGAAATAACTAAGAAATCTAAAGAACAAATGGAACAATTAGAACAAGAATCTATTAATTAATAGCAAGCTTGAAACTAAAAACCTAATTTATTTAAAGCTCAAAATAAAAAAGAGTTTTAAAATGGGAAATTCTGGTTTTTATTTGTATAACACTGAAAACTGCGTCTTTGCTGATAATATCAAAGTTGGGCAAATGACAGAGCCGCTCAAGGACCAGCAAATAATCCTTGGGACAACATCAACACCTGTCGCAGCCAAAATGACAGCTTCTGATGGAATATCTTTAACAGTCTCCAATAATTCATCAACCAATGCTTCTATTACAATTGGTTTGGATGCGGAAAAAGCTTACCAGCTTATTCTAGAAAAGTTGGGAGATCAAATTCTTGATGGAATTGCTGATACTATTGTTGATAGTACAGTCCAAGATATTTTAGACAAAATCAAAACAGACCCTTCTCTAGGTTTGTTGAAAGCTTTTAACAACTTTCCAATCACTAATAAAATTCAATGCAACGGGTTATTCACTCCCAGTAACATTGAAACTTTATTAGGAGGAACTGAAATAGGAAAATTCACAGTCACACCCAAAAGCTCTGGGAGCATGTTCTTAGTCTCAGCAGATATTATTGCATCAAGAATGGAAGGCGGCGTTGTTCTAGCTTTGGTACGAGAAGGTGATTCTAAGCCCTGCGCGATTAGTTATGGATACTCATCAGGCATTCCTAATTTATGTAGTCTAAGAACCAGTATTACTAATACAGGATTGACTCCGACAACGTATTCATTACGTGTAGGCGGTTTAGAAAGCGGTGTGGTATGGGTTAATGCCCTTTCTAATGGCAATGATATTTTAGGAATAACAAATACTTCTAATGTATCTTTTTTAGAGGTAATACCTCAAACAAACGCTTAAACAATTTTTATTGGATTTTTCTTATAGGTTTTATATTTAGAGAAAACAGTTCGAATTACGGGGTTTGTTATGCAAAATAAAAGAAAAGTGAGGGACGATTTTATTAAAATTGTTAAAGATGTGAAAAAAGATTTCCCCGAATTAGACCTAAAAATACGAGTAAACAAGGAAAAAGTAACTTTCTTAAATTCTCCCTTAGAACTCTACCATAAAAGTGTCTCACTAATTCTAGGACTGCTTCAACAAATAGAAAACTCTTTAGGATTATTCCCAGACTCTCCTGTTCTTGAAAAATTAGAGGATAACAGTTTAAAGCTAAAAAAGGCTTTGATTATGCTTATCTTGTCTAGAAAAGACATGTTTTCCAAGGCTGAATAGACAACTTACTCTAACGTTGGAGTTGATTTGCACACCTTAGTTTTTTGCTCTTTTAAGGGAGGAACTGGAAAAACAACACTTTCTCTAAACGTGGGATGCAACTTGGCCCAATTTTTAGGGAAAAAAGTGTTACTTGCTGACCTAGACCCGCAATCCAATTTATCTTCTGGATTGGGGGCTAGTGTCAGAAGTGACCAAAAAGGCTTGCACGACATAGTATACACATCAAACGATTTAAAATCAATCATTTGCGAAACAAAAAAAGATAGTGTGGACCTAATTCCTGCATCATTTTCATCCGAACAGTTTAGAGAATTGGATATTCATAGAGGACCTAGTAACAACTTAAAGTTATTTCTGAATGAGTACTGCGCTCCTTTTTATGACATCTGCATAATAGACACTCCACCTAGCCTAGGAGGGTTAACGAAAGAAGCTTTTGTTGCAGGAGACAAATTAATTGCTTGTTTAACTCCAGAACCTTTTTCTATTCTAGGGTTACAAAAGATACGTGAATTCTTAAGTTCGGTCGGAAAACCTGAAGAAGAACACATTCTTGGAATAGCTTTGTCTTTTTGGGATGATCGTAACTCGACTAACCAAATGTATATAGACATTATCGAGTCTATTTACAAAAACAAGCTTTTTTCAACAAAAATTCGTCGAGATATTTCTCTCAGCCGTTCTCTTCTTAAAGAAGATTCTGTAGCTAATGTCTATCCAAATTCTAGGGCCGCAGAAGATATTCTGAAGTTAACGCATGAAATAGCAAATATTTTGCATATCGAATATGAACGAGATTACTCTCAGAGGACAACGTGAACAAACTAAAAAAAGAAGCGGATGTCTTTTTTAAAAAAAATCAAACTGCCGCTTCTCTAGATTTTAAGAAGACGCTTCCCTCCATTGAACTATTCTCAGCAACTTTGAATTCTGAGGAAAGTCAGAGTTTGGATCGATTATTTTTATCAGAGTCCCAAAACTATTCGGATGAAGAATTTTATCAAGAAGACATCCTAGCGGTAAAACTGCTTACTGGTCAGATAAAATCCATACAGAAGCAACACGTACTTCTTTTAGGAGAAAAAATCTATAATGCTAGAAAAATCCTGAGTAAGGATCACTTCTCCTCAACAACTTTTTCATCTTGGATAGAGTTAGTTTTTAGAACTAAGTCTTCTGCTTACAATGCTCTTGCATATTACGAGCTTTTTATAAACCTCCCCAACCAAACTCTACAAAAAGAGTTTCAATCGATCCCCTATAAATCCGCATATATTTTGGCCGCTAGAAAAGGCGATTTAAAAACCAAGGTCGATGTGATAGGGAAAGTATGTGGAATGTCGAACTCATCGGCGATAAGGGTGTTGGATCAATTTCTTCCTTCATCTAGAAACAAAGACGT

TAGAGAAACGATAGATAAGTCTGATTCAGAGAAGAATCGCCAATTATCTGATTTCTTAATAGAGATACTTCGCATCATGTGTTCCGGAGTTTCTTTGTCCTCCTATAACGAAAATCTTCTACAACAGCTTTTTGAACTTTTTAAGCAAAAGAGCTGATCCTCCGTCAGCTCATATATATATATCTATTATATATATATATTTAGGGATTTGATTTCACGAGAGAGATTTGCAACTCTTGGTGGTAGACTTTGCAACTCTTGGTGGTAGACTTTGCAACTCTTGGTGGTAGACTTTGCAACTCTTGGTGGTAGACTTGGTCATAATGGACTTTTGTTAAAAAATTTCTTAAAATCTTAGAGCTCCGATTTTGAATAGCTTTGGTTAAGAAAATGGGCTCGATGGCTTTCCATAAAAGTAGATTGTTTTTAACTTTTGGGGACGCGTCGGAAATTTGGTTATCTACTTTATCTTATCTAACTAGAAAAAATTATGCGTCTGGGATTAACTTTCTTGTTTCTTTAGAGATTCTGGATTTATCGGAAACCTTGATAAAGGCTATTTCTCTTGACCACAGCGAATCTTTGTTTAAAATCAAGTCTCTAGATGTTTTTAATGGAAAAGTTGTTTCAGAGGCATCTAAACAGGCTAGAGCGGCATGCTACATATCTTTCACAAAGTTTTTGTATAGATTGACCAAGGGATATATTAAACCCGCTATTCCATTGAAAGATTTTGGAAACACTACATTTTTTAAAATCCGAGACAAAATCAAAACAGAATCGATTTCTAAGCAGGAATGGACAGTTTTTTTTGAAGCGCTCCGGATAGTGAATTATAGAGACTATTTAATCGGTAAATTGATTGTACAAGGGATCC
