# Supplementary material for: Chlamydia trachomatis and Chlamydia muridarum spectinomycin resistant vectors and a transcriptional fluorescent reporter to monitor conversion from replicative to infectious bacteria
Source: PLoS One. 2019 Jun 6;14(6):e0217753. doi: 10.1371/journal.pone.0217753 (PMC6553856; doi:10.1371/journal.pone.0217753)
Supplement: S4 Fig — (DOCX) [file pone.0217753.s005.docx]

**Plasmid p2TK2_Spec_-SW2 mCh(Gro_L2_) Tet-KpnI-IncV-NotI-3xFLAG features**

*incDEFG* Promoter: bases 1-228

*aadA* ORF (spectinomycin resistance): bases 229-1239

Terminator: bases 1240-1386

*E.coli* origin of replication: bases 1451-2234

*groESL* Promoter: bases 2256-2426

*mCherry* ORF: bases 2427-3137

*groESL* Terminator: bases 3138-3265

*tet* Repressor: bases 3272-3895

*tetA* Promoter: bases 3896-3976

Restriction Site KpnI (3977)

*IncV* ORF: bases 3983-5071

Restriction Site*:* NotI (5073)

3xFLAG: bases 5081-5146

*IncD* Terminator: bases 5147-5313

SW2: bases 5326-12488

**Plasmid p2TK2_Spec_-SW2 mCh(Gro_L2_) Tet-KpnI-IncV-NotI-3xFLAG sequence**

AACGGAGCCTTCTAGCTATTTTGTAAATATTTTAACAATTTAGATTCTTCAAAGCTCAGCGAGGGCGTGAAGAATCTTGTTCAGGTGTATTTGAAAAAAGTTTGTTTTAAATAGTTTTTTTAGTTAAAATGGGTCCCTAAATAATTTAAATCCGGTAGTTTTTGCGTCCGAAACATTGTTTTATAAGTGAGAAATGAGATCTGGCTAAAATCTGTCGAAGTGAGGTTTATGCGCTCACGCAACTGGTCCAGAACCTTGACCGAACGCAGCGGTGGTAACGGCGCAGTGGCGGTTTTCATGGCTTGTTATGACTGTTTTTTTGGGGTACAGTCTATGCCTCGGGCATCCAAGCAGCAAGCGCGTTACGCCGTGGGTCGATGTTTGATGTTATGGAGCAGCAACGATGTTACGCAGCAGGGCAGTCGCCCTAAAACAAAGTTAAACATCATGAGGGAAGCGGTGATCGCCGAAGTATCGACTCAACTATCAGAGGTAGTTGGCGTCATCGAGCGCCATCTCGAACCGACGTTGCTGGCCGTACATTTGTACGGCTCCGCAGTGGATGGCGGCCTGAAGCCACACAGTGATATTGATTTGCTGGTTACGGTGACCGTAAGGCTTGATGAAACAACGCGGCGAGCTTTGATCAACGACCTTTTGGAAACTTCGGCTTCCCCTGGAGAGAGCGAGATTCTCCGCGCTGTAGAAGTCACCATTGTTGTGCACGACGACATCATTCCGTGGCGTTATCCAGCTAAGCGCGAACTGCAATTTGGAGAATGGCAGCGCAATGACATTCTTGCAGGTATCTTCGAGCCAGCCACGATCGACATTGATCTGGCTATCTTGCTGACAAAAGCAAGAGAACATAGCGTTGCCTTGGTAGGTCCAGCGGCGGAGGAACTCTTTGATCCGGTTCCTGAACAGGATCTATTTGAGGCGCTAAATGAAACCTTAACGCTATGGAACTCGCCGCCCGACTGGGCTGGCGATGAGCGAAATGTAGTGCTTACGTTGTCCCGCATTTGGTACAGCGCAGTAACCGGCAAAATCGCGCCGAAGGATGTCGCTGCCGACTGGGCAATGGAGCGCCTGCCGGCCCAGTATCAGCCCGTCATACTTGAAGCTAGACAGGCTTATCTTGGACAAGAAGAAGATCGCTTGGCCTCGCGCGCAGATCAGTTGGAAGAATTTGTCCACTACGTGAAAGGCGAGATCACCAAGGTAGTCGGCAAATAAGCTAATTTTATTGCAATAACAGGTGCTTACTTTTAAAACTACTGATTTATTGATAAATATTGAACAATTTTTGGGAAGAATAAAGCGTCCTCTTGTGAAATTAGAGAACGCTTTATTACTTTAATTTAGTGAAACAATTTGTAACTACTGTCAGACCAAGTTTACTCATATATACTTTAGATTGATTTAAAACTTCATTTTTAATTTAAAAGGATCTAGGTGAAGATCCTTTTTGATAATCTCATGACCAAAATCCCTTAACGTGAGTTTTCGTTCCACTGAGCGTCAGACCCCGTAGAAAAGATCAAAGGATCTTCTTGAGATCCTTTTTTTCTGCGCGTAATCTGCTGCTTGCAAACAAAAAAACCACCGCTACCAGCGGTGGTTTGTTTGCCGGATCAAGAGCTACCAACTCTTTTTCCGAAGGTAACTGGCTTCAGCAGAGCGCAGATACCAAATACTGTCCTTCTAGTGTAGCCGTAGTTAGGCCACCACTTCAAGAACTCTGTAGCACCGCCTACATACCTCGCTCTGCTAATCCTGTTACCAGTGGCTGCTGCCAGTGGCGATAAGTCGTGTCTTACCGGGTTGGACTCAAGACGATAGTTACCGGATAAGGCGCAGCGGTCGGGCTGAACGGGGGGTTCGTGCACACAGCCCAGCTTGGAGCGAACGACCTACACCGAACTGAGATACCTACAGCGTGAGCTATGAGAAAGCGCCACGCTTCCCGAAGGGAGAAAGGCGGACAGGTATCCGGTAAGCGGCAGGGTCGGAACAGGAGAGCGCACGAGGGAGCTTCCAGGGGGAAACGCCTGGTATCTTTATAGTCCTGTCGGGTTTCGCCACCTCTGACTTGAGCGTCGATTTTTGTGATGCTCGTCAGGGGGGCGGAGCCTATGGAAAAACGCCAGCAACGCGGCCTTTTTACGGTTCCTGGCCTTTTGCTGGCCTTTTGCTCACATGTTCTTTCCTGCGTTATCCCCTGATTCTGTGGATAACCGTATTACACCGGTATTTTTAAAAATAGCAGTTGATCATGCCAACTGCTAAACCAGTTGCAAAAAAGCGAGGACTTTGCTATCGTTCTTCCTCTGAACGTTCTATCGTTCAAATCCCTACGTTGGTAGCGGAACAAAGCCGGACCACGGGGCCTCATAGAATATAAAAATACGAGGAGCTTAAACATGGTGAGCAAGGGCGAGGAGGATAACATGGCCATCATCAAGGAGTTCATGCGCTTCAAGGTGCACATGGAGGGCTCCGTGAACGGCCACGAGTTCGAGATCGAGGGCGAGGGCGAGGGCCGCCCCTACGAGGGCACCCAGACCGCCAAGCTGAAGGTGACCAAGGGTGGCCCCCTGCCCTTCGCCTGGGACATCCTGTCCCCTCAGTTCATGTACGGCTCCAAGGCCTACGTGAAGCACCCCGCCGACATCCCCGACTACTTGAAGCTGTCCTTCCCCGAGGGCTTCAAGTGGGAGCGCGTGATGAACTTCGAGGACGGCGGCGTGGTGACCGTGACCCAGGACTCCTCCCTGCAGGACGGCGAGTTCATCTACAAGGTGAAGCTGCGCGGCACCAACTTCCCCTCCGACGGCCCCGTAATGCAGAAGAAGACCATGGGCTGGGAGGCCTCCTCCGAGCGGATGTACCCCGAGGACGGCGCCCTGAAGGGCGAGATCAAGCAGAGGCTGAAGCTGAAGGACGGCGGCCACTACGACGCTGAGGTCAAGACCACCTACAAGGCCAAGAAGCCCGTGCAGCTGCCCGGCGCCTACAACGTCAACATCAAGTTGGACATCACCTCCCACAACGAGGACTACACCATCGTGGAACAGTACGAACGCGCCGAGGGCCGCCACTCCACCGGCGGCATGGACGAGCTGTACAAGTAGTTCCTCTAATGGGAACAAATAGATTCTTCGAGCCTCGTTTCCCAAAAGGAACGAGGCTTTTTTTTAGATTCCTAATATTTCTCTATTCCTCTATCGTAAACATCTAGTGCTTACGACCATCCTTTTCTACCGGTTTAAGACCCACTTTCACATTTAAGTTGTTTTTCTAATCCGCATATGATCAATTCAAGGCCGAATAAGAAGGCTGGCTCTGCACCTTGGTGATCAAATAATTCGATAGCTTGTCGTAATAATGGCGGCATACTATCAGTAGTAGGTGTTTCCCTTTCTTCTTTAGCGACTTGATGCTCTTGATCTTCCAATACGCAACCTAAAGTAAAATGCCCCACAGCGCTGAGTGCATATAATGCATTCTCTAATGAAAAACCTTGTTGGCATAAAAAGGCTAATTGATTTTCGAGAGTTTCATACTGTTTTTCTGTAGGCCGTGTACCTAAATGTACTTTTGCTCCATCGCGATGATTTAGTAAAGCACATCTAAAACTTTTAGCGTTATTACGTAAAAAATCTTGCCAGCTTTCCCCTTCTAAAGGGCAAAAGTGAGTATGGTGCCTATCTAACATCTCAATGGCTAAGGCGTCGAGCAAAGCCCGCTTATTTTTTACATGCCAATACAATGTAGGCTGCTCTACACCTAGCTTCTGGGCGAGTTTACGGGTTGTTAAACCTTCGATTCCGACCTCATTAAGCAGCTCTAATGCGCTGTTAATCACTTTACTTTTATCTAATCTAGACATCATTAATTCCTAATTTTTGTTGACACTCTATCATTGATAGAGTTATTTTACCACTCCCTATCAGTGATAGAGAAAAGTGAAGGTACCATGACTCCAGTAACACCAGTCCCTCCCCAATCTCCCCAACAGGTAAAAGGGCTTTTATCCAGGTTTCTGACGGCACCCGATCGTCACCCCAAACTACGCTATGTTTACGATATTGCTCTTATAGCTATTAGTATTCTCTGTATTGTGAGTATCATTCTCTGGACACAAGGGTCTGGGCTCGCCTTATTTGCAATCGCTCCAGCCTTAGCTATTGGAGCCCTAGGAGTCACTCTGCTAGTCTCAGATCTTGCCGAATCCCAGAAAAGTAAAGAGATTGCTGATACCGTTGCGGCAGTCTCTCTTCCTTTTATCCTAACAGGGACAGCTGCTGGATTGATGTTTTCTGCTATTGCCGTAGGCGGAGGCGCTGTAATCTTAGCGAATCCTCTATTCCTAATGGGCTCTATGACTCTCGGCTTTGCTCTGATGTCTCTGCATAGAGTGACCTATCAATATCTCAGTAATCGCGAGCAATGGAAACAGCAGAAGAAGCTCGAACAAGTTGAGTTAGCTGCCTGGGAGAGCCATCTTCCTAAAGAAAGCAAATCCTCCGCTCTGGAAGAGGTTCGCTATTCCCCTCGTTTGATGAAAAGAGGGAAGACTTGGCGAAAACGGGCAATCAGAAGAAAAAACTATATACCTATTCCGTTGGTCGACAAAACATTGCAAACCATGCAACCGGATGCACTCTTCTCCTCTACAACCACACATTCTACAGATAGTGAGCAGATTCTAACTTCTGTCAGTCCTCAAAGCTCAGATACCGAATCCTCCTCTTCTTCTAGCTTCCACACTCCACCAAATAGCGATAAAGAACTGTCCGACTCGAATTCTTCTGACAGCAGCTCTTCTTCTGAATATATGGATGCTCTTGAAACCGTAGCTGCAGGAGATGTCTCAGGAATAACCCCTCCATCCAAACCCTCTTCTTCTCCGAAAACGACACGCCGCGTCGTAAAGCTCTCTCGCAGCGAGAGAAATGCTCAGCATCATCGTAATAAAGACCAAGAGCAAAGACAAGACAGCAGCGAATCTTCGGATGAGGAATCCTCTTCCGATTCATCTCAAAAGAAGAAACCCTCTCGTAAAGGCGGCCGCATGGACTACAAAGACCATGACGGTGATTATAAAGATCATGACATCGATTACAAGGATGACGATTAAGGATGACATGTGATTCGCGTAGGAAAAAGAGGAGGGAGACCTCCTCTTTTTTTTTATTTTGTAGAGTTCCGTTACTATTGGCACCCTGTGTGCAGTTAGGATGAGTAGACTAGTTCTGCAGCCTTTTACAGGGTGTTATGTTTTGCATTGCAAAAAGCTCCTAAGACGTCGACGGATCCCTTGTACAATCAATTTACCGATTAAATAGTCTCTATAATTCACTATCCGGAGCGCTTCAAAAAAAACTGTCCATTCCTGCTTAGAAATCGATTCTGTTTTGATTTTGTCTCGGATTTTAAAAAATGTAGTGTTTCCAAAATCTTTCAATGGAATAGCGGGTTTAATATATCCCTTGGTCAATCTATACAAAAACTTTGTGAAAGATATGTAGCATGCCGCTCTAGCCTGTTTAGATGCCTCTGAAACAACTTTTCCATTAAAAACATCTAGAGACTTGATTTTAAACAAAGATTCGCTGTGGTCAAGAGAAATAGCCTTTATCAAGGTTTCCGATAAATCCAGAATCTCTAAAGAAACAAGAAAGTTAATCCCAGACGCATAATTTTTTCTAGTTAGATAAGATAAAGTAGATAACCAAATTTCCGACGCGTCCCCAAAAGTTAAAAACAATCTACTTTTATGGAAAGCCATCGAGCCCATTTTCTTAACCAAAGCTATTCAAAATCGGAGCTCTAAGATTTTAAGAAATTTTTTAACAAAAGTCCATTATGACCAAGTCTACCACCAAGAGTTGCAAAGTCTACCACCAAGAGTTGCAAAGTCTACCACCAAGAGTTGCAAAGTCTACCACCAAGAGTTGCAAATCTCTCTCGTGAAATCAAATCCCTAAATATATATATATAATAGATATATATATATGAGCTGACGGAGGATCAGCTCTTTTGCTTAAAAAGTTCAAAAAGCTGTTGTAGAAGATTTTCGTTATAGGAGGACAAAGAAACTCCGGAACACATGATGCGAAGTATCTCTATTAAGAAATCAGATAATTGGCGATTCTTCTCTGAATCAGACTTATCTATCGTTTCTCTAACGTCTTTGTTTCTAGATGAAGGAAGAAATTGATCCAACACCCTTATCGCCGATGAGTTCGACATTCCACATACTTTCCCTATCACATCGACCTTGGTTTTTAAATCGCCTTTTCTAGCGGCCAAAATATATGCGGATTTATAGGGGATCGATTGAAACTCTTTTTGTAGAGTTTGGTTGGGGAGGTTTATAAAAAGCTCGTAATATGCAAGAGCATTGTAAGCAGAAGACTTAGTTCTAAAAACTAACTCTATCCAAGATGAAAAAGTTGTTGAGGAGAAGTGATCCTTACTCAGGATTTTTCTAGCATTATAGATTTTTTCTCCTAAAAGAAGTACGTGTTGCTTCTGTATGGATTTTATCTGACCAGTAAGCAGTTTTACCGCTAGGATGTCTTCTTGATAAAATTCTTCATCCGAATAGTTTTGGGACTCTGATAAAAATAATCGATCCAAACTCTGACTTTCCTCAGAATTCAAAGTTGCTGAGAATAGTTCAATGGAGGGAAGCGTCTTCTTAAAATCTAGAGAAGCGGCAGTTTGATTTTTTTTAAAAAAGACATCCGCTTCTTTTTTTAGTTTGTTCACGTTGTCCTCTGAGAGTAATCTCGTTCATATTCGATATGCAAAATATTTGCTATTTCATGCGTTAACTTCAGAATATCTTCTGCGGCCCTAGAATTTGGATAGACATTAGCTACAGAATCTTCTTTAAGAAGAGAACGGCTGAGAGAAATATCTCGACGAATTTTTGTTGAAAAAAGCTTGTTTTTGTAAATAGACTCGATAATGTCTATATACATTTGGTTAGTCGAGTTACGATCATCCCAAAAAGACAAAGCTATTCCAAGAATGTGTTCTTCTTCAGGTTTTCCGACCGAACTTAAGAATTCACGTATCTTTTGTAACCCTAGAATAGAAAAAGGTTCTGGAGTTAAACAAGCAATTAATTTGTCTCCTGCAACAAAAGCTTCTTTCGTTAACCCTCCTAGGCTAGGTGGAGTGTCTATTATGCAGATGTCATAAAAAGGAGCGCAGTACTCATTCAGAAATAACTTTAAGTTGTTACTAGGTCCTCTATGAATATCCAATTCTCTAAACTGTTCGGATGAAAATGATGCAGGAATTAGGTCCACACTATCTTTTTTTGTTTCGCAAATGATTGATTTTAAATCGTTTGATGTGTATACTATGTCGTGCAAGCCTTTTTGGTCACTTCTGACACTAGCCCCCAATCCAGAAGATAAATTGGATTGCGGGTCTAGGTCAGCAAGTAACACTTTTTTCCCTAAAAATTGGGCCAAGTTGCATCCCACGTTTAGAGAAAGTGTTGTTTTTCCAGTTCCTCCCTTAAAAGAGCAAAAAACTAAGGTGTGCAAATCAACTCCAACGTTAGAGTAAGTTGTCTATTCAGCCTTGGAAAACATGTCTTTTCTAGACAAGATAAGCATAATCAAAGCCTTTTTTAGCTTTAAACTGTTATCCTCTAATTTTTCAAGAACAGGAGAGTCTGGGAATAATCCTAAAGAGTTTTCTATTTGTTGAAGCAGTCCTAGAATTAGTGAGACACTTTTATGGTAGAGTTCTAAGGGAGAATTTAAGAAAGTTACTTTTTCCTTGTTTACTCGTATTTTTAGGTCTAATTCGGGGAAATCTTTTTTCACATCTTTAACAATTTTAATAAAATCGTCCCTCACTTTTCTTTTATTTTGCATAACAAACCCCGTAATTCGAACTGTTTTCTCTAAATATAAAACCTATAAGAAAAATCCAATAAAAATTGTTTAAGCGTTTGTTTGAGGTATTACCTCTAAAAAAGATACATTAGAAGTATTTGTTATTCCTAAAATATCATTGCCATTAGAAAGGGCATTAACCCATACCACACCGCTTTCTAAACCGCCTACACGTAATGAATACGTTGTCGGAGTCAATCCTGTATTAGTAATACTGGTTCTTAGACTACATAAATTAGGAATGCCTGATGAGTATCCATAACTAATCGCGCAGGGCTTAGAATCACCTTCTCGTACCAAAGCTAGAACAACGCCGCCTTCCATTCTTGATGCAATAATATCTGCTGAGACTAAGAACATGCTCCCAGAGCTTTTGGGTGTGACTGTGAATTTTCCTATTTCAGTTCCTCCTAATAAAGTTTCAATGTTACTGGGAGTGAATAACCCGTTGCATTGAATTTTATTAGTGATTGGAAAGTTGTTAAAAGCTTTCAACAAACCTAGAGAAGGGTCTGTTTTGATTTTGTCTAAAATATCTTGGACTGTACTATCAACAATAGTATCAGCAATTCCATCAAGAATTTGATCTCCCAACTTTTCTAGAATAAGCTGGTAAGCTTTTTCCGCATCCAAACCAATTGTAATAGAAGCATTGGTTGATGAATTATTGGAGACTGTTAAAGATATTCCATCAGAAGCTGTCATTTTGGCTGCGACAGGTGTTGATGTTGTCCCAAGGATTATTTGCTGGTCCTTGAGCGGCTCTGTCATTTGCCCAACTTTGATATTATCAGCAAAGACGCAGTTTTCAGTGTTATACAAATAAAAACCAGAATTTCCCATTTTAAAACTCTTTTTTATTTTGAGCTTTAAATAAATTAGGTTTTTAGTTTCAAGCTTGCTATTAATTAATAGATTCTTGTTCTAATTGTTCCATTTGTTCTTTAGATTTCTTAGTTATTTCTTCAAAGCGCTCTTTATTTAGATATAGAATTTCTTTTTTAGAGAGTTTAGAAGAATCCAGAAATTCAATGCGTTTTCTTCTAGATAACCAGCCAAGCTTAATGGCGATTTCTATACATTTATCGATAGCTAACTCGATTTTTTTCCAGTTCCTTGTACAGATGTACCGATTCATCCTTAAAATATATGCAAGACTTTTAACGTTAACGTTTAATAACAAGTTTTCTGGCCAAGAATTATCCTTAGTTAATTTTCGTCTCTTTTTCGCAGCTGCTGTAATCACCCAGTCGATAAATGTGTAAGCATACTTTGATGCATTTGGGAAACGCATTTTTATTTCTTGGTATACATTTGCAGGCTTGATTACAAAGTAGGATTCTATTTGATCTACCAAGATAGGACATGGCTCTACAACGAACCCTTTATGTTTCCGTGTAGGTGGTGAATTAAAAGGTGTTAAGTCTATATCTATATTTTCTTCGTCAGTTAAACCTTCCCATCCTTCGTAAATCCTAATGATCGGAGAAAGAGTTTGGTAACGGTCTACTATTTGTGTTCCATTAGTCCATCGAGTTCTAGTTGCCACTATTAAAAACGGTTGATGTCCTAAATGGTATAAGGCTTCTAAAGCAGTTTCAGCTTCTTTTCCACTAAACTCATACTTATTTCTGGATGTTTTATACCGCTTAACTCCATAAGCCTCTAAGAATTCAGTTTTTGTAAAACGGATTCTTGGTATCCATCCTTCAAATTGAAAACTATTTGATTCTCTGGATAAAACAACCCCTTTTGTGTTCCCCTTGTAATTCGTTGCGGTCAGTAATCTTTGGATAGCTGCTAATGCATGGTAATGAGATGAAAGAAAATCAAGACCTATAACTTCTACCATCCCATTTTGAGCCAATTTGGGAGATATCTTAATAGATTGACCAGGTCTTCTTCCAAACTTCTGATTTTCAAGGTGGATAGGACTTTTGATGAAGTGGCAGTTACTATAATTTACCATACTTTTTTAATAGCGGAGAATTTACTAATTTTTGGATCGAAATGTAATACCGAAGAGAAAACCGATCCATGTCTATTTTTCCCAACAGTTATCTCACAATTAGAAGACGATTCCTTCCTATTGATAAACAAAATCACATCTGCGTCTTGCTCTATTTGACCGCTGTCTCGCAAATCTGAAAGCATGGGAACTTTATTTGCTCTATCCTCAACTTTTCTAGATAGTTGGGATAAACAAACTATAGGAATGTTTAGCTCTGAGGCTAAACCTCTTAAGGTTCTAGATATATCTGCTATTTCATTTTGACGATTTTCTCCAACCGATGAGTTGATCAACTGCAAGTAATCGATAAATATTACGTCTACTCGATCTTCTTTTCTCAGCAACCGGATCTGATTCGCGATTAAGTTAAGCTTATACTGACTATCACTGCAGATATAAAAATGTGATTCTCTAACCGTTTCTCCAGCTTCTTCTACTCGGAATAATTCTTCTTTAGAGAGATCCCCTCTTTGTAATTTTTCACCAGATATTCCTGTTAAATTAGCAATAATCCGCTCAACAATTTGACCTGCGCTCATTTCTAGAGATAGGAAACCAACTCTACGCTGTTGAGTAACCGCAAGATTTATCGCCATGTCTATAGCTAAAGCTGTTTTCCCTATAGATGGTCTAGCTGCTATAATCACGAAATTACCTTTAGCTAAGATAACTCCTTTATCATCAATATCCTTGTATCCTGTTGGGAAGCCATCAAAGAAAGAATTTTGATTCTCAGAGAACGCTACTCGTCTTTTTTTTATACGAGCCAGCACTCCAATTTCTGACTGTGAGAATATATCATAAATAGACCGGCCTCTAGCGCTGCGAATAGAAAAAGTCTTTGCTATAGCACTATCAAGCCTTCCCTTTATACGCTCAAGCAATAGAAACGGAGATCTACGCAATGGATTTTCATTGTACTCATTAAACGAGCGGAAAATGAAATTACTCAAATTTTCTTCAGCGCTACACACGCTCAAATCATCGAGGAAAACCGTATGAGAAACGGATCTAAGCTTGTCATTTGATAAAGCATCATGCAACATTAACCCGAGATACGATTTGTCCATATCTTTGATACGACGCCGCAAAAGCTCTTCCCAAGCCGAGTCTACAGTTATAGGTAATCCATTGTCTTTTAAGTATTTAAATACTATGAATATGTTTTTATGATGAGAACACTTAAACTCATAATTAGCAAGCTGCCTCAGAATATACTCAGTAGAGTCTTCAAATATCAGAGCTTTACCTAACAACGCATACTCGATATCTTGCATGCGATTTTCTATTTCGGAACGAGTTTTCATGTTTATATAAAAAAATACCGAGCGTGCTATCCTGTTAACAACCTGTTTATATAAAAAAATACCGAGCGTGCTATCCTGTTAACAACCTGATTATTTCACTAATCAGGACATTTTACGGATAGGTTATATCACGAGGGATTTCATGGGTAAAGGGATTTTATCTTTGCAGCAAGAAATGTCGTTAGAATATAGTGAAAAGTCTTATCAGGAAGTTTTAAAAATTCGCCAAGAATCCTATTGGAAACGCATGAAAAGCTTCTCCTTATTCGAAGTTATTATGCATTGGACCGCATCACTCAACAAACATACTTGTAGATCATATCGAGGATCTTTTTTGTCTTTAGAAAAGATTGGTCTATTGTCCTTGGATATGAATCTGCAAGAGTTTTCCCTTTTAAATCATAATCTAATCCTAGATGCGATTAAAAAAGTTTCCTCTGCCAAGACTTCTTGGACCGAAGGTACTAAACAAGTTCGAGCAGCAAGCTATATTTCCTTAACAAGATTCCTAAACAGGATGACTCAAGGAATAGTCGCTATAGCGCAACCTTCTAAACAAGAAAATAGTCGAACATTTTTTAAAACCAGGGAAATAGTAAAAACGGATGCGATGAACAGTTTGCAAACAGCATCCTTCCTAAAAGAGCTAAAAAAAATCAATGCCCGGGATTGGTTGATCGCCCAGACAATGCTCCAAGGAGGTAAACGCTCCTCTGAAGTCTTAAGCTTGGAGATTAGTCAGATTTGTTTCCAACAAGCTACCATTTCTTTCTCCCAGCTTAAGAACCGTCAGACAGAAAAGAGGATTATTATAACTTATCCTCAGAAGTTTATGCACTTTCTACAAGAGTACATCGGTCAACGAAGAGGTTTTGTCTTCGTAACTCGCTCCGGAAAAATGGTGGGGTTAAGGCAAATCGCCCGCACGTTCTCTCAAGCAGGACTACAAGCTGCAATCCCTTTTAAAATAACCCCGCACGTGCTTCGAGCAACCGCTGTGACGGAGTACAAACGCCTAGGGTGCTCAGACTCCGACATAATGAAGGTCACAGGACACGCAACCGCAAAGATGATATTTGCGTACGATAAATCTTCTCGAGAAGACAACGCTTCAAAGAAGCTGGCTCTAATATAGCCTAAAGGTGTTTTTTCTGGCAACAGAATATGAATATAATTTTAATTATATCACAATATTGTGGGTGTTTGTACTAGAGGAATTACCTCTTCCCCAGAACAAACGGATCC
