# Supplementary material for: Chlamydia trachomatis and Chlamydia muridarum spectinomycin resistant vectors and a transcriptional fluorescent reporter to monitor conversion from replicative to infectious bacteria
Source: PLoS One. 2019 Jun 6;14(6):e0217753. doi: 10.1371/journal.pone.0217753 (PMC6553856; doi:10.1371/journal.pone.0217753)
Supplement: S5 Fig — (DOCX) [file pone.0217753.s006.docx]

**Plasmid p2TK2_Spec_-Nigg features**

*incDEFG* Promoter: bases 1-228

*aadA* ORF (spectinomycin resistance): bases 229-1239

Terminator: bases 1240-1361

*E.coli* origin of replication: bases 1451-2234

Unique Restriction Sites: AgeI (2250), KpnI (2256), NdeI (2268), NotI (2280), and SgrDI (2287)

Nigg: bases 2300-9417

**Plasmid p2TK2_Spec_-Nigg sequence**

AACGGAGCCTTCTAGCTATTTTGTAAATATTTTAACAATTTAGATTCTTCAAAGCTCAGCGAGGGCGTGAAGAATCTTGTTCAGGTGTATTTGAAAAAAGTTTGTTTTAAATAGTTTTTTTAGTTAAAATGGGTCCCTAAATAATTTAAATCCGGTAGTTTTTGCGTCCGAAACATTGTTTTATAAGTGAGAAATGAGATCTGGCTAAAATCTGTCGAAGTGAGGTTTATGCGCTCACGCAACTGGTCCAGAACCTTGACCGAACGCAGCGGTGGTAACGGCGCAGTGGCGGTTTTCATGGCTTGTTATGACTGTTTTTTTGGGGTACAGTCTATGCCTCGGGCATCCAAGCAGCAAGCGCGTTACGCCGTGGGTCGATGTTTGATGTTATGGAGCAGCA

ACGATGTTACGCAGCAGGGCAGTCGCCCTAAAACAAAGTTAAACATCATGAGGGAAGCGGTGATCGCCGAAGTATCGACTCAACTATCAGAGGTAGTTGGCGTCATCGAGCGCCATCTCGAACCGACGTTGCTGGCCGTACATTTGTACGGCTCCGCAGTGGATGGCGGCCTGAAGCCACACAGTGATATTGATTTGCTGGTTACGGTGACCGTAAGGCTTGATGAAACAACGCGGCGAGCTTTGATCAACGACCTTTTGGAAACTTCGGCTTCCCCTGGAGAGAGCGAGATTCTCCGCGCTGTAGAAGTCACCATTGTTGTGCACGACGACATCATTCCGTGGCGTTATCCAGCTAAGCGCGAACTGCAATTTGGAGAATGGCAGCGCAATGACATTCTTGCAGGTATCTTCGAGCCAGCCACGATCGACATTGATCTGGCTATCTTGCTGACAAAAGCAAGAGAACATAGCGTTGCCTTGGTAGGTCCAGCGGCGGAGGAACTCTTTGATCCGGTTCCTGAACAGGATCTATTTGAGGCGCTAAATGAAACCTTAACGCTATGGAACTCGCCGCCCGACTGGGCTGGCGATGAGCGAAATGTAGTGCTTACGTTGTCCCGCATTTGGTACAGCGCAGTAACCGGCAAAATCGCGCCGAAGGATGTCGCTGCCGACTGGGCAATGGAGCGCCTGCCGGCCCAGTATCAGCCCGTCATACTTGAAGCTAGACAGGCTTATCTTGGACAAGAAGAAGATCGCTTGGCCTCGCGCGCAGATCAGTTGGAAGAATTTGTCCACTACGTGAAAGGCGAGATCACCAAGGTAGTCGGCAAATAAGCTAATTTTATTGCAATAACAGGTGCTTACTTTTAAAACTACTGATTTATTGATAAATATTGAACAATTTTTGGGAAGAATAAAGCGTCCTCTTGTGAAATTAGAGAACGCTTTATTACTTTAATTTAGTGAAACAATTTGTAACTACTGTCAGACCAAGTTTACTCATATATACTTTAGATTGATTTAAAACTTCATTTTTAATTTAAAAGGATCTAGGTGAAGATCCTTTTTGATAATCTCATGACCAAAATCCCTTAACGTGAGTTTTCGTTCCACTGAGCGTCAGACCCCGTAGAAAAGATCAAAGGATCTTCTTGAGATCCTTTTTTTCTGCGCGTAATCTGCTGCTTGCAAACAAAAAAACCACCGCTACCAGCGGTGGTTTGTTTGCCGGATCAAGAGCTACCAACTCTTTTTCCGAAGGTAACTGGCTTCAGCAGAGCGCAGATACCAAATACTGTCCTTCTAGTGTAGCCGTAGTTAGGCCACCACTTCAAGAACTCTGTAGCACCGCCTACATACCTCGCTCTGCTAATCCTGTTACCAGTGGCTGCTGCCAGTGGCGATAAGTCGTGTCTTACCGGGTTGGACTCAAGACGATAGTTACCGGATAAGGCGCAGCGGTCGGGCTGAACGGGGGGTTCGTGCACACAGCCCAGCTTGGAGCGAACGACCTACACCGAACTGAGATACCTACAGCGTGAGCTATGAGAAAGCGCCACGCTTCCCGAAGGGAGAAAGGCGGACAGGTATCCGGTAAGCGGCAGGGTCGGAACAGGAGAGCGCACGAGGGAGCTTCCAGGGGGAAACGCCTGGTATCTTTATAGTCCTGTCGGGTTTCGCCACCTCTGACTTGAGCGTCGATTTTTGTGATGCTCGTCAGGGGGGCGGAGCCTATGGAAAAACGCCAGCAACGCGGCCTTTTTACGGTTCCTGGCCTTTTGCTGGCCTTTTGCTCACATGTTCTTTCCTGCGTTATCCCCTGATTCTGTGGATAACCGTATTACACCGGTGGTACCCCATGGCATATGGCTAGCGCGGCCGCGTCGACGGATCCGTTTGTTCTGGGGAAGAGGTAATTCCTCTAGTACAAACACCCACAATATTGTGATATAATTAAAATTATATCCATTTAGTTGCCCTCAAAAGCAACTGTAGATTATATTAGGGCCATCTTCTTTGAGGCATTGTCTTCTCTAGAGGATTTATCGTACGCAAATACCATCTTTGCGGTTGTGTGTCCTGTGACCTTCATGATGTCGGAGTCCGAACACCCTAGGCGTTTGTACTCTGTCACAGCGGTTGCTCTAAGCACGTG

AGGGGTTATCTTAAATGGGATAGATGCTTGCAGTCCTGCTTGAGAGAACGTGCGGGCAATTTGTCTTAACCCCACCATTTTTCCAGAACTAGTTACGAAGACCAAACCTCTTCGTGGCCCAATGTACTCTCTTAGAGCGTGCATGAACTTCTGAGGATAAGTTATAATAATCCTCTTTTCTGTCTGACGATTCTTAAGCTGGGAGAAAGAGATAGTTGCTTGTTGAAAGCAGATCTGATCGATCTCTAAGCTTAAGACTTCAGAAGAACGCTTACCTCCTTGCAGCATAGTTTGGGCGATCAACCAATCTCTGGGATTGATTTTTTTTAGTTCTTTCAAGAAAGAAGCTGTTTGCAATCTATTCATTGCATTTGTTTTTACAATTTCTCTGGTTTTGAAAAATGTTCGGCTGTTTTCTTGTTTAGAAGGTTGTGCGATAGAAACAATTCCCTGAGTCATTCTGTTTAAAAATCTAGTCAAAGAGATATAACTAGCTGCACGAACTTGTTTGGTGCCTTCTGTCCATGAAGCTTTTGACGATGGAATCTTTTTAATTGCATCCAATATCAAGTTATGATTCAAAAGAGAAAATTCTTGTAGATTCATGTCTAAAGACAATAGCCCAATCTTTTCTAAAGCTAAAAAAGAGCCTCGGTAAGATCTACAAGTATGCTGATTTAGTGATGCAGTCCAATGCATGATAACTTCGAATAAAGAGAAGCTTCTCATGCGTTTCCAGTAAGATTCTTGTCGGATTTTTAATACTTCCTGATAAGACTTTCCGATATATTCTAATGGCATTTCTTGCTGCAAAGATAAAATCCCTTTACCCACGAAATTCCTCGTGATATAACCTAAACGCAAATGTCCTGATTAGTGAAATAATCAGGTTATCATTAGGATAGCACGCGCTGCATTTTTTTAGAAAAGCATGAAAACTAATTCTGAAATAGAAAACCGCATGCAAGATATTGAGTATGCGTTACTAGGAAAAGCTTTGGTATTTGAAGACTGTACAGAGTACATTCTTAGGCAACTTGTTAATTACGAATTCAAGTGCTCTCGCCATAAAAACATATTCATTGTTTTTAAACACTTAAAAGACAACGCTCTGCCAATAACTGTAGATTCAGCTTGGGAAGAGCTGTTAAGAAGGCGTGTCAAAGATATAGATAAGTCTTATCTCGGTATAATGTTACATGATGCCATGTTTAACGATAAGCTCAGGCCTATTTCGCATACGGTTCTTTTAGATGACTTAAGTGTATGTAGCGCTGAAGAAAATTTAACTAATTTCATTTTTCGTTCGTTTAATGAATATAACGAAAATCCATTGAGACGATCACCATTTTTACTATTAGACCGCATAAAAGATCGTCTCGACAGAACTATCGCAAAAACTTTTTCTACTCGTAGCGTTAGAGGACGATCTGTTTATGATATCTTTTCTCAAGCAGAACTCGGAGTATTAGCTCGTATAAAAAAAAGAAGGGCGGCTTATTCTGAGAATAATGATTCATTTTATGACGGCTTGCCAACCGGATATCAAGATATTGATAGTAAAGGGGTTATTTTAGCGAACGGCAATTTTGTGATAATTGCAGCTCGGCCTTCTATAGGGAAAACCGCACTCGCTATTGATATAGCTATCAATATTGCTATCCATCAACGACGTAGAGTTGGTTTTTTATCTCTTGAAATGAGTGCAGGGCAAATAGTTGAAAGAATTATTTCTAACTTAACAGGGGTATCTGGAGAGAAATTACAAAGGGGCTCTCTATCTGAAGAAGAGATTTTTTGCATTGAAGAAGCAGGAAATACTATAAGAGATTCTCATCTTTATATTTGTAGTGACAACCAATATAAGCTCAATTTGATAGCGAATCAAATTCGTTTGTTAAAACGAGATGATCGTGTCGACGTTATTTTTATCGATTACTTACAACTTATTAACTCATCTGTTGGAGAAAATCGACAAAATGAAATAGCAGATATATCTAGAACTTTAAGGGGGTTAGCTGCAGAGCTAAACATTCCTATAGTTTGTTTGTCTCAATTATCCAGAAAAGTCGAGGATAGAGCAAACAAAGTTCCTATGCTGTCAGACCTAAGAGATAGCGGTCAAATAGAACAGGATGCAGATGTAATTTTGTTCATCAATAGAAAGGAAACTTCTCCTAATTGTGAAATAACAGTGGGTAAAAATAGACATGGATCGGTTTTCTCTACTGTATTACAGTTCGATCCAAAAACAAGTAAGTTCTCTGCTATTAAAAAAGTATGGTAAATTATAGCAACTGTCACTTCATTAGAAGTCCTATTCATCTTGAGAATCAGAAGTTTGGTAGAAGACCAGGTCAATTAATCAAGATATCTCCTAAGTTAGCTCAAAATGGCTTA

GTAGAAGTCATAGGTCTTGACTTTCTTTCTTCTCATTACCACGCACTAGCTGCTATCCAGAGATTACTTACAGCTACAAATTATAAGGGGAATACAAAAGGAGTTGTATTATCAAGAGAATCAAACAGCTTCCAATTCGAAGGTTGGATTCCTCGAATTAGATTTACAAAAACAGAGTTCTTAGAAGCTTACGGCGTAAAACGATACAAAACATCTAGAAACAAATACGAATTTAGTGGGAAAGAATCTGAAACAGCTTTAGAGGCTCTGTATCATTTAGGACATCAACCTTTCTTGATAGTGGCAACCAGAACTCGATGGAATAATGGGACGCCTATTTTAGATCGTTATCAAACCCTTTCGCCTATTATTAGAATTTACGAAGGATGGGAAGGTCTAACTGATGAAGAAAATACAGAAATTGATGTAACACCATTCAATTCACCATCAACACGAAAGCATAAAGGATTCATTGTAGAACCTTGTCCCATCTTGGTAGATCAAATAGACTCTTATTTCGTAGTCAAGCCTGCGAACGTATACCAAGAAATAAAAATGCGTTTCCCAAACGCATCAAGATATGCTTACACCTTTATTGATTGGATAATTACTGCATCTGCCAAAAAGAAAAGAAAATTGACCAAAGAGAATTCTTGGCCAGAAAACTTGTCTCTGAATGTTAACGTTAAAAGCCTTGCGTATATTTTAAGGATGAATCGATATATCAGCACAAGAAACTGGAAAAAAATTGAAATGGCTATTGATAAATGTGTTGAAATAGCTATTCAACTAGGTTGGTTATCTAGTCGGAAACGAGTAGAGTTCTTAGAAGCATCTAAGCTGTCTAAAAAAGAGATCTTGTATTTAAACAAAGAACGCTTTGAAGAAATAACAAGAAAATCAAAAGAACAAATGAATCAATTCGAGCAAGAATTTAATTAAAAAATAGCAAAACTTGAAACTAAAAACCAAATTTATTTAAAGCTCAAAATAAAAAGAGTTTTTAAAATGGGAAATTCTGGTTTTTATTTGCATAACACTAGCAACTGTGTATTTGCCGACAATATTAAAGTTGGGCAAATGACAGAACCTCTTACAGATCAACAAATAATACTTGGGACATCGACAACTCCTGTCGCAGCAAAAATAACAGCTTCTGAAGGGATATCCTTAACAATAACAAACAATGCTCAAGCTAACTCTTCAGTAAATATTGGATTAGATGCTGAAAAAGCGTACCAACTTATTTTAGATAAGCTTGGCGACCAAATCTTTGATGGAATCACAGGATCCATAGTTGAGAGTGCTGTACAGGACATTATAGATAAGATTACCTCGGACCCTTCTCTAGGATTGTTGAAGGCTTTCTATAACTTCCAAATCACTGGGAAAATTCAATGTAACGGCCTATTCACATCTAGCAATGTAACAACTTTATTAGGAGGAACAGAAATAGGTAGATTTACAGTAACTCCTAGAAGTTCTGGAAGCATGTTTTTAGTTTCTGCAGATATCATTGCATCAAGAATGGAAGGTGGAGTTGTATTAGCCTTAGTAAAAGAAGGAGATACACAACCATGTGCGATTAGCTATGGCTATTCTTCTGGTGTGCCCAATTTATGTAGCTTAAAAACCTGTGTTACTAATTCCGGATCGACACCCACAACTTATTCATTACGAATAGGAGGATTAGAGAGCGGAGTTGTATGGGTTAATGCTCTATCCAATGGTAATGATATTCTTGGAATAACAAATACTTCTAACGTTTCTTTTTTGGAGGTGATACCTCAAAAAAACACTTAAATAATTTTATTGGAATTTTCTTATCGGTTTTATATTTAGAAGAAACAGTTCTAATTACGGGGGTTGTTATGCAAAACAAAAGAAAACTGAGAAACGATTTTATTAAAATTGTTAAAGATGTAGAAAAGGATTTCCCCGAGCTAGACTTGAAAATACGGGTGAATAAGGAAAGGGTTACTTTTTTAAATTCACCCTTAGAACTCTACCACAAGAGTATTTCATTAATTTTAGGCTTGTTACAACAAATAGAAAAGTCTTTGGAATTATTTCCAGATTCCCCCGTTCTTGAAAAATTAGAGGATAACAGTTTAAAGCTAAAAAAAGCGTTGATTATGCTTATTCTGTCTAGAAAAGACATGTTTTCTAAGGCAGAATAGATGTTTTACTCTAACGTTGGAGTACACTTTGCAAACCTTAGTTTTTTGCTCTTTTAAGGGTGGGACAGGAAAAACAACACTTTCCCTGAATGTAGGGTGTAATTTAGCTCAATTTTTAGGAAAGAGAGTACTTCTAATTGACCTAGATCCCCAATCAAATCTCTCATCTGGATTGGGGGCTAGCATCGAAGGCAACCATAAAGGCCTTCACGAAGTGATGTGTGCCTCAAATGATTTAAAATCAATAATTTGTAAAACAAAAAAAACTGGGGTAGACATAATCCCTGCATCATTTTTGTCAGAACAATTTAGAGAATTTTCTACAAATGGCATCCCAAGCAGCAATTTACGGCTGTTTTTAGATGAGTATTGTTCGCCTTTATATGATGTGTGCATAGTAGATACTCCACCTAGTCTTGGTGGATTAACAAAAGAAGCCTTTATTGCAGGAGACAAACTAATCGTATGTTTGATTCCTGAGCCATTTTCTATTCTCGGGCTGCAGAAAATTAGAGAATTTTTAATTTCTATAGGCAAACCTGAGGAAGAACATATTCTTGGGGTAGCACTATCTTTTTGGGATGACCGGAGTTCGACTAATCAAACGTACATAGATATCATTGAGTCAATTTACGAAAATAAGATTTTTTCAACAAAAATACGCAGAGATATTTCTTTGAGTCGTTCCCTTCTTAAAGAGGATTCTGTGATCAATGTATATCCAACTTCAAGAGCTGCAACAGATATTCTGAATTTAACACACGAAATATCTGCTCTTTTAAATTCTAAACACAAACAAGACTTTTCCCAGAGGACACTGTGAATAAACTGGAAAAGGAAGCTAGCGTCTTTTTTAAAAAAAATCAGGAATCCGTTTCTCAAGACTTTAAGAAAAAGGTTTCTTCAATTGAGATGTTTTCAACTTCTTTAAATTCGGAGGAAAACCAGAGTCTGGATCGGCTTTTTTTGTCTGAGACTCAGAATTTATCAGATGAAGAATCTTACCAAGAAGATGTTTTGTCAGTAAAACTTCTGACAAGTCAAATAAAGGCTATTCAAAAACAACACGTGCTCCTTCTTGGAGAGAAGATTTACAATGCGAGAAAGATACTAAGTAAAAGTTGTTTCTCTTCAACAACCTTTTCATCTTGGCTAGATTTAGTTTTCAGGACTAAATCATCCGCCTATAATGCGTTGGCTTATTATGAACTTTTCATAAGTCTACCAAGCACAACTTTGCAGAAAGAGTTCCAATCAATCCCGTATAAGTCTGCATATATTTTAGCTGCTAGGAAAGGAGACTTAAAAACAAAAGTCTCTGTTATAGGGAAAGTTTGTGGAATGTCCAATGCATCTGCTATCCGGGTTATGGACCAACTTCTTCCTTCATCTAGAAGTAAAGATAATCAAAGATTTTTCGAATCTGATTTAGAGAAAAATCGACAGTTATCAGATCTTCTCGTAGAACTGCTTCGCATTGTATGTTCTGGAGTTTTCTTATCTCCTTATAACGAAAACCTTCTGCAGCAGTTGTTTGAAGTCTATAAGCAAAAGAGCTGATCCGCCGTCAGCTCTTATATATATATCTATTATATATATATATTTTAGGGATTCGATTTTACGAGAGCTTCGCGCAACTCTTGGTGGTAGACCTTGCAACTCTTGGTGGTAGACCTTGCAACTCTTGGTGGTAGACCTTGCAACTCTTGGTGGTAGACTTAGTCGGGATAGACTTTTGTGTAAAAAAAAAATAAACTCTTGAGACTCTGAATCAGAGTCATATTGTTTAAGAAAAGATGAACTCAAAATTTTACCACAGAAGTAGGCTATTCCTAACTTTTGGAGACGCGTCGGAAATTTGGTTATCTACTTTATCTTATCTAACTAGAAAAAATTATGCGTCTGGGATTAACTTTCTTGTTTCTTTAGAGATTCTGGATTTATCGGAAACCTTGATAAAGGCTATTTCTCTTGACCACAGCGAATCTTTGTTTAAAATCAAGTCTCTAGATGTTTTTAATGGAAAAGTTGTTTCAGAGGCATCTAAACAGGCTAGAGCGGCATGCTACATATCTTTCACAAAGTTTTTGTATAGATTGACCAAGGGATATATTAAACCCGCTATTCCATTGAAAGATTTTGGAAACACTACATTTTTTAAAATCCGAGACAAAATCAAAACAGAATCGATTTCTAAGCAGGAATGGACAGTTTTTTTTGAAGCGCTCCGGATAGTGAATTATAGAGACTATTTAATCGGTAAATTGATTGTACAAGGGATCC
