# Supplementary material for: Chlamydia trachomatis and Chlamydia muridarum spectinomycin resistant vectors and a transcriptional fluorescent reporter to monitor conversion from replicative to infectious bacteria
Source: PLoS One. 2019 Jun 6;14(6):e0217753. doi: 10.1371/journal.pone.0217753 (PMC6553856; doi:10.1371/journal.pone.0217753)
Supplement: S7 Fig — (DOCX) [file pone.0217753.s008.docx]

**Plasmid p2TK2_Spec_-Nigg mCh(Gro_L2_) TetTC0273-3xFLAG features**

*incDEFG* Promoter: bases 1-228

*aadA* ORF (spectinomycin resistance): bases 229-1239

Terminator: bases 1310-1361

*E.coli* origin of replication: bases 1451-2234

*groESL* Promoter: bases 2256-2426

*mCherry* ORF: bases 2427-3137

*groESL* Terminator: bases 3138-3265

Restriction Site: KpnI (3287)

*tet* Repressor: bases 3293-3916

*tetA* Promoter: bases 3917-3997

*tc0273* ORF: bases 3998-5086

3xFLAG: bases 5087-5152

*incDEFG* Terminator: bases 5153-5319

Restriction Site: NotI (5320)

Unique Restriction Site: SgrDI (5327)

Nigg: bases 5340-12457

**Plasmid p2TK2_Spec_-Nigg mCh(Gro_L2_) TetTC0273-3xFLAG sequence**

AACGGAGCCTTCTAGCTATTTTGTAAATATTTTAACAATTTAGATTCTTCAAAGCTCAGCGAGGGCGTGAAGAATCTTGTTCAGGTGTATTTGAAAAAAGTTTGTTTTAAATAGTTTTTTTAGTTAAAATGGGTCCCTAAATAATTTAAATCCGGTAGTTTTTGCGTCCGAAACATTGTTTTATAAGTGAGAAATGAGATCTGGCTAAAATCTGTCGAAGTGAGGTTTATGCGCTCACGCAACTGGTCCAGAACCTTGACCGAACGCAGCGGTGGTAACGGCGCAGTGGCGGTTTTCATGGCTTGTTATGACTGTTTTTTTGGGGTACAGTCTATGCCTCGGGCATCCAAGCAGCAAGCGCGTTACGCCGTGGGTCGATGTTTGATGTTATGGAGCAGCAACGATGTTACGCAGCAGGGCAGTCGCCCTAAAACAAAGTTAAACATCATGAGGGAAGCGGTGATCGCCGAAGTATCGACTCAACTATCAGAGGTAGTTGGCGTCATCGAGCGCCATCTCGAACCGACGTTGCTGGCCGTACATTTGTACGGCTCCGCAGTGGATGGCGGCCTGAAGCCACACAGTGATATTGATTTGCTGGTTACGGTGACCGTAAGGCTTGATGAAACAACGCGGCGAGCTTTGATCAACGACCTTTTGGAAACTTCGGCTTCCCCTGGAGAGAGCGAGATTCTCCGCGCTGTAGAAGTCACCATTGTTGTGCACGACGACATCATTCCGTGGCGTTATCCAGCTAAGCGCGAACTGCAATTTGGAGAATGGCAGCGCAATGACATTCTTGCAGGTATCTTCGAGCCAGCCACGATCGACATTGATCTGGCTATCTTGCTGACAAAAGCAAGAGAACATAGCGTTGCCTTGGTAGGTCCAGCGGCGGAGGAACTCTTTGATCCGGTTCCTGAACAGGATCTATTTGAGGCGCTAAATGAAACCTTAACGCTATGGAACTCGCCGCCCGACTGGGCTGGCGATGAGCGAAATGTAGTGCTTACGTTGTCCCGCATTTGGTACAGCGCAGTAACCGGCAAAATCGCGCCGAAGGATGTCGCTGCCGACTGGGCAATGGAGCGCCTGCCGGCCCAGTATCAGCCCGTCATACTTGAAGCTAGACAGGCTTATCTTGGACAAGAAGAAGATCGCTTGGCCTCGCGCGCAGATCAGTTGGAAGAATTTGTCCACTACGTGAAAGGCGAGATCACCAAGGTAGTCGGCAAATAAGCTAATTTTATTGCAATAACAGGTGCTTACTTTTAAAACTACTGATTTATTGATAAATATTGAACAATTTTTGGGAAGAATAAAGCGTCCTCTTGTGAAATTAGAGAACGCTTTATTACTTTAATTTAGTGAAACAATTTGTAACTACTGTCAGACCAAGTTTACTCATATATACTTTAGATTGATTTAAAACTTCATTTTTAATTTAAAAGGATCTAGGTGAAGATCCTTTTTGATAATCTCATGACCAAAATCCCTTAACGTGAGTTTTCGTTCCACTGAGCGTCAGACCCCGTAGAAAAGATCAAAGGATCTTCTTGAGATCCTTTTTTTCTGCGCGTAATCTGCTGCTTGCAAACAAAAAAACCACCGCTACCAGCGGTGGTTTGTTTGCCGGATCAAGAGCTACCAACTCTTTTTCCGAAGGTAACTGGCTTCAGCAGAGCGCAGATACCAAATACTGTCCTTCTAGTGTAGCCGTAGTTAGGCCACCACTTCAAGAACTCTGTAGCACCGCCTACATACCTCGCTCTGCTAATCCTGTTACCAGTGGCTGCTGCCAGTGGCGATAAGTCGTGTCTTACCGGGTTGGACTCAAGACGATAGTTACCGGATAAGGCGCAGCGGTCGGGCTGAACGGGGGGTTCGTGCACACAGCCCAGCTTGGAGCGAACGACCTACACCGAACTGAGATACCTACAGCGTGAGCTATGAGAAAGCGCCACGCTTCCCGAAGGGAGAAAGGCGGACAGGTATCCGGTAAGCGGCAGGGTCGGAACAGGAGAGCGCACGAGGGAGCTTCCAGGGGGAAACGCCTGGTATCTTTATAGTCCTGTCGGGTTTCGCCACCTCTGACTTGAGCGTCGATTTTTGTGATGCTCGTCAGGGGGGCGGAGCCTATGGAAAAACGCCAGCAACGCGGCCTTTTTACGGTTCCTGGCCTTTTGCTGGCCTTTTGCTCACATGTTCTTTCCTGCGTTATCCCCTGATTCTGTGGATAACCGTATTACACCGGTATTTTTAAAAATAGCAGTTGATCATGCCAACTGCTAAACCAGTTGCAAAAAAGCGAGGACTTTGCTATCGTTCTTCCTCTGAACGTTCTATCGTTCAAATCCCTACGTTGGTAGCGGAACAAAGCCGGACCACGGGGCCTCATAGAATATAAAAATACGAGGAGCTTAAACATGGTGAGCAAGGGCGAGGAGGATAACATGGCCATCATCAAGGAGTTCATGCGCTTCAAGGTGCACATGGAGGGCTCCGTGAACGGCCACGAGTTCGAGATCGAGGGCGAGGGCGAGGGCCGCCCCTACGAGGGCACCCAGACCGCCAAGCTGAAGGTGACCAAGGGTGGCCCCCTGCCCTTCGCCTGGGACATCCTGTCCCCTCAGTTCATGTACGGCTCCAAGGCCTACGTGAAGCACCCCGCCGACATCCCCGACTACTTGAAGCTGTCCTTCCCCGAGGGCTTCAAGTGGGAGCGCGTGATGAACTTCGAGGACGGCGGCGTGGTGACCGTGACCCAGGACTCCTCCCTGCAGGACGGCGAGTTCATCTACAAGGTGAAGCTGCGCGGCACCAACTTCCCCTCCGACGGCCCCGTAATGCAGAAGAAGACCATGGGCTGGGAGGCCTCCTCCGAGCGGATGTACCCCGAGGACGGCGCCCTGAAGGGCGAGATCAAGCAGAGGCTGAAGCTGAAGGACGGCGGCCACTACGACGCTGAGGTCAAGACCACCTACAAGGCCAAGAAGCCCGTGCAGCTGCCCGGCGCCTACAACGTCAACATCAAGTTGGACATCACCTCCCACAACGAGGACTACACCATCGTGGAACAGTACGAACGCGCCGAGGGCCGCCACTCCACCGGCGGCATGGACGAGCTGTACAAGTAGTTCCTCTAATGGGAACAAATAGATTCTTCGAGCCTCGTTTCCCAAAAGGAACGAGGCTTTTTTTTAGATTCCTAATATTTCTCTATTCCTCTATCGTAAACATCTAGTGCTTACGACCATCCTTTTCTGGATAACCGTATTACACCGGTGGTACCTTAAGACCCACTTTCACATTTAAGTTGTTTTTCTAATCCGCATATGATCAATTCAAGGCCGAATAAGAAGGCTGGCTCTGCACCTTGGTGATCAAATAATTCGATAGCTTGTCGTAATAATGGCGGCATACTATCAGTAGTAGGTGTTTCCCTTTCTTCTTTAGCGACTTGATGCTCTTGATCTTCCAATACGCAACCTAAAGTAAAATGCCCCACAGCGCTGAGTGC

ATATAATGCATTCTCTAGTGAAAAACCTTGTTGGCATAAAAAGGCTAATTGATTTTCGAGAGTTTCATACTGTTTTTCTGTAGGCCGTGTACCTAAATGTACTTTTGCTCCATCGCGATGACTTAGTAAAGCACATCTAAAACTTTTAGCGTTATTACGTAAAAAATCTTGCCAGCTTTCCCCTTCTAAAGGGCAAAAGTGAGTATGGTGCCTATCTAACATCTCAATGGCTAAGGCGTCGAGCAAAGCCCGCTTATTTTTTACATGCCAATACAATGTAGGCTGCTCTACACCTAGCTTCTGGGCGAGTTTACGGGTTGTTAAACCTTCGATTCCGACCTCATTAAGCAGCTCTAATGCGCTGTTAATCACTTTACTTTTATCTAATCTAGACATCATTAATTCCTAATTTTTGTTGACACTCTATCATTGATAGAGTTATTTTACCACTCCCTATCAGTGATAGAGAAAAGTGAAATGGCCCCAATAACGCCAACAACATCGCCCCAGGTAAAGGGGCTTTTATCAAGGTTTTTGACAGCCCCTGATCGCCACCCCAAACTGCGCTACGTTTATGATATCTCCCTGATAGCTATCAGTATTCTCTGTATTGTTAGTATTATCCTGTGGACTCAAGGGTCCGGATTGGCATTATTTGCAATTGCCCCGGCTTTAGCTATTGGGGCTTTAGGAGTAACACTCCTTGTCTCAGACCTTGCCGAATCCCCAAAAAGTAAAGAAGTTGCAGATACTGTAGCGGCAGTCTCCCTCCCCTTTATTTTAACAGGAACGGCTGCTGGACTGATGTTCTCTGCTATTGCTGTAGGAGGAGGGGCTGTAATCCTAGCTAATCCACTATTCTTGATGGGCTCTATGACTCTAGGCTTTGCCTTAATGTCTTTGCATAAAGTGACTTATCAATATTTAAGCAACCGCTCACAATGGCAAAAACAGAATAAAATCAAACAGATTGAGTCAGCAGCCTGGGAAAACAAACTCCCTAAAGAAAGTAAAGAAAGCTCTCTACAAACAAGCGTTCGTTATTCCTCCCTCGCAAGGAAAGATAAAACTCGACGTAATAAACCCGGAATGCCCAACAAAGGGAGCCAAGTTCCTGCATCAATCGCAAACACAGAGAGATCTCTGCGATCTGAAGAAGTGCTCCACTCTCAATCTTTACTCCGCCAAAAAGAGCTTTTTCCCAACACTTCTAACATTAAGAAAGAGTTACCAAATACAAAATCGATCCTGCATACTCCATTAAACAGAAGAAGCCCTTCGGGGAGTGATTCTGATGACGTCTATTACACACCTCGGGCAGGTTTAAGTTCTGCTGAAACCTCTGCTCTGGGAGATATCTCGGGAATCAGCAGCTCCTCTACCTCTTCAAAAACGTCTACTCCTAAAGCAAAACGTCGTGTTGTAAGATCATCTCGAAGTGAACGGAATGCTCGTCATCACCGTAATAAAGAGGATCATAGACAAAATCAAGAAGAGTCTTCTGATGATGAGGACTCATCTCCCCTACCTAGCCCTAGAAGGAAAAAATACAGGTCTCGACCTAAAATGGACTACAAAGACCATGACGGTGATTATAAAGATCATGACATCGATTACAAGGATGACGATTAAGGATGACATGTGATTCGCGTAGGAAAAAGAGGAGGGAGACCTCCTCTTTTTTTTTATTTTGTAGAGTTCCGTTACTATTGGCACCCTGTGTGCAGTTAGGATGAGTAGACTAGTTCTGCAGCCTTTTACAGGGTGTTATGTTTTGCATTGCAAAAAGCTCCTAAGACGCGGCCGCGTCGACGGATCCGTTTGTTCTGGGGAAGAGGTAATTCCTCTAGTACAAACACCCACAATATTGTGATATAATTAAAATTATATCCATTTAGTTGCCCTCAAAAGCAACTGTAGATTATATTAGGGCCATCTTCTTTGAGGCATTGTCTTCTCTAGAGGATTTATCGTACGCAAATACCATCTTTGCGGTTGTG

TGTCCTGTGACCTTCATGATGTCGGAGTCCGAACACCCTAGGCGTTTGTACTCTGTCACAGCGGTTGCTCTAAGCACGTGAGGGGTTATCTTAAATGGGATAGATGCTTGCAGTCCTGCTTGAGAGAACGTGCGGGCAATTTGTCTTAACCCCACCATTTTTCCAGAACTAGTTACGAAGACCAAACCTCTTCGTGGCCCAATGTACTCTCTTAGAGCGTGCATGAACTTCTGAGGATAAGTTATAATAATCCTCTTTTCTGTCTGACGATTCTTAAGCTGGGAGAAAGAGATAGTTGCTTGTTGAAAGCAGATCTGATCGATCTCTAAGCTTAAGACTTCAGAAGAACGCTTACCTCCTTGCAGCATAGTTTGGGCGATCAACCAATCTCTGGGATTGATTTTTTTTAGTTCTTTCAAGAAAGAAGCTGTTTGCAATCTATTCATTGCATTTGTTTTTACAATTTCTCTGGTTTTGAAAAATGTTCGGCTGTTTTCTTGTTTAGAAGGTTGTGCGATAGAAACAATTCCCTGAGTCATTCTGTTTAAAAATCTAGTCAAAGAGATATAACTAGCTGCACGAACTTGTTTGGTGCCTTCTGTCCATGAAGCTTTTGACGATGGAATCTTTTTAATTGCATCCAATATCAAGTTATGATTCAAAAGAGAAAATTCTTGTAGATTCATGTCTAAAGACAATAGCCCAATCTTTTCTAAAGCTAAAAAAGAGCCTCGGTAAGATCTACAAGTATGCTGATTTAGTGATGCAGTCCAATGCATGATAACTTCGAATAAAGAGAA

GCTTCTCATGCGTTTCCAGTAAGATTCTTGTCGGATTTTTAATACTTCCTGATAAGACTTTCCGATATATTCTAATGGCATTTCTTGCTGCAAAGATAAAATCCCTTTACCCACGAAATTCCTCGTGATATAACCTAAACGCAAATGTCCTGATTAGTGAAATAATCAGGTTATCATTAGGATAGCACGCGCTGCATTTTTTTAGAAAAGCATGAAAACTAATTCTGAAATAGAAAACCGCATGCAAGATATTGAGTATGCGTTACTAGGAAAAGCTTTGGTATTTGAAGACTGTACAGAGTACATTCTTAGGCAACTTGTTAATTACGAATTCAAGTGCTCTCGCCATAAAAACATATTCATTGTTTTTAAACACTTAAAAGACAACGCTCTGCCAATA

ACTGTAGATTCAGCTTGGGAAGAGCTGTTAAGAAGGCGTGTCAAAGATATAGATAAGTCTTATCTCGGTATAATGTTACATGATGCCATGTTTAACGATAAGCTCAGGCCTATTTCGCATACGGTTCTTTTAGATGACTTAAGTGTATGTAGCGCTGAAGAAAATTTAACTAATTTCATTTTTCGTTCGTTTAATGAATATAACGAAAATCCATTGAGACGATCACCATTTTTACTATTAGACCGCATAAAAGATCGTCTCGACAGAACTATCGCAAAAACTTTTTCTACTCGTAGCGTTAGAGGACGATCTGTTTATGATATCTTTTCTCAAGCAGAACTCGGAGTATTAGCTCGTATAAAAAAAAGAAGGGCGGCTTATTCTGAGAATAATGATTCATTTTATGACGGCTTGCCAACCGGATATCAAGATATTGATAGTAAAGGGGTTATTTTAGCGAACGGCAATTTTGTGATAATTGCAGCTCGGCCTTCTATAGGGAAAACCGCACTCGCTATTGATATAGCTATCAATATTGCTATCCATCAACGACGTAGAGTTGGTTTTTTATCTCTTGAAATGAGTGCAGGGCAAATAGTTGAAAGAATTATTTCTAACTTAACAGGGGTATCTGGAGAGAAATTACAAAGGGGCTCTCTATCTGAAGAAGAGATTTTTTGCATTGAAGAAGCAGGAAATACTATAAGAGATTCTCATCTTTATATTTGTAGTGACAACCAATATAAGCTCAATTTGATAGCGAATCAAATTCGTTTGTTAAAACGAGATGATCGTGTCGACGTTATTTTTATCGATTACTTACAACTTATTAACTCATCTGTTGGAGAAAATCGACAAAATGAAATAGCAGATATATCTAGAACTTTAAGGGGGTTAGCTGCAGAGCTAAACATTCCTATAGTTTGTTTGTCTCAATTATCCAGAAAAGTCGAGGATAGAGCAAACAAAGTTCCTATGCTGTCAGACCTAAGAGATAGCGGTCAAATAGAACAGGATGCAGATGTAATTTTGTTCATCAATAGAAAGGAAACTTCTCCTAATTGTGAAATAACAGTGGGTAAAAATAGACATGGATCGGTTTTCTCTACTGTATTACAGTTCGATCCAAAAACAAGTAAGTTCTCTGCTATTAAAAAAGTATGGTAAATTATAGCAACTGTCACTTCATTAGAAGTCCTATTCATCTTGAGAATCAGAAGTTTGGTAGAAGACCAGGTCAATTAATCAAGATATCTCCTAAGTTAGCTCAAAATGGCTTAGTAGAAGTCATAGGTCTTGACTTTCTTTCTTCTCATTACCACGCACTAGCTGCTATCCAGAGATTACTTACAGCTACAAATTATAAGGGGAATACAAAAGGAGTTGTATTATCAAGAGAATCAAACAGCTTCCAATTCGAAGGTTGGATTCCTCGAATTAGATTTACAAAAACAGAGTTCTTAGAAGCTTACGGCGTAAAACGATACAAAACATCTAGAAACAAATACGAATTTAGTGGGAAAGAATCTGAAACAGCTTTAGAGGCTCTGTATCATTTAGGACATCAACCTTTCTTGATAGTGGCAACCAGAACTCGATGGAATAATGGGACGCCTATTTTAGATCGTTATCAAACCCTTTCGCCTATTATTAGAATTTACGAAGGATGGGAAGGTCTAACTGATGAAGAAAATACAGAAATTGATGTAACACCATTCAATTCACCATCAACACGAAAGCATAAAGGATTCATTGTAGAACCTTGTCCCATCTTGGTAGATCAAATAGACTCTTATTTCGTAGTCAAGCCTGCGAACGTATACCAAGAAATAAAAATGCGTTTCCCAAACGCATCAAGATATGCTTACACCTTTATTGATTGGATAATTACTGCATCTGCCAAAAAGAAAAGAAAATTGACCAAAGAGAATTCTTGGCCAGAAAACTTGTCTCTGAATGTTAACGTTAAAAGCCTTGCGTATATTTTAAGGATGAATCGATATATCAGCACAAGAAACTGGAAAAAAATTGAAATGGCTATTGATAAATGTGTTGAAATAGCTATTCAACTAGGTTGGTTATCTAGTCGGAAACGAGTAGAGTTCTTAGAAGCATCTAAGCTGTCTAAAAAAGAGATCTTGTATTTAAACAAAGAACGCTTTGAAGAAATAACAAGAAAATCAAAAGAACAAATGAATCAATTCGAGCAAGAATTTAATTAAAAAATAGCAAAACTTGAAACTAAAAACCAAATTTATTTAAAGCTCAAAATAAAAAGAGTTTTTAAAATGGGAAATTCTGGTTTTTATTTGCATAACACTAGCAACTGTGTATTTGCCGACAATATTAAAGTTGGGCAAATGACAGAACCTCTTACAGATCAACAAATAATACTTGGGACATCGACAACTCCTGTCGCAGCAAAAATAACAGCTTCTGAAGGGATATCCTTAACAATAACAAACAATGCTCAAGCTAACTCTTCAGTAAATATTGGATTAGATGCTGAAAAAGCGTACCAACTTATTTTAGATAAGCTTGGCGACCAAATCTTTGATGGAATCACAGGATCCATAGTTGAGAGTGCTGTACAGGACATTATAGATAAGATTACCTCGGACCCTTCTCTAGGATTGTTGAAGGCTTTCTATAACTTCCAAATCACTGGGAAAATTCAATGTAACGGCCTATTCACATCTAGCAATGTAACAACTTTATTAGGAGGAACAGAAATAGGTAGATTTACAGTAACTCCTAGAAGTTCTGGAAGCATGTTTTTAGTTTCTGCAGATATCATTGCATCAAGAATGGAAGGTGGAGTTGTATTAGCCTTAGTAAAAGAAGGAGATACACAACCATGTGCGATTAGCTATGGCTATTCTTCTGGTGTGCCCAATTTATGTAGCTTAAAAACCTGTGTTACTAATTCCGGATCGACACCCACAACTTATTCATTACGAATAGGAGGATTAGAGAGCGGAGTTGTATGGGTTAATGCTCTATCCAATGGTAATGATATTCTTGGAATAACAAATACTTCTAACGTTTCTTTTTTGGAGGTGATACCTCAAAAAAACACTTAAATAATTTTATTGGAATTTTCTTATCGGTTTTATATTTAGAAGAAACAGTTCTAATTACGGGGGTTGTTATGCAAAACAAAAGAAAACTGAGAAACGATTTTATTAAAATTGTTAAAGATGTAGAAAAGGATTTCCCCGAGCTAGACTTGAAAATACGGGTGAATAAGGAAAGGGTTACTTTTTTAAATTCACCCTTAGAACTCTACCACAAGAGTATTTCATTAATTTTAGGCTTGTTACAACAAATAGAAAAGTCTTTGGAATTATTTCCAGATTCCCCCGTTCTTGAAAAATTAGAGGATAACAGTTTAAAGCTAAAAAAAGCGTTGATTATGCTTATTCTGTCTAGAAAAGACATGTTTTCTAAGGCAGAATAGATGTTTTACTCTAACGTTGGAGTACACTTTGCAAACCTTAGTTTTTTGCTCTTTTAAGGGTGGGACAGGAAAAACAACACTTTCCCTGAATGTAGGGTGTAATTTAGCTCAATTTTTAGGAAAGAGAGTACTTCTAATTGACCTAGATCCCCAATCAAATCTCTCATCTGGATTGGGGGCTAGCATCGAAGGCAACCATAAAGGCCTTCACGAAGTGATGTGTGCCTCAAATGATTTAAAATCAATAATTTGTAAAACAAAAAAAACTGGGGTAGACATAATCCCTGCATCATTTTTGTCAGAACAATTTAGAGAATTTTCTACAAATGGCATCCCAAGCAGCAATTTACGGCTGTTTTTAGATGAGTATTGTTCGCCTTTATATGATGTGTGCATAGTAGATACTCCACCTAGTCTTGGTGGATTAACAAAAGAAGCCTTTATTGCAGGAGACAAACTAATCGTATGTTTGATTCCTGAGCCATTTTCTATTCTCGGGCTGCAGAAAATTAGAGAATTTTTAATTTCTATAGGCAAACCTGAGGAAGAACATATTCTTGGGGTAGCACTATCTTTTTGGGATGACCGGAGTTCGACTAATCAAACGTACATAGATATCATTGAGTCAATTTACGAAAATAAGATTTTTTCAACAAAAATACGCAGAGATATTTCTTTGAGTCGTTCCCTTCTTAAAGAGGATTCTGTGATCAATGTATATCCAACTTCAAGAGCTGCAACAGATATTCTGAATTTAACACACGAAATATCTGCTCTTTTAAATTCTAAACACAAACAAGACTTTTCCCAGAGGACACTGTGAATAAACTGGAAAAGGAAGCTAGCGTCTTTTTTAAAAAAAATCAGGAATCCGTTTCTCAAGACTTTAAGAAAAAGGTTTCTTCAATTGAGATGTTTTCAACTTCTTTAAATTCGGAGGAAAACCAGAGTCTGGATCGGCTTTTTTTGTCTGAGACTCAGAATTTATCAGATGAAGAATCTTACCAAGAAGATGTTTTGTCAGTAAAACTTCTGACAAGTCAAATAAAGGCTATTCAAAAACAACACGTGCTCCTTCTTGGAGAGAAGATTTACAATGCGAGAAAGATACTAAGTAAAAGTTGTTTCTCTTCAACAACCTTTTCATCTTGGCTAGATTTAGTTTTCAGGACTAAATCATCCGCCTATAATGCGTTGGCTTATTATGAACTTTTCATAAGTCTACCAAGCACAACTTTGCAGAAAGAGTTCCAATCAATCCCGTATAAGTCTGCATATATTTTAGCTGCTAGGAAAGGAGACTTAAAAACAAAAGTCTCTGTTATAGGGAAAGTTTGTGGAATGTCCAATGCATCTGCTATCCGGGTTATGGACCAACTTCTTCCTTCATCTAGAAGTAAAGATAATCAAAGATTTTTCGAATCTGATTTAGAGAAAAATCGACAGTTATCAGATCTTCTCGTAGAACTGCTTCGCATTGTATGTTCTGGAGTTTTCTTATCTCCTTATAACGAAAACCTTCTGCAGCAGTTGTTTGAAGTCTATAAGCAAAAGAGCTGATCCGCCGTCAGCTCTTATATATATATCTATTATATATATATATTTTAGGGATTCGATTTTACGAGAGCTTCGCGCAACTCTTGGTGGTAGACCTTGCAACTCTTGGTGGTAGACCTTGCAACTCTTGGTGGTAGACCTTGCAACTCTTGGTGGTAGACTTAGTCGGGATAGACTTTTGTGTAAAAAAAAAATAAACTCTTGAGACTCTGAATCAGAGTCATATTGTTTAAGAAAAGATGAACTCAAAATTTTACCACAGAAGTAGGCTATTCCTAACTTTTGGAGACGCGTCGGAAATTTGGTTATCTACTTTATCTTATCTAACTAGAAAAAATTATGCGTCTGGGATTAACTTTCTTGTTTCTTTAGAGATTCTGGATTTATCGGAAACCTTGATAAAGGCTATTTCTCTTGACCACAGCGAATCTTTGTTTAAAATCAAGTCTCTAGATGTTTTTAATGGAAAAGTTGTTTCAGAGGCATCTAAACAGGCTAGAGCGGCATGCTACATATCTTTCACAAAGTTTTTGTATAGATTGACCAAGGGATATATTAAACCCGCTATTCCATTGAAAGATTTTGGAAACACTACATTTTTTAAAATCCGAGACAAAATCAAAACAGAATCGATTTCTAAGCAGGAATGGACAGTTTTTTTTGAAGCGCTCCGGATAGTGAATTATAGAGACTATTTAATCGGTAAATTGATTGTACAAGGGATCC
