# Supplementary material for: Chlamydia trachomatis and Chlamydia muridarum spectinomycin resistant vectors and a transcriptional fluorescent reporter to monitor conversion from replicative to infectious bacteria
Source: PLoS One. 2019 Jun 6;14(6):e0217753. doi: 10.1371/journal.pone.0217753 (PMC6553856; doi:10.1371/journal.pone.0217753)
Supplement: S8 Fig — (DOCX) [file pone.0217753.s009.docx]

**Plasmid p2TK2-SW2 mCh(Gro_L2_) GFP(OmcA_L2_) Features**

Promoter: bases 1-241

*bla* ORF (Ampicillin resistance): bases 242-1102

*E.coli* origin of replcation: bases 1167-1950

*groESL* Promoter: bases 1972-2142

*mCherry* ORF: bases 2143-2853

*groESL* Terminator: bases 2854-2981

*C. trachomatis* (L2) *omcA* Promoter: bases 2994-3329

*gfp* ORF: bases 3330-4061

*incDEFG* Terminator: bases 4062-4228

Unique Restriction Sites: KpnI (2988), NotI (4229), and SalI (4237)

SW2: bases 4249-11411

**Plasmid p2TK2-SW2 mCh(Gro_L2_) GFP(OmcA_L2_) Sequence**

GACCCAGTCACGTAGCGATAGCGGAGTGTATAATTCTTGAAGACGAAAGGGCCTCGTGATACGCCTATTTTTATAGGTTAATGTCATGATAATAATGGTTTCTTAGACGTCAGGTGGCACTTTTCGGGGAAATGTGCGCGGAACCCCTATTTGTTTATTTTTCTAAATACATTCAAATATGTATCCGCTCATGAGACAATAACCCTGATAAATGCTTCAATAATATTGAAAAAGGAAGAGTATGAGTATTCAACATTTCCGTGTCGCCCTTATTCCCTTTTTTGCGGCATTTTGCCTTCCTGTTTTTGCTCACCCAGAAACGCTGGTGAAAGTAAAAGATGCTGAAGATCAGTTGGGTGCACGAGTGGGTTACATCGAACTGGATCTCAACAGCGGTAAGATCCTTGAGAGTTTTCGCCCCGAAGAACGTTTTCCAATGATGAGCACTTTTAAAGTTCTGCTATGTGGCGCGGTATTATCCCGTGTTGACGCCGGGCAAGAGCAACTCGGTCGCCGCATACACTATTCTCAGAATGACTTGGTTGAGTACTCACCAGTCACAGAAAAGCATCTTACGGATGGCATGACAGTAAGAGAATTATGCAGTGCTGCCATAACCATGAGTGATAACACTGCGGCCAACTTACTTCTGACAACGATCGGAGGACCGAAGGAGCTAACCGCTTTTTTGCACAACATGGGGGATCATGTAACTCGCCTTGATCGTTGGGAACCGGAGCTGAATGAAGCCATACCAAACGACGAGCGTGACACCACGATGCCTGCAGCAATGGCAACAACGTTGCGCAAACTATTAACTGGCGAACTACTTACTCTAGCTTCCCGGCAACAATTAATAGACTGGATGGAGGCGGATAAAGTTGCAGGACCACTTCTGCGCTCGGCCCTTCCGGCTGGCTGGTTTATTGCTGATAAATCTGGAGCCGGTGAGCGTGGGTCTCGCGGTATCATTGCAGCACTGGGGCCAGATGGTAAGCCCTCCCGTATCGTAGTTATCTACACGACGGGGAGTCAGGCAACTATGGATGAACGAAATAGACAGATCGCTGAGATAGGTGCCTCACTGATTAAGCATTGGTAACTGTCAGACCAAGTTTACTCATATATACTTTAGATTGATTTAAAACTTCATTTTTAATTTAAAAGGATCTAGGTGAAGATCCTTTTTGATAATCTCATGACCAAAATCCCTTAACGTGAGTTTTCGTTCCACTGAGCGTCAGACCCCGTAGAAAAGATCAAAGGATCTTCTTGAGATCCTTTTTTTCTGCGCGTAATCTGCTGCTTGCAAACAAAAAAACCACCGCTACCAGCGGTGGTTTGTTTGCCGGATCAAGAGCTACCAACTCTTTTTCCGAAGGTAACTGGCTTCAGCAGAGCGCAGATACCAAATACTGTCCTTCTAGTGTAGCCGTAGTTAGGCCACCACTTCAAGAACTCTGTAGCACCGCCTACATACCTCGCTCTGCTAATCCTGTTACCAGTGGCTGCTGCCAGTGGCGATAAGTCGTGTCTTACCGGGTTGGACTCAAGACGATAGTTACCGGATAAGGCGCAGCGGTCGGGCTGAACGGGGGGTTCGTGCACACAGCCCAGCTTGGAGCGAACGACCTACACCGAACTGAGATACCTACAGCGTGAGCTATGAGAAAGCGCCACGCTTCCCGAAGGGAGAAAGGCGGACAGGTATCCGGTAAGCGGCAGGGTCGGAACAGGAGAGCGCACGAGGGAGCTTCCAGGGGGAAACGCCTGGTATCTTTATAGTCCTGTCGGGTTTCGCCACCTCTGACTTGAGCGTCGATTTTTGTGATGCTCGTCAGGGGGGCGGAGCCTATGGAAAAACGCCAGCAACGCGGCCTTTTTACGGTTCCTGGCCTTTTGCTGGCCTTTTGCTCACATGTTCTTTCCTGCGTTATCCCCTGATTCTGTGGATAACCGTATTACACCGGTATTTTTAAAAATAGCAGTTGATCATGCCAACTGCTAAACCAGTTGCAAAAAAGCGAGGACTTTGCTATCGTTCTTCCTCTGAACGTTCTATCGTTCAAATCCCTACGTTGGTAGCGGAACAAAGCCGGACCACGGGGCCTCATAGAATATAAAAATACGAGGAGCTTAAACATGGTGAGCAAGGGCGAGGAGGATAACATGGCCATCATCAAGGAGTTCATGCGCTTCAAGGTGCACATGGAGGGCTCCGTGAACGGCCACGAGTTCGAGATCGAGGGCGAGGGCGAGGGCCGCCCCTACGAGGGCACCCAGACCGCCAAGCTGAAGGTGACCAAGGGTGGCCCCCTGCCCTTCGCCTGGGACATCCTGTCCCCTCAGTTCATGTACGGCTCCAAGGCCTACGTGAAGCACCCCGCCGACATCCCCGACTACTTGAAGCTGTCCTTCCCCGAGGGCTTCAAGTGGGAGCGCGTGATGAACTTCGAGGACGGCGGCGTGGTGACCGTGACCCAGGACTCCTCCCTGCAGGACGGCGAGTTCATCTACAAGGTGAAGCTGCGCGGCACCAACTTCCCCTCCGACGGCCCCGTAATGCAGAAGAAGACCATGGGCTGGGAGGCCTCCTCCGAGCGGATGTACCCCGAGGACGGCGCCCTGAAGGGCGAGATCAAGCAGAGGCTGAAGCTGAAGGACGGCGGCCACTACGACGCTGAGGTCAAGACCACCTACAAGGCCAAGAAGCCCGTGCAGCTGCCCGGCGCCTACAACGTCAACATCAAGTTGGACATCACCTCCCACAACGAGGACTACACCATCGTGGAACAGTACGAACGCGCCGAGGGCCGCCACTCCACCGGCGGCATGGACGAGCTGTACAAGTAGTTCCTCTAATGGGAACAAATAGATTCTTCGAGCCTCGTTTCCCAAAAGGAACGAGGCTTTTTTTTAGATTCCTAATATTTCTCTATTCCTCTATCGTAAACATCTAGTGCTTACGACCATCCTTTTCTACCGGTGGTACCTTTTTCATCCCTCCGATTCTTGTATTATATAGTTAGTCTGTTTGTTATCTATAGTTTAGATAGATTTTAGTTTTAGTTTATTTATTCTATCGAAGTTTGTTTTGTTTGTAGGCAAACTTATCTCTTTGAATCCGAGCTGTTTATTATTTTATTTTGTTTGCTTTGATTTGCTAATTACCTGTTATTAGACGATTTGTTTTAAAAAACAATTGATATAATTTTTATTTTATAATGTAATATTGTCTATGAGGGCTAGTTTCTTTTATTATTAAAAGAATTGCTTTTATCGATAAAAGAAACTTCAAGAGCCCTTTTCTAGAAAGGAGTCTGGAAGTTATGAGTAAAGGAGAAGCACTTTTCACTGGAGTTGTCCCAATTCTTGTTGAATTAGATGGTGATGTTAATGGGCACAAATTTTCTGTCAGTGGAGAGGGTGAAGGTGATGCAACATACGGAAAACTTACCCTTAAATTTATTTGCACTACTGGAAAACTACCTGTTCCATGGCCAACACTTGTCACTACTCTTACGTATGGTGTTCAATGCTTTTCAAGATACCCAGATCATATGAAACGGCATGACTTTTTCAAGAGTGCCATGCCCGAAGGTTATGTACAGGAAAGAACTATATTTTTCAAAGATGACGGGAACTACAAGACACGTGCTGAAGTCAAGTTTGAAGGTGATACCCTTGTTAATAGAATCGAGTTAAAAGGTATTGATTTTAAAGAAGATGGAAACATTCTTGGACACAAATTGGAATACAACTATAACTCACACAATGTATACATCATGGCAGACAAACAAAAGAATGGAATCAAAGTTAACTTCAAAATTAGACACAACATTGAAGATGGAAGCGTTCAACTAGCAGACCATTATCAACAAAATACTCCAATTGGCGATGGCCCTGTCCTTTTACCAGACAACCATTACCTGTCCACACAATCTGCCCTTTCGAAAGATCCCAACGAAAAGAGAGACCACATGGTCCTTCTTGAGTTTGTAACAGCTGCTGGGATTACACATGGCATGGATGAACTATACAAGTCCGGACTCAGATCTTAAGGATGACATGTGATTCGCGTAGGAAAAAGAGGAGGGAGACCTCCTCTTTTTTTTTATTTTGTAGAGTTCCGTTACTATTGGCACCCTGTGTGCAGTTAGGATGAGTAGACTAGTTCTGCAGCCTTTTACAGGGTGTTATGTTTTGCATTGCAAAAAGCTCCTAAGACGCGGCCGCGTCGACGGATCCGTTTGTTCTGGGGAAGAGGTAATTCCTCTAGTACAAACACCCACAATATTGTGATATAATTAAAATTATATTCATATTCTGTTGCCAGAAAAAACACCTTTAGGCTATATTAGAGCCAGCTTCTTTGAAGCGTTGTCTTCTCGAGAAGATTTATCGTACGCAAATATCATCTTTGCGGTTGCGTGTCCTGTGACCTTCATTATGTCGGAGTCTGAGCACCCTAGGCGTTTGTACTCCGTCACAGCGGTTGCTCGAAGCACGTGCGGGGTTATTTTAAAAGGGATTGCAGCTTGTAGTCCTGCTTGAGAGAACGTGCGGGCGATTTGCCTTAACCCCACCATTTTTCCGGAGCGAGTTACGAAGACAAAACCTCTTCGTTGACCGATGTACTCTTGTAGAAAGTGCATAAACTTCTGAGGATAAGTTATAATAATCCTCTTTTCTGTCTGACGGTTCTTAAGCTGGGAGAAAGAAATGGTAGCTTGTTGGAAACAAATCTGACTAATCTCCAAGCTTAAGACTTCAGAGGAGCGTTTACCTCCTTGGAGCATTGTCTGGGCGATCAACCAATCCCGGGCATTGATTTTTTTTAGCTCTTTTAGGAAGGATGCTGTTTGCAAACTGTTCATCGCATCCGTTTTTACTATTTCCCTGGTTTTAAAAAATGTTCGACTATTTTCTTGTTTAGAAGGTTGCGCTATAGCGACTATTCCTTGAGTCATCCTGTTTAGGAATCTTGTTAAGGAAATATAGCTTGCTGCTCGAACTTGTTTAGTACCTTCGGTCCAAGAAGTCTTGGCAGAGGAAACTTTTTTAATCGCATCTAGGATTAGATTATGATTTAAAAGGGAAAACTCTTGCAGATTCATATCCAAGGACAATAGACCAATCTTTTCTAAAGACAAAAAAGATCCTCGATATGATCTACAAGTATGTTTGTTGAGTGATGCGGTCCAATGCATAATAACTTCGAATAAGGAGAAGCTTTTCATGCGTTTCCAATAGGATTCTTGGCGAATTTTTAAAACTTCCTGATAAGACTTTTCACTATATTCTAACGACATTTCTTGCTGCAAAGATAAAATCCCTTTACCCATGAAATCCCTCGTGATATAACCTATCCGTAAAATGTCCTGATTAGTGAAATAATCAGGTTGTTAACAGGATAGCACGCTCGGTATTTTTTTATATAAACAGGTTGTTAACAGGATAGCACGCTCGGTATTTTTTTATATAAACATGAAAACTCGTTCCGAAATAGAAAATCGCATGCAAGATATCGAGTATGCGTTGTTAGGTAAAGCTCTGATATTTGAAGACTCTACTGAGTATATTCTGAGGCAGCTTGCTAATTATGAGTTTAAGTGTTCTCATCATAAAAACATATTCATAGTATTTAAATACTTAAAAGACAATGGATTACCTATAACTGTAGACTCGGCTTGGGAAGAGCTTTTGCGGCGTCGTATCAAAGATATGGACAAATCGTATCTCGGGTTAATGTTGCATGATGCTTTATCAAATGACAAGCTTAGATCCGTTTCTCATACGGTTTTCCTCGATGATTTGAGCGTGTGTAGCGCTGAAGAAAATTTGAGTAATTTCATTTTCCGCTCGTTTAATGAGTACAATGAAAATCCATTGCGTAGATCTCCGTTTCTATTGCTTGAGCGTATAAAGGGAAGGCTTGATAGTGCTATAGCAAAGACTTTTTCTATTCGCAGCGCTAGAGGCCGGTCTATTTATGATATATTCTCACAGTCAGAAATTGGAGTGCTGGCTCGTATAAAAAAAAGACGAGTAGCGTTCTCTGAGAATCAAAATTCTTTCTTTGATGGCTTCCCAACAGGATACAAGGATATTGATGATAAAGGAGTTATCTTAGCTAAAGGTAATTTCGTGATTATAGCAGCTAGACCATCTATAGGGAAAACAGCTTTAGCTATAGACATGGCGATAAATCTTGCGGTTACTCAACAGCGTAGAGTTGGTTTCCTATCTCTAGAAATGAGCGCAGGTCAAATTGTTGAGCGGATTATTGCTAATTTAACAGGAATATCTGGTGAAAAATTACAAAGAGGGGATCTCTCTAAAGAAGAATTATTCCGAGTAGAAGAAGCTGGAGAAACGGTTAGAGAATCACATTTTTATATCTGCAGTGATAGTCAGTATAAGCTTAACTTAATCGCGAATCAGATCCGGTTGCTGAGAAAAGAAGATCGAGTAGACGTAATATTTATCGATTACTTGCAGTTGATCAACTCATCGGTTGGAGAAAATCGTCAAAATGAAATAGCAGATATATCTAGAACCTTAAGAGGTTTAGCCTCAGAGCTAAACATTCCTATAGTTTGTTTATCCCAACTATCTAGAAAAGTTGAGGATAGAGCAAATAAAGTTCCCATGCTTTCAGATTTGCGAGACAGCGGTCAAATAGAGCAAGACGCAGATGTGATTTTGTTTATCAATAGGAAGGAATCGTCTTCTAATTGTGAGATAACTGTTGGGAAAAATAGACATGGATCGGTTTTCTCTTCGGTATTACATTTCGATCCAAAAATTAGTAAATTCTCCGCTATTAAAAAAGTATGGTAAATTATAGTAACTGCCACTTCATCAAAAGTCCTATCCACCTTGAAAATCAGAAGTTTGGAAGAAGACCTGGTCAATCTATTAAGATATCTCCCAAATTGGCTCAAAATGGGATGGTAGAAGTTATAGGTCTTGATTTTCTTTCATCTCATTACCATGCATTAGCAGCTATCCAAAGATTACTGACCGCAACGAATTACAAGGGGAACACAAAAGGGGTTGTTTTATCCAGAGAATCAAATAGTTTTCAATTTGAAGGATGGATACCAAGAATCCGTTTTACAAAAACTGAATTCTTAGAGGCTTATGGAGTTAAGCGGTATAAAACATCCAGAAATAAGTATGAGTTTAGTGGAAAAGAAGCTGAAACTGCTTTAGAAGCCTTATACCATTTAGGACATCAACCGTTTTTAATAGTGGCAACTAGAACTCGATGGACTAATGGAACACAAATAGTAGACCGTTACCAAACTCTTTCTCCGATCATTAGGATTTACGAAGGATGGGAAGGTTTAACTGACGAAGAAAATATAGATATAGACTTAACACCTTTTAATTCACCACCTACACGGAAACATAAAGGGTTCGTTGTAGAGCCATGTCCTATCTTGGTAGATCAAATAGAATCCTACTTTGTAATCAAGCCTGCAAATGTATACCAAGAAATAAAAATGCGTTTCCCAAATGCATCAAAGTATGCTTACACATTTATCGACTGGGTGATTACAGCAGCTGCGAAAAAGAGACGAAAATTAACTAAGGATAATTCTTGGCCAGAAAACTTGTTATTAAACGTTAACGTTAAAAGTCTTGCATATATTTTAAGGATGAATCGGTACATCTGTACAAGGAACTGGAAAAAAATCGAGTTAGCTATCGATAAATGTATAGAAATCGCCATTAAGCTTGGCTGGTTATCTAGAAGAAAACGCATTGAATTTCTGGATTCTTCTAAACTCTCTAAAAAAGAAATTCTATATCTAAATAAAGAGCGCTTTGAAGAAATAACTAAGAAATCTAAAGAACAAATGGAACAATTAGAACAAGAATCTATTAATTAATAGCAAGCTTGAAACTAAAAACCTAATTTATTTAAAGCTCAAAATAAAAAAGAGTTTTAAAATGGGAAATTCTGGTTTTTATTTGTATAACACTGAAAACTGCGTCTTTGCTGATAATATCAAAGTTGGGCAAATGACAGAGCCGCTCAAGGACCAGCAAATAATCCTTGGGACAACATCAACACCTGTCGCAGCCAAAATGACAGCTTCTGATGGAATATCTTTAACAGTCTCCAATAATTCATCAACCAATGCTTCTATTACAATTGGTTTGGATGCGGAAAAAGCTTACCAGCTTATTCTAGAAAAGTTGGGAGATCAAATTCTTGATGGAATTGCTGATACTATTGTTGATAGTACAGTCCAAGATATTTTAGACAAAATCAAAACAGACCCTTCTCTAGGTTTGTTGAAAGCTTTTAACAACTTTCCAATCACTAATAAAATTCAATGCAACGGGTTATTCACTCCCAGTAACATTGAAACTTTATTAGGAGGAACTGAAATAGGAAAATTCACAGTCACACCCAAAAGCTCTGGGAGCATGTTCTTAGTCTCAGCAGATATTATTGCATCAAGAATGGAAGGCGGCGTTGTTCTAGCTTTGGTACGAGAAGGTGATTCTAAGCCCTGCGCGATTAGTTATGGATACTCATCAGGCATTCCTAATTTATGTAGTCTAAGAACCAGTATTACTAATACAGGATTGACTCCGACAACGTATTCATTACGTGTAGGCGGTTTAGAAAGCGGTGTGGTATGGGTTAATGCCCTTTCTAATGGCAATGATATTTTAGGAATAACAAATACTTCTAATGTATCTTTTTTAGAGGTAATACCTCAAACAAACGCTTAAACAATTTTTATTGGATTTTTCTTATAGGTTTTATATTTAGAGAAAACAGTTCGAATTACGGGGTTTGTTATGCAAAATAAAAGAAAAGTGAGGGACGATTTTATTAAAATTGTTAAAGATGTGAAAAAAGATTTCCCCGAATTAGACCTAAAAATACGAGTAAACAAGGAAAAAGTAACTTTCTTAAATTCTCCCTTAGAACTCTACCATAAAAGTGTCTCACTAATTCTAGGACTGCTTCAACAAATAGAAAACTCTTTAGGATTATTCCCAGACTCTCCTGTTCTTGAAAAATTAGAGGATAACAGTTTAAAGCTAAAAAAGGCTTTGATTATGCTTATCTTGTCTAGAAAAGACATGTTTTCCAAGGCTGAATAGACAACTTACTCTAACGTTGGAGTTGATTTGCACACCTTAGTTTTTTGCTCTTTTAAGGGAGGAACTGGAAAAACAACACTTTCTCTAAACGTGGGATGCAACTTGGCCCAATTTTTAGGGAAAAAAGTGTTACTTGCTGACCTAGACCCGCAATCCAATTTATCTTCTGGATTGGGGGCTAGTGTCAGAAGTGACCAAAAAGGCTTGCACGACATAGTATACACATCAAACGATTTAAAATCAATCATTTGCGAAACAAAAAAAGATAGTGTGGACCTAATTCCTGCATCATTTTCATCCGAACAGTTTAGAGAATTGGATATTCATAGAGGACCTAGTAACAACTTAAAGTTATTTCTGAATGAGTACTGCGCTCCTTTTTATGACATCTGCATAATAGACACTCCACCTAGCCTAGGAGGGTTAACGAAAGAAGCTTTTGTTGCAGGAGACAAATTAATTGCTTGTTTAACTCCAGAACCTTTTTCTATTCTAGGGTTACAAAAGATACGTGAATTCTTAAGTTCGGTCGGAAAACCTGAAGAAGAACACATTCTTGGAATAGCTTTGTCTTTTTGGGATGATCGTAACTCGACTAACCAAATGTATATAGACATTATCGAGTCTATTTACAAAAACAAGCTTTTTTCAACAAAAATTCGTCGAGATATTTCTCTCAGCCGTTCTCTTCTTAAAGAAGATTCTGTAGCTAATGTCTATCCAAATTCTAGGGCCGCAGAAGATATTCTGAAGTTAACGCATGAAATAGCAAATATTTTGCATATCGAATATGAACGAGATTACTCTCAGAGGACAACGTGAACAAACTAAAAAAAGAAGCGGATGTCTTTTTTAAAAAAAATCAAACTGCCGCTTCTCTAGATTTTAAGAAGACGCTTCCCTCCATTGAACTATTCTCAGCAACTTTGAATTCTGAGGAAAGTCAGAGTTTGGATCGATTATTTTTATCAGAGTCCCAAAACTATTCGGATGAAGAATTTTATCAAGAAGACATCCTAGCGGTAAAACTGCTTACTGGTCAGATAAAATCCATACAGAAGCAACACGTACTTCTTTTAGGAGAAAAAATCTATAATGCTAGAAAAATCCTGAGTAAGGATCACTTCTCCTCAACAACTTTTTCATCTTGGATAGAGTTAGTTTTTAGAACTAAGTCTTCTGCTTACAATGCTCTTGCATATTACGAGCTTTTTATAAACCTCCCCAACCAAACTCTACAAAAAGAGTTTCAATCGATCCCCTATAAATCCGCATATATTTTGGCCGCTAGAAAAGGCGATTTAAAAACCAAGGTCGATGTGATAGGGAAAGTATGTGGAATGTCGAACTCATCGGCGATAAGGGTGTTGGATCAATTTCTTCCTTCATCTAGAAACAAAGACGTTAGAGAAACGATAGATAAGTCTGATTCAGAGAAGAATCGCCAATTATCTGATTTCTTAATAGAGATACTTCGCATCATGTGTTCCGGAGTTTCTTTGTCCTCCTATAACGAAAATCTTCTACAACAGCTTTTTGAACTTTTTAAGCAAAAGAGCTGATCCTCCGTCAGCTCATATATATATATCTATTATATATATATATTTAGGGATTTGATTTCACGAGAGAGATTTGCAACTCTTGGTGGTAGACTTTGCAACTCTTGGTGGTAGACTTTGCAACTCTTGGTGGTAGACTTTGCAACTCTTGGTGGTAGACTTGGTCATAATGGACTTTTGTTAAAAAATTTCTTAAAATCTTAGAGCTCCGATTTTGAATAGCTTTGGTTAAGAAAATGGGCTCGATGGCTTTCCATAAAAGTAGATTGTTTTTAACTTTTGGGGACGCGTCGGAAATTTGGTTATCTACTTTATCTTATCTAACTAGAAAAAATTATGCGTCTGGGATTAACTTTCTTGTTTCTTTAGAGATTCTGGATTTATCGGAAACCTTGATAAAGGCTATTTCTCTTGACCACAGCGAATCTTTGTTTAAAATCAAGTCTCTAGATGTTTTTAATGGAAAAGTTGTTTCAGAGGCATCTAAACAGGCTAGAGCGGCATGCTACATATCTTTCACAAAGTTTTTGTATAGATTGACCAAGGGATATATTAAACCCGCTATTCCATTGAAAGATTTTGGAAACACTACATTTTTTAAAATCCGAGACAAAATCAAAACAGAATCGATTTCTAAGCAGGAATGGACAGTTTTTTTTGAAGCGCTCCGGATAGTGAATTATAGAGACTATTTAATCGGTAAATTGATTGTACAAGGGATCC
