# Supplementary material for: Chlamydia trachomatis and Chlamydia muridarum spectinomycin resistant vectors and a transcriptional fluorescent reporter to monitor conversion from replicative to infectious bacteria
Source: PLoS One. 2019 Jun 6;14(6):e0217753. doi: 10.1371/journal.pone.0217753 (PMC6553856; doi:10.1371/journal.pone.0217753)
Supplement: S9 Fig — (DOCX) [file pone.0217753.s010.docx]

**Plasmid p2TK2_Spec_-Nigg mCh(Gro_L2_) GFP(OmcA_Cm_) Features**

*incDEFG* promoter: bases 1-228

*aadA* ORF (spectinomycin resistance): bases 229-1239

Terminator: bases 1240-1361

*E.coli* origin of replication: bases 1451-2234

*groESL* Promoter: bases 2256-2426

*mCherry* ORF: bases 2427-3137

*groESL* Terminator: bases 3138-3265

*C. muridarum omcA* Promoter: bases 3293-3619

*gfp* ORF: bases 3620-4351

*incDEFG* Terminator: bases 4352-4518

Unique Restriction Sites: KpnI (3287), NotI (4519), and SgrDI (4526)

Nigg: bases 4534-11662

**Plasmid p2TK2_Spec_-Nigg mCh(Gro_L2_) GFP(OmcA_Cm_) Sequence**

AACGGAGCCTTCTAGCTATTTTGTAAATATTTTAACAATTTAGATTCTTCAAAGCTCAGCGAGGGCGTGAAGAATCTTGTTCAGGTGTATTTGAAAAAAGTTTGTTTTAAATAGTTTTTTTAGTTAAAATGGGTCCCTAAATAATTTAAATCCGGTAGTTTTTGCGTCCGAAACATTGTTTTATAAGTGAGAAATGAGATCTGGCTAAAATCTGTCGAAGTGAGGTTTATGCGCTCACGCAACTGGTCCAGAACCTTGACCGAACGCAGCGGTGGTAACGGCGCAGTGGCGGTTTTCATGGCTTGTTATGACTGTTTTTTTGGGGTACAGTCTATGCCTCGGGCATCCAAGCAGCAAGCGCGTTACGCCGTGGGTCGATGTTTGATGTTATGGAGCAGCAACGATGTTACGCAGCAGGGCAGTCGCCCTAAAACAAAGTTAAACATCATGAGGGAAGCGGTGATCGCCGAAGTATCGACTCAACTATCAGAGGTAGTTGGCGTCATCGAGCGCCATCTCGAACCGACGTTGCTGGCCGTACATTTGTACGGCTCCGCAGTGGATGGCGGCCTGAAGCCACACAGTGATATTGATTTGCTGGTTACGGTGACCGTAAGGCTTGATGAAACAACGCGGCGAGCTTTGATCAACGACCTTTTGGAAACTTCGGCTTCCCCTGGAGAGAGCGAGATTCTCCGCGCTGTAGAAGTCACCATTGTTGTGCACGACGACATCATTCCGTGGCGTTATCCAGCTAAGCGCGAACTGCAATTTGGAGAATGGCAGCGCAATGACATTCTTGCAGGTATCTTCGAGCCAGCCACGATCGACATTGATCTGGCTATCTTGCTGACAAAAGCAAGAGAACATAGCGTTGCCTTGGTAGGTCCAGCGGCGGAGGAACTCTTTGATCCGGTTCCTGAACAGGATCTATTTGAGGCGCTAAATGAAACCTTAACGCTATGGAACTCGCCGCCCGACTGGGCTGGCGATGAGCGAAATGTAGTGCTTACGTTGTCCCGCATTTGGTACAGCGCAGTAACCGGCAAAATCGCGCCGAAGGATGTCGCTGCCGACTGGGCAATGGAGCGCCTGCCGGCCCAGTATCAGCCCGTCATACTTGAAGCTAGACAGGCTTATCTTGGACAAGAAGAAGATCGCTTGGCCTCGCGCGCAGATCAGTTGGAAGAATTTGTCCACTACGTGAAAGGCGAGATCACCAAGGTAGTCGGCAAATAAGCTAATTTTATTGCAATAACAGGTGCTTACTTTTAAAACTACTGATTTATTGATAAATATTGAACAATTTTTGGGAAGAATAAAGCGTCCTCTTGTGAAATTAGAGAACGCTTTATTACTTTAATTTAGTGAAACAATTTGTAACTACTGTCAGACCAAGTTTACTCATATATACTTTAGATTGATTTAAAACTTCATTTTTAATTTAAAAGGATCTAGGTGAAGATCCTTTTTGATAATCTCATGACCAAAATCCCTTAACGTGAGTTTTCGTTCCACTGAGCGTCAGACCCCGTAGAAAAGATCAAAGGATCTTCTTGAGATCCTTTTTTTCTGCGCGTAATCTGCTGCTTGCAAACAAAAAAACCACCGCTACCAGCGGTGGTTTGTTTGCCGGATCAAGAGCTACCAACTCTTTTTCCGAAGGTAACTGGCTTCAGCAGAGCGCAGATACCAAATACTGTCCTTCTAGTGTAGCCGTAGTTAGGCCACCACTTCAAGAACTCTGTAGCACCGCCTACATACCTCGCTCTGCTAATCCTGTTACCAGTGGCTGCTGCCAGTGGCGATAAGTCGTGTCTTACCGGGTTGGACTCAAGACGATAGTTACCGGATAAGGCGCAGCGGTCGGGCTGAACGGGGGGTTCGTGCACACAGCCCAGCTTGGAGCGAACGACCTACACCGAACTGAGATACCTACAGCGTGAGCTATGAGAAAGCGCCACGCTTCCCGAAGGGAGAAAGGCGGACAGGTATCCGGTAAGCGGCAGGGTCGGAACAGGAGAGCGCACGAGGGAGCTTCCAGGGGGAAACGCCTGGTATCTTTATAGTCCTGTCGGGTTTCGCCACCTCTGACTTGAGCGTCGATTTTTGTGATGCTCGTCAGGGGGGCGGAGCCTATGGAAAAACGCCAGCAACGCGGCCTTTTTACGGTTCCTGGCCTTTTGCTGGCCTTTTGCTCACATGTTCTTTCCTGCGTTATCCCCTGATTCTGTGGATAACCGTATTACACCGGTATTTTTAAAAATAGCAGTTGATCATGCCAACTGCTAAACCAGTTGCAAAAAAGCGAGGACTTTGCTATCGTTCTTCCTCTGAACGTTCTATCGTTCAAATCCCTACGTTGGTAGCGGAACAAAGCCGGACCACGGGGCCTCATAGAATATAAAAATACGAGGAGCTTAAACATGGTGAGCAAGGGCGAGGAGGATAACATGGCCATCATCAAGGAGTTCATGCGCTTCAAGGTGCACATGGAGGGCTCCGTGAACGGCCACGAGTTCGAGATCGAGGGCGAGGGCGAGGGCCGCCCCTACGAGGGCACCCAGACCGCCAAGCTGAAGGTGACCAAGGGTGGCCCCCTGCCCTTCGCCTGGGACATCCTGTCCCCTCAGTTCATGTACGGCTCCAAGGCCTACGTGAAGCACCCCGCCGACATCCCCGACTACTTGAAGCTGTCCTTCCCCGAGGGCTTCAAGTGGGAGCGCGTGATGAACTTCGAGGACGGCGGCGTGGTGACCGTGACCCAGGACTCCTCCCTGCAGGACGGCGAGTTCATCTACAAGGTGAAGCTGCGCGGCACCAACTTCCCCTCCGACGGCCCCGTAATGCAGAAGAAGACCATGGGCTGGGAGGCCTCCTCCGAGCGGATGTACCCCGAGGACGGCGCCCTGAAGGGCGAGATCAAGCAGAGGCTGAAGCTGAAGGACGGCGGCCACTACGACGCTGAGGTCAAGACCACCTACAAGGCCAAGAAGCCCGTGCAGCTGCCCGGCGCCTACAACGTCAACATCAAGTTGGACATCACCTCCCACAACGAGGACTACACCATCGTGGAACAGTACGAACGCGCCGAGGGCCGCCACTCCACCGGCGGCATGGACGAGCTGTACAAGTAGTTCCTCTAATGGGAACAAATAGATTCTTCGAGCCTCGTTTCCCAAAAGGAACGAGGCTTTTTTTTAGATTCCTAATATTTCTCTATTCCTCTATCGTAAACATCTAGTGCTTACGACCATCCTTTTCTGGATAACCGTATTACACCGGTGGTACCTTTTTTACCCCTTTGATTCTTTTATTATATAGTTAATTTGTTTGTTATCTATAGTTTAGGAATATTCTCTTTCGTTTTTATTTTTGTTTGTTTCTATCAACAGAATTTCATCTGTTTGAAATATAGTGATTTGACGCACATTGCTTTATAACTATTAATTAGTAACTATCTGTTATTAGATGATTCTTTTTAAAAAAGAATTGATATAATTTTTATTTTATAATGTAATATTGTCTGTGAGGGCCAGTTTCTTTTATTGGTAAAAGAATTGCTTTTGTTAATAAAAGAAACTTCGAGAGCCCTTTTCTAGAAAGGAGTCTGGAAGTTATGAGTAAAGGAGAAGCACTTTTCACTGGAGTTGTCCCAATTCTTGTTGAATTAGATGGTGATGTTAATGGGCACAAATTTTCTGTCAGTGGAGAGGGTGAAGGTGATGCAACATACGGAAAACTTACCCTTAAATTTATTTGCACTACTGGAAAACTACCTGTTCCATGGCCAACACTTGTCACTACTCTTACGTATGGTGTTCAATGCTTTTCAAGATACCCAGATCATATGAAACGGCATGACTTTTTCAAGAGTGCCATGCCCGAAGGTTATGTACAGGAAAGAACTATATTTTTCAAAGATGACGGGAACTACAAGACACGTGCTGAAGTCAAGTTTGAAGGTGATACCCTTGTTAATAGAATCGAGTTAAAAGGTATTGATTTTAAAGAAGATGGAAACATTCTTGGACACAAATTGGAATACAACTATAACTCACACAATGTATACATCATGGCAGACAAACAAAAGAATGGAATCAAAGTTAACTTCAAAATTAGACACAACATTGAAGATGGAAGCGTTCAACTAGCAGACCATTATCAACAAAATACTCCAATTGGCGATGGCCCTGTCCTTTTACCAGACAACCATTACCTGTCCACACAATCTGCCCTTTCGAAAGATCCCAACGAAAAGAGAGACCACATGGTCCTTCTTGAGTTTGTAACAGCTGCTGGGATTACACATGGCATGGATGAACTATACAAGTCCGGACTCAGATCTTAAGGATGACATGTGATTCGCGTAGGAAAAAGAGGAGGGAGACCTCCTCTTTTTTTTTATTTTGTAGAGTTCCGTTACTATTGGCACCCTGTGTGCAGTTAGGATGAGTAGACTAGTTCTGCAGCCTTTTACAGGGTGTTATGTTTTGCATTGCAAAAAGCTCCTAAGACGCGGCCGCGTCGACGGATCCGTTTGTTCTGGGGAAGAGGTAATTCCTCTAGTACAAACACCCACAATATTGTGATATAATTAAAATTATATCCATTTAGTTGCCCTCAAAAGCAACTGTAGATTATATTAGGGCCATCTTCTTTGAGGCATTGTCTTCTCTAGAGGATTTATCGTACGCAAATACCATCTTTGCGGTTGTGTGTCCTGTGACCTTCATGATGTCGGAGTCCGAACACCCTAGGCGTTTGTACTCTGTCACAGCGGTTGCTCTAAGCACGTGAGGGGTTATCTTAAATGGGATAGATGCTTGCAGTCCTGCTTGAGAGAACGTGCGGGCAATTTGTCTTAACCCCACCATTTTTCCAGAACTAGTTACGAAGACCAAACCTCTTCGTGGCCCAATGTACTCTCTTAGAGCGTGCATGAACTTCTGAGGATAAGTTATAATAATCCTCTTTTCTGTCTGACGATTCTTAAGCTGGGAGAAAGAGATAGTTGCTTGTTGAAAGCAGATCTGATCGATCTCTAAGCTTAAGACTTCAGAAGAACGCTTACCTCCTTGCAGCATAGTTTGGGCGATCAACCAATCTCTGGGATTGATTTTTTTTAGTTCTTTCAAGAAAGAAGCTGTTTGCAATCTATTCATTGCATTTGTTTTTACAATTTCTCTGGTTTTGAAAAATGTTCGGCTGTTTTCTTGTTTAGAAGGTTGTGCGATAGAAACAATTCCCTGAGTCATTCTGTTTAAAAATCTAGTCAAAGAGATATAACTAGCTGCACGAACTTGTTTGGTGCCTTCTGTCCATGAAGCTTTTGACGATGGAATCTTTTTAATTGCATCCAATATCAAGTTATGATTCAAAAGAGAAAATTCTTGTAGATTCATGTCTAAAGACAATAGCCCAATCTTTTCTAAAGCTAAAAAAGAGCCTCGGTAAGATCTACAAGTATGCTGATTTAGTGATGCAGTCCAATGCATGATAACTTCGAATAAAGAGAAGCTTCTCATGCGTTTCCAGTAAGATTCTTGTCGGATTTTTAATACTTCCTGATAAGACTTTCCGATATATTCTAATGGCATTTCTTGCTGCAAAGATAAAATCCCTTTACCCACGAAATTCCTCGTGATATAACCTAAACGCAAATGTCCTGATTAGTGAAATAATCAGGTTATCATTAGGATAGCACGCGCTGCATTTTTTTAGAAAAGCATGAAAACTAATTCTGAAATAGAAAACCGCATGCAAGATATTGAGTATGCGTTACTAGGAAAAGCTTTGGTATTTGAAGACTGTACAGAGTACATTCTTAGGCAACTTGTTAATTACGAATTCAAGTGCTCTCGCCATAAAAACATATTCATTGTTTTTAAACACTTAAAAGACAACGCTCTGCCAATAACTGTAGATTCAGCTTGGGAAGAGCTGTTAAGAAGGCGTGTCAAAGATATAGATAAGTCTTATCTCGGTATAATGTTACATGATGCCATGTTTAACGATAAGCTCAGGCCTATTTCGCATACGGTTCTTTTAGATGACTTAAGTGTATGTAGCGCTGAAGAAAATTTAACTAATTTCATTTTTCGTTCGTTTAATGAATATAACGAAAATCCATTGAGACGATCACCATTTTTACTATTAGACCGCATAAAAGATCGTCTCGACAGAACTATCGCAAAAACTTTTTCTACTCGTAGCGTTAGAGGACGATCTGTTTATGATATCTTTTCTCAAGCAGAACTCGGAGTATTAGCTCGTATAAAAAAAAGAAGGGCGGCTTATTCTGAGAATAATGATTCATTTTATGACGGCTTGCCAACCGGATATCAAGATATTGATAGTAAAGGGGTTATTTTAGCGAACGGCAATTTTGTGATAATTGCAGCTCGGCCTTCTATAGGGAAAACCGCACTCGCTATTGATATAGCTATCAATATTGCTATCCATCAACGACGTAGAGTTGGTTTTTTATCTCTTGAAATGAGTGCAGGGCAAATAGTTGAAAGAATTATTTCTAACTTAACAGGGGTATCTGGAGAGAAATTACAAAGGGGCTCTCTATCTGAAGAAGAGATTTTTTGCATTGAAGAAGCAGGAAATACTATAAGAGATTCTCATCTTTATATTTGTAGTGACAACCAATATAAGCTCAATTTGATAGCGAATCAAATTCGTTTGTTAAAACGAGATGATCGTGTCGACGTTATTTTTATCGATTACTTACAACTTATTAACTCATCTGTTGGAGAAAATCGACAAAATGAAATAGCAGATATATCTAGAACTTTAAGGGGGTTAGCTGCAGAGCTAAACATTCCTATAGTTTGTTTGTCTCAATTATCCAGAAAAGTCGAGGATAGAGCAAACAAAGTTCCTATGCTGTCAGACCTAAGAGATAGCGGTCAAATAGAACAGGATGCAGATGTAATTTTGTTCATCAATAGAAAGGAAACTTCTCCTAATTGTGAAATAACAGTGGGTAAAAATAGACATGGATCGGTTTTCTCTACTGTATTACAGTTCGATCCAAAAACAAGTAAGTTCTCTGCTATTAAAAAAGTATGGTAAATTATAGCAACTGTCACTTCATTAGAAGTCCTATTCATCTTGAGAATCAGAAGTTTGGTAGAAGACCAGGTCAATTAATCAAGATATCTCCTAAGTTAGCTCAAAATGGCTTAGTAGAAGTCATAGGTCTTGACTTTCTTTCTTCTCATTACCACGCACTAGCTGCTATCCAGAGATTACTTACAGCTACAAATTATAAGGGGAATACAAAAGGAGTTGTATTATCAAGAGAATCAAACAGCTTCCAATTCGAAGGTTGGATTCCTCGAATTAGATTTACAAAAACAGAGTTCTTAGAAGCTTACGGCGTAAAACGATACAAAACATCTAGAAACAAATACGAATTTAGTGGGAAAGAATCTGAAACAGCTTTAGAGGCTCTGTATCATTTAGGACATCAACCTTTCTTGATAGTGGCAACCAGAACTCGATGGAATAATGGGACGCCTATTTTAGATCGTTATCAAACCCTTTCGCCTATTATTAGAATTTACGAAGGATGGGAAGGTCTAACTGATGAAGAAAATACAGAAATTGATGTAACACCATTCAATTCACCATCAACACGAAAGCATAAAGGATTCATTGTAGAACCTTGTCCCATCTTGGTAGATCAAATAGACTCTTATTTCGTAGTCAAGCCTGCGAACGTATACCAAGAAATAAAAATGCGTTTCCCAAACGCATCAAGATATGCTTACACCTTTATTGATTGGATAATTACTGCATCTGCCAAAAAGAAAAGAAAATTGACCAAAGAGAATTCTTGGCCAGAAAACTTGTCTCTGAATGTTAACGTTAAAAGCCTTGCGTATATTTTAAGGATGAATCGATATATCAGCACAAGAAACTGGAAAAAAATTGAAATGGCTATTGATAAATGTGTTGAAATAGCTATTCAACTAGGTTGGTTATCTAGTCGGAAACGAGTAGAGTTCTTAGAAGCATCTAAGCTGTCTAAAAAAGAGATCTTGTATTTAAACAAAGAACGCTTTGAAGAAATAACAAGAAAATCAAAAGAACAAATGAATCAATTCGAGCAAGAATTTAATTAAAAAATAGCAAAACTTGAAACTAAAAACCAAATTTATTTAAAGCTCAAAATAAAAAGAGTTTTTAAAATGGGAAATTCTGGTTTTTATTTGCATAACACTAGCAACTGTGTATTTGCCGACAATATTAAAGTTGGGCAAATGACAGAACCTCTTACAGATCAACAAATAATACTTGGGACATCGACAACTCCTGTCGCAGCAAAAATAACAGCTTCTGAAGGGATATCCTTAACAATAACAAACAATGCTCAAGCTAACTCTTCAGTAAATATTGGATTAGATGCTGAAAAAGCGTACCAACTTATTTTAGATAAGCTTGGCGACCAAATCTTTGATGGAATCACAGGATCCATAGTTGAGAGTGCTGTACAGGACATTATAGATAAGATTACCTCGGACCCTTCTCTAGGATTGTTGAAGGCTTTCTATAACTTCCAAATCACTGGGAAAATTCAATGTAACGGCCTATTCACATCTAGCAATGTAACAACTTTATTAGGAGGAACAGAAATAGGTAGATTTACAGTAACTCCTAGAAGTTCTGGAAGCATGTTTTTAGTTTCTGCAGATATCATTGCATCAAGAATGGAAGGTGGAGTTGTATTAGCCTTAGTAAAAGAAGGAGATACACAACCATGTGCGATTAGCTATGGCTATTCTTCTGGTGTGCCCAATTTATGTAGCTTAAAAACCTGTGTTACTAATTCCGGATCGACACCCACAACTTATTCATTACGAATAGGAGGATTAGAGAGCGGAGTTGTATGGGTTAATGCTCTATCCAATGGTAATGATATTCTTGGAATAACAAATACTTCTAACGTTTCTTTTTTGGAGGTGATACCTCAAAAAAACACTTAAATAATTTTATTGGAATTTTCTTATCGGTTTTATATTTAGAAGAAACAGTTCTAATTACGGGGGTTGTTATGCAAAACAAAAGAAAACTGAGAAACGATTTTATTAAAATTGTTAAAGATGTAGAAAAGGATTTCCCCGAGCTAGACTTGAAAATACGGGTGAATAAGGAAAGGGTTACTTTTTTAAATTCACCCTTAGAACTCTACCACAAGAGTATTTCATTAATTTTAGGCTTGTTACAACAAATAGAAAAGTCTTTGGAATTATTTCCAGATTCCCCCGTTCTTGAAAAATTAGAGGATAACAGTTTAAAGCTAAAAAAAGCGTTGATTATGCTTATTCTGTCTAGAAAAGACATGTTTTCTAAGGCAGAATAGATGTTTTACTCTAACGTTGGAGTACACTTTGCAAACCTTAGTTTTTTGCTCTTTTAAGGGTGGGACAGGAAAAACAACACTTTCCCTGAATGTAGGGTGTAATTTAGCTCAATTTTTAGGAAAGAGAGTACTTCTAATTGACCTAGATCCCCAATCAAATCTCTCATCTGGATTGGGGGCTAGCATCGAAGGCAACCATAAAGGCCTTCACGAAGTGATGTGTGCCTCAAATGATTTAAAATCAATAATTTGTAAAACAAAAAAAACTGGGGTAGACATAATCCCTGCATCATTTTTGTCAGAACAATTTAGAGAATTTTCTACAAATGGCATCCCAAGCAGCAATTTACGGCTGTTTTTAGATGAGTATTGTTCGCCTTTATATGATGTGTGCATAGTAGATACTCCACCTAGTCTTGGTGGATTAACAAAAGAAGCCTTTATTGCAGGAGACAAACTAATCGTATGTTTGATTCCTGAGCCATTTTCTATTCTCGGGCTGCAGAAAATTAGAGAATTTTTAATTTCTATAGGCAAACCTGAGGAAGAACATATTCTTGGGGTAGCACTATCTTTTTGGGATGACCGGAGTTCGACTAATCAAACGTACATAGATATCATTGAGTCAATTTACGAAAATAAGATTTTTTCAACAAAAATACGCAGAGATATTTCTTTGAGTCGTTCCCTTCTTAAAGAGGATTCTGTGATCAATGTATATCCAACTTCAAGAGCTGCAACAGATATTCTGAATTTAACACACGAAATATCTGCTCTTTTAAATTCTAAACACAAACAAGACTTTTCCCAGAGGACACTGTGAATAAACTGGAAAAGGAAGCTAGCGTCTTTTTTAAAAAAAATCAGGAATCCGTTTCTCAAGACTTTAAGAAAAAGGTTTCTTCAATTGAGATGTTTTCAACTTCTTTAAATTCGGAGGAAAACCAGAGTCTGGATCGGCTTTTTTTGTCTGAGACTCAGAATTTATCAGATGAAGAATCTTACCAAGAAGATGTTTTGTCAGTAAAACTTCTGACAAGTCAAATAAAGGCTATTCAAAAACAACACGTGCTCCTTCTTGGAGAGAAGATTTACAATGCGAGAAAGATACTAAGTAAAAGTTGTTTCTCTTCAACAACCTTTTCATCTTGGCTAGATTTAGTTTTCAGGACTAAATCATCCGCCTATAATGCGTTGGCTTATTATGAACTTTTCATAAGTCTACCAAGCACAACTTTGCAGAAAGAGTTCCAATCAATCCCGTATAAGTCTGCATATATTTTAGCTGCTAGGAAAGGAGACTTAAAAACAAAAGTCTCTGTTATAGGGAAAGTTTGTGGAATGTCCAATGCATCTGCTATCCGGGTTATGGACCAACTTCTTCCTTCATCTAGAAGTAAAGATAATCAAAGATTTTTCGAATCTGATTTAGAGAAAAATCGACAGTTATCAGATCTTCTCGTAGAACTGCTTCGCATTGTATGTTCTGGAGTTTTCTTATCTCCTTATAACGAAAACCTTCTGCAGCAGTTGTTTGAAGTCTATAAGCAAAAGAGCTGATCCGCCGTCAGCTCTTATATATATATCTATTATATATATATATTTTAGGGATTCGATTTTACGAGAGCTTCGCGCAACTCTTGGTGGTAGACCTTGCAACTCTTGGTGGTAGACCTTGCAACTCTTGGTGGTAGACCTTGCAACTCTTGGTGGTAGACTTAGTCGGGATAGACTTTTGTGTAAAAAAAAAATAAACTCTTGAGACTCTGAATCAGAGTCATATTGTTTAAGAAAAGATGAACTCAAAATTTTACCACAGAAGTAGGCTATTCCTAACTTTTGGAGACGCGTCGGAAATTTGGTTATCTACTTTATCTTATCTAACTAGAAAAAATTATGCGTCTGGGATTAACTTTCTTGTTTCTTTAGAGATTCTGGATTTATCGGAAACCTTGATAAAGGCTATTTCTCTTGACCACAGCGAATCTTTGTTTAAAATCAAGTCTCTAGATGTTTTTAATGGAAAAGTTGTTTCAGAGGCATCTAAACAGGCTAGAGCGGCATGCTACATATCTTTCACAAAGTTTTTGTATAGATTGACCAAGGGATATATTAAACCCGCTATTCCATTGAAAGATTTTGGAAACACTACATTTTTTAAAATCCGAGACAAAATCAAAACAGAATCGATTTCTAAGCAGGAATGGACAGTTTTTTTTGAAGCGCTCCGGATAGTGAATTATAGAGACTATTTAATCGGTAAATTGATTGTACAAGGGATCC
